# Depletion of Mageb16 induces differentiation of pluripotent stem cells predominantly into mesodermal derivatives

**DOI:** 10.1038/s41598-017-14561-z

**Published:** 2017-10-27

**Authors:** John Antonydas Gaspar, Sureshkumar Perumal Srinivasan, Poornima Sureshkumar, Michael Xavier Doss, Jürgen Hescheler, Symeon Papadopoulos, Agapios Sachinidis

**Affiliations:** 1 University of Cologne (UKK), Institute of Neurophysiology and Center for Molecular Medicine Cologne (CMMC), Robert-Koch-Str. 39, 50931 Cologne, Germany; 2 University of Cologne, Center of Physiology and Pathophysiology, Institute of Vegetative Physiology, Robert-Koch-Str. 39, 50931 Cologne, Germany

## Abstract

The Melanoma-associated Antigen gene family (MAGE) generally encodes for tumour antigens. We had identified that one of the MAGE gene members, Mageb16 was highly expressed in undifferentiated murine embryonic stem cells (ESCs). While the role of Mageb16 in stemness and differentiation of pluripotent stem cells is completely unknown, here, in our current study, we have demonstrated that Mageb16 (41 kDa) is distributed in cytosol and/or in surface membrane in undifferentiated ESCs. A transcriptome study performed at  differentiated short hairpin RNA (shRNA)-mediated Mageb16 knockdown (KD) ESCs and scrambled control (SCR) ESCs until a period of 22 days, revealed that Mageb16 KD ESCs mainly differentiated towards cells expressing mesodermal and cardiovascular lineage - gene markers. Gene markers of other mesoderm-oriented biological processes such as adipogenesis, osteogenesis, limb morphogenesis and spermatogenesis were also significantly enriched in the differentiated Mageb16 KD ESCs. The expression levels of contractile genes were higher in differentiated Mageb16 KD ESCs when compared to differentiated SCR and wild ESCs, suggesting a higher cardiomyogenic potential of Mageb16 depleted ESCs. Further analysis indicates  that regulative epigenetic networks and nucleocytoplasmic modifications induced by the depletion of Mageb16, may play a probable role in differentiation.

## Introduction

Melanoma-associated Antigen family (*MAGE*) of genes are located in X chromosome and encodes for tumour antigens which normally bind to T-lymphocytes in cancer patients^1^. The *MAGE* family genes are classified into type I and type II. Type I *MAGE* genes are further divided into subfamilies *MAGE-A*, *-B* and *-C* which are silent in almost all normal tissues except in testes and placenta. Interestingly, the type I *MAGE* genes are also highly expressed in rapidly proliferating cells such as tumour cells and in developing embryos^2^. The type II *MAGE* genes (*MAGE D1-D4, E, F, G* and *H*) are highly conserved and expressed in many adult tissues as well as in many tissues during embryonic development^3,4^. Homologous genes have also been identified in human, mice, and zebrafish^2^. Human *MAGEB16* belongs to the *MAGE B* subfamily (GenBank accession No.: NM_001099921.1) which have been identified based on the sequence and the isoelectric points of analogous proteins^5^. When verified for protein similarities between murine *Mageb16* and human *MAGEB16*, only 52% residue identity has been observed. Among the *MAGE* B subfamily, subgroups of genes- B1, B2, B3 and B4 share 70–80% of nucleotide identity and 49–68% of amino acid residue identity^5^. Recently, it has been reported that amongst 12 different human and mice tissues, the expression level of *MAGEB16* was very high only in the testis^6^. Authors have also reported very high expression levels of *Mageb16* in testis of 3 to 18 day postnatal mice embryos and though slightly decreased, the expression level was still high in testis of 4 month old adult mice^6^. Authors proposed that the testis-specific expression of *MAGEB16* in human and *Mageb16* in mice is regulated by the CpG methylation status of their promoter regions^6^.

In order to identify novel gene networks controlling fundamental biological processes of pluripotency and differentiation in murine ESCs, we recently performed microarray analysis of undifferentiated, early and late differentiated ESCs. Our transcriptome data showed that the most critical early differentiation processes occurs at day 2 and 3 of differentiation^7^. Besides monitoring well-annotated genes involved in regulation of pluripotency, germ layer formation and late differentiation processes toward somatic cell lines, *Mageb16* has been identified showing a similar time kinetic of expression like the pluripotency factors *Oct4* and *Nanog* ^7^. The expression level of *Mageb16* was very high in undifferentiated ESCs which considerably declined immediately after differentiation^7^.

Interestingly, siRNA knockdown of *Mageb16* in undifferentiated ESCs cultured under monolayer conditions resulted in a remarkable upregulation of mesodermal, ectodermal and endodermal marker genes after 48 h of differentiation as compared to untreated and control scrambled (SCR)-oligonucleotide treated ESCs^7^. These results suggested a crucial key role of *Mageb16* for maintenance of pluripotency and differentiation of ESCs. In the present study, this hypothesis has been evaluated by generating a transgenic mESC line in which *Mageb16* was permanently silenced by knockdown (KD) of *Mageb16* using a shRNA directed to *Mageb16* mRNA. Moreover, for a detailed study of its functional role in controlling pluripotency and differentiation *Mageb16* KD ESCs were randomly differentiated for different time points using the embryoid body (EB) methodology. Signal transduction pathways affected by the depletion of *Mageb16* were identified by detailed transcriptome and bioinformatics analysis. Additionally, the cellular localization of Mageb16 has been determined in ESCs.

## Results

### Knockdown efficiency of *Mageb16* in ESCs

**Figure 1:**
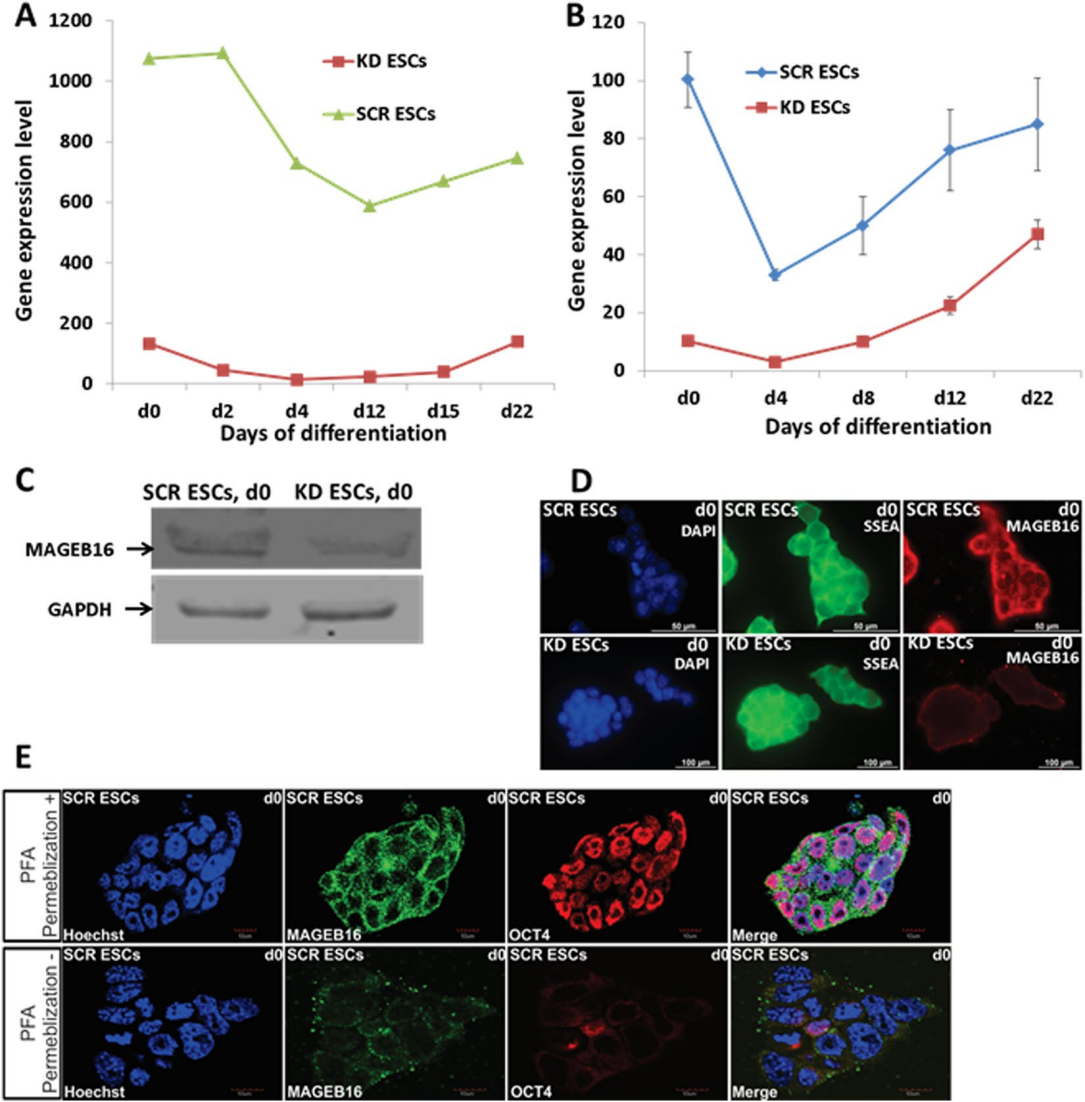
*Mageb16* expression in differentiated KD and SCR ESCs. (**A**) *Mageb16* expression in undifferentiated and differentiated KD and SCR ESCs (microarray data). (**B**) qPCR analysis of *Mageb16* expression in undifferentiated and differentiated KD and SCR ESCs (2 to 22 day EBs). (**C**) MAGEB16 protein expression in the KD and SCR ESCs. After preparation of the protein lysates, 10 µg protein was analysed by western blotting. Chemiluminescence detection of MAGEB16 has been performed using MAGEB16 polyclonal antibodies (1:250) and GAPDH has been detected using the anti-GAPDH antibody (1:2500) dilutions. (**D**) Cellular localization of MAGEB16 and SSEA1 in SCR and KD ESCs. Immunocytochemistry has been performed using primary anti-SSEA1 andibodies (1:50) (green colour) and anti-MAGEB16 antibodies (1:50) (red colour) and goat anti mouse IgM-alexa fluor 488 secondary antibody (1:1000) and goat anti rabbit IgG-alexa fluor 568 as secondary antibody (1: 1000). Cells were co-stained with nuclear marker Hoechst 33342. The overlay of nuclear and MAGEB16 staining reveals that the presence of MAGEB16 is restricted to cytosol and/or surface membrane (scale bar: 100 µm). (**E**) Confocal microscopy (upper panel+: permebilized cells; lower panel−: non-permebilized). Immunocytochemistry was performed using primary anti-OCT4 antibodies (1:250) (red color) and anti-MAGEB16 antibodies (1:50) (green color) and goat anti rabbit IgM-alexa fluor 488 secondary antibody (1:1000) and goat anti mouse IgG-alexa fluor 568 as secondary antibody (1:1000). Cells were co-stained with nuclear marker Hoechst 33342. The overlay of nuclear, OCT4 and MAGEB16 staining reveals the presence of MAGEB16, which is restricted to cytoplasmic domains of the ESCs (scale bar: 10 µm).

Maximal expression of *Mageb16* has been observed in undifferentiated SCR ESCs and the expression started declining at 4-day old embryoid bodies (EBs) (Fig. 1A) (Microarray data). The expression of *Mageb16* was drastically reduced in KD ESCs and remained low until 22-days in EBs (Fig. 1A). The efficiency of the *Mageb16* knockdown has been additionally confirmed by qPCR and western blot (independent experiments from microarray experiments). As indicated in Fig. 1B, the expression level of *Mageb16* in the SCR ESCs was very high (=100%) and declined by 30, 50 and 75% at 4-, 8-, and 22-days in SCR EBs, respectively. In comparison to SCR EBs, the *MageB16* mRNA expression level was reduced by more than 90% in KD ESCs and the expression level remained significantly lower in 4-, 8-, and 12-days KD EBs. Knockdown of *Mageb16* has also been confirmed at the protein level as shown by western blot analysis (Fig. 1C). MAGEB16 was distributed in cytosol and the cell surface of undifferentiated control SCR ESCs but not in the nucleus (Fig. 1D). No visible Mageb16 was observed in KD ESCs (Fig. 1D). As expected, Ssea1 a cell surface protein associated with undifferentiated ESCs^8^, was expressed in both undifferentiated SCR and KD ESCs. Confocal imaging of permeabilized and non-permeablized SCR ESCs, further confirmed the localization of MAGEB16 in cytosol and the cell plasma membrane (supplementary Figure 1E).

### Gene expression modulations regulated by the depleted Mageb16 during differentiation

**Figure 2:**
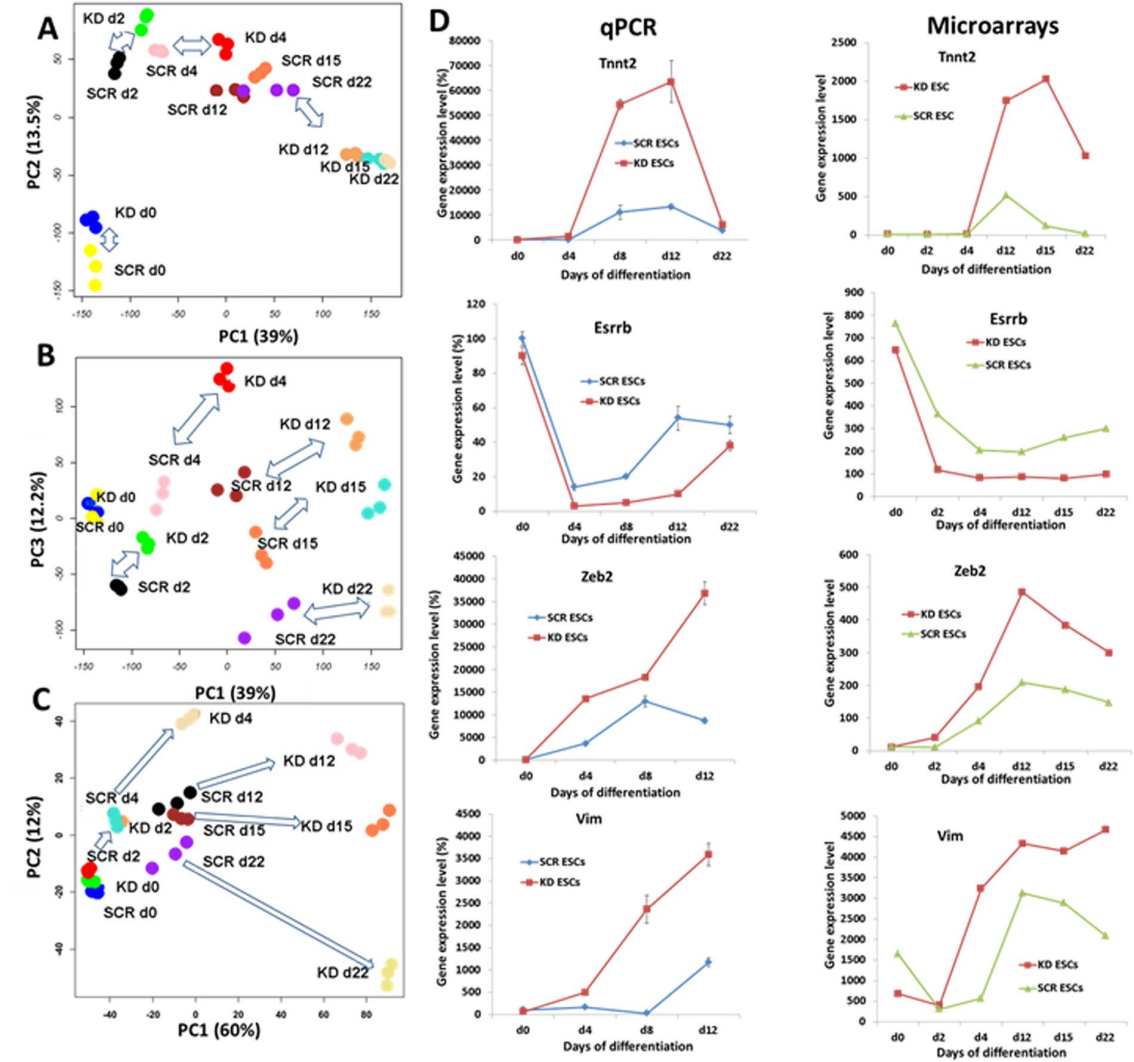
Principal Component Analysis of the entire normalized array datasets. After normalization of the entire transcriptome dataset obtained from both of the undifferentiated and differentiated SCR and *Mageb16* KD ESCs (2-day to 22-day EBs), they were analysed by principal component analysis (PCA). Each sphere represents individual sample from a color-coded triplicate sample. (**A**) PC1 shows the main variability among the transcriptome differences and PC2 shows the second largest variability. (**B**) PCA plot with PC1 and PC3 to monitor a clear separation at the third largest variance (PC3). (**C**) PCA of the significantly differential expressed genes (at least 2 fold; FDR corrected P value < 0.05) between undifferentiated and differentiated SCR or undifferentiated and differentiated KD ESCs. A PC1 and PC2 variance of 60% and 12%, respectively, has been obtained indicating a clear separation between the transcriptomes of the differentiated SCR and KD ESCs. (**D**) Gene expression of representative genes determined by qPCR analysis. The gene expression data of triplicates for each experimental condition are expressed as mean ± SD (*P < 0.05 for KD 12-day versus control SCR 12-day EBs). The expression of *Mabeb16* determined by qPCR is shown in Fig. 1B.

Principal component analysis (PCA) of the transcriptomes was performed for- undifferentiated Mageb16 KD ESCs, SCR ESCs and differentiated (2-, 4-, 12-, 15- and 22 days) EBs of both the populations (Fig. 2). The PCA plot of the analysis of the entire transcriptome of the undifferentiated and differentiated ESC populations and EBs of both Mageb16 depleted and SCR cell types indicates a 39% variance in the principal component 1 PC1,13.5% variance in PC2 and 12.2% in PC3 (Fig. 1A and B). A Clear separation between undifferentiated SCR ESCs and undifferentiated Mageb16 KD ESCs was obvious in PC2 (Fig. 2A). The significant distance between the different differentiation time points of both the populations were seen clearly at PC3 with 12.3% of variance. Using the significance of change FDR corrected P value < 0.05 and size of change at least 2 fold of the gene expression part of statistical correction, we determined 4499 statistically significant expressed probe sets doing statistical extensive comparisons of different cell populations of our study. Among the probe sets there were 174 non-annotated, 225 Riken cDNAs and 3060 annotated genes. As shown in Fig. 2C, significant transcriptome of differentiated KD ESCs and EBs shows clear variance of its earlier expression foldaway compared to control SCR ESCs and EBs. The main difference of a 60% variance was observed in PC 1 whereas 12% variance has been observed in PC2 (in total 72% variance). Validation of microarray data has been performed by choosing five genes (including *Mageb16*, see Fig. 1A, B) applying qPCR. As shown in Fig. 2D, qPCR data for expression of *Tnnt2*, *Esrrb*, *Zeb2* and *Vim* were consistent with microarray gene expression data (To be considered is that the qPCR validation of *Vim* and *Zeb2* has been performed for undifferentiated SCR and KD ESCs and 4-, 8- and 12-day SCR and KD EBs).

### K-means clusters and GO analysis

**Figure 3:**
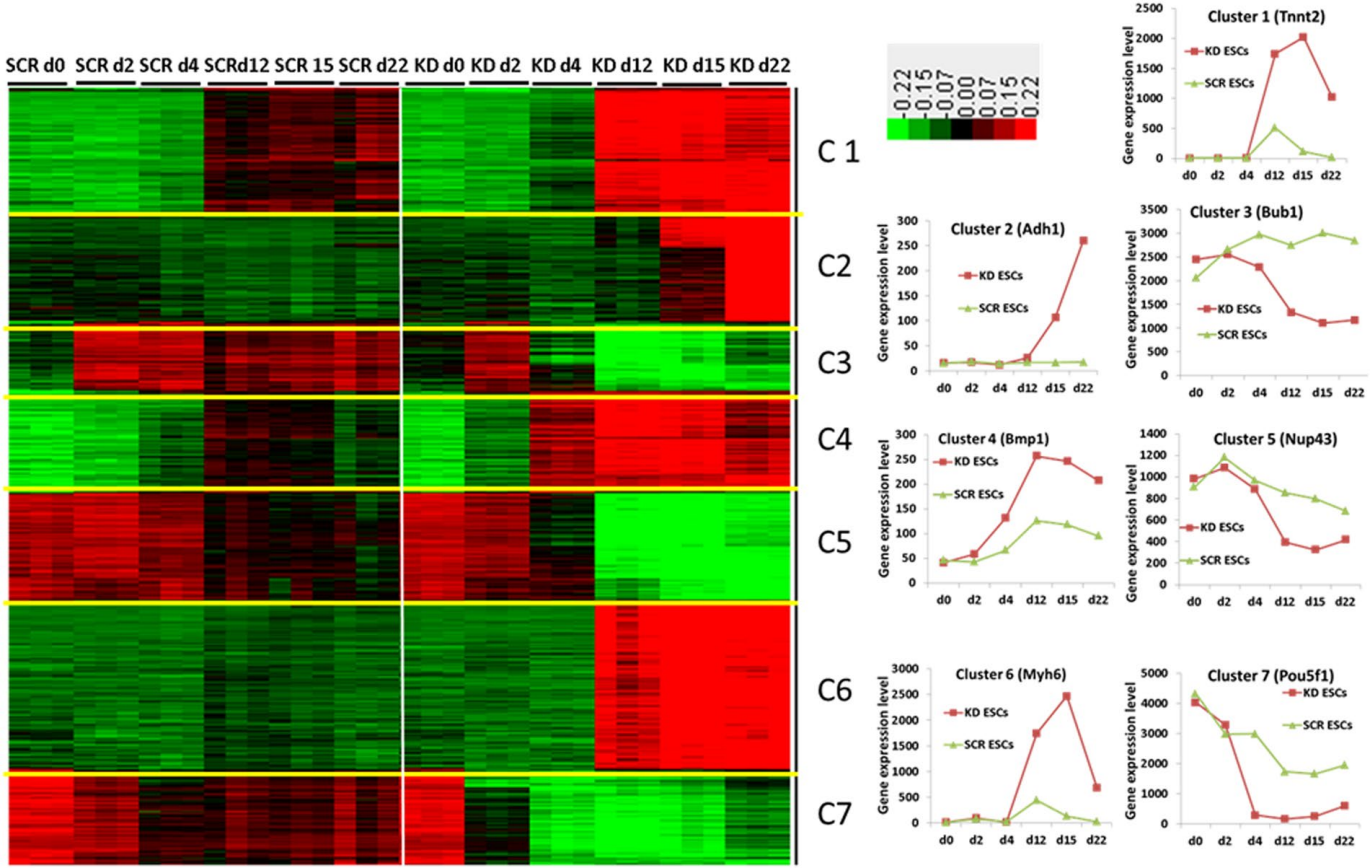
Visualization of k-means clustering of differentially expressed probe sets with Euclidean distance measurement and k = 7 group clusters. Replicates are displayed in the vertical axis and genes in the horizontal axis. Log2 transformed signal intensities are depicted in colour code. The heatmap indicates high expression levels in red, intermediate expression level in dark grey and low expression levels in green. Representative diagrams for the gene expression pattern of the different clusters are shown on the right.

Part of Gene set enrichment analysis, applying the k-means clustering, significantly altered genes (exhibiting at least 2-fold regulation in each condition) were clustered in seven specific gene clusters. The clustering was based on their expression pattern over the different differentiation time points (Fig. 3). Also illustrated on Fig. 3 (right) are the expression pattern of representative genes from the different clusters. To identify specific Gene Ontology (GO), biological processes (BPs), molecular function (MF) and cellular component (CC) along with KEGG pathways, the differentially expressed genes were analysed using Database for Annotation, Visualization and Integrated Discovery (DAVID) (http://david.abcc.ncifcrf.gov/) tool. The most biologically significant “parent” terms and more specialized “child” terms of the biological processes of 7 clusters are shown in Table 1. The complete GO analysis is shown in the Supplementary Dataset File.

**Table 1:** Specific GO and KEGG pathways of the cluster specific genes indicated in Fig. 3.

Term	Gene Nr.	PValue
Cluster 1		
GO:0009888~tissue development	49	7.76E-12
GO:0001568~blood vessel development	24	5.57E-08
GO:0001944~vasculature development	24	8.69E-08
GO:0048706~embryonic skeletal system development	14	1.43E-07
GO:0060348~bone development	15	1.58E-06
GO:0051216~cartilage development	12	3.86E-06
GO:0007420~brain development	23	3.90E-06
GO:0001649~osteoblast differentiation	9	2.02E-05
GO:0007417~central nervous system development	25	2.20E-05
GO:0030900~forebrain development	15	8.57E-05
mmu05414:Dilated cardiomyopathy	11	2.16E-04
GO:0060485~mesenchyme development	8	2.41E-04
GO:0043009~chordate embryonic development	24	4.31E-04
GO:0030324~lung development	10	0.0019
GO:0050767~regulation of neurogenesis	10	0.0062
GO:0001654~eye development	11	0.0065
GO:0030902~hindbrain development	7	0.0067
**Cluster 4**		
GO:0009888~tissue development	46	1.87E-13
GO:0001568~blood vessel development	27	4.74E-12
GO:0001944~vasculature development	27	8.28E-12
GO:0007507~heart development	22	5.64E-09
GO:0060429~epithelium development	23	3.70E-08
GO:0030324~lung development	13	2.71E-06
GO:0060485~mesenchyme development	9	7.00E-06
GO:0048732~gland development	16	1.27E-05
GO:0007517~muscle organ development	15	1.51E-05
GO:0009952~anterior/posterior pattern formation	14	1.52E-05
GO:0014032~neural crest cell development	7	4.80E-05
mmu04510:Focal adhesion	14	6.91E-05
GO:0022008~neurogenesis	26	1.07E-04
GO:0060348~bone development	11	1.51E-04
GO:0001656~metanephros development	8	1.70E-04
GO:0030326~embryonic limb morphogenesis	9	8.25E-04
GO:0007420~brain development	16	8.65E04
**Cluster 2**		
mmu00982:Drug metabolism	23	3.08E-18
mmu00980:Metabolism of xenobiotics by cytochrome P450	19	1.90E-14
mmu00830:Retinol metabolism	19	3.39E-14
GO:0009888~tissue development	34	1.14E-07
GO:0060429~epithelium development	21	1.92E-07
mmu00140:Steroid hormone biosynthesis	10	1.50E-06
mmu00983:Drug metabolism	10	2.66E-06
GO:0008202~steroid metabolic process	14	1.08E-05
mmu00590:Arachidonic acid metabolism	10	2.43E-04
GO:0008544~epidermis development	10	5.85E-04
GO:0007398~ectoderm development	10	9.15E-04
**Cluster 3**		
GO:0007126~meiosis	11	5.73E-07
GO:0005634~nucleus	89	7.74E-06
GO:0000279~M phase	15	6.85E-05
GO:0004089~carbonate dehydratase activity	5	1.02E-04
mmu00270:Cysteine and methionine metabolism	5	7.46E-04
GO:0000775~chromosome, centromeric region	8	8.26E-04
GO:0007283~spermatogenesis	10	0.0125
GO:0006006~glucose metabolic process	7	0.0166
mmu00480:Glutathione metabolism	4	0.0279
**Cluster 5**		
GO:0005634~nucleus	208	2.83E-40
GO:0006259~DNA metabolic process	57	1.26E-27
GO:0000279~M phase	46	1.66E-25
GO:0005730~nucleolus	37	1.60E-16
mmu04110:Cell cycle	17	2.62E-09
mmu03030:DNA replication	8	4.07E-06
GO:0006310~DNA recombination	11	7.53E-06
GO:0007126~meiosis	11	3.16E-05
GO:0051327~M phase of meiotic cell cycle	11	3.16E-05
mmu04115:p53 signaling pathway	9	5.43E-05
GO:0005654~nucleoplasm	28	3.18E-04
GO:0034470~ncRNA processing	12	0.001
GO:0040029~regulation of gene expression, epigenetic	7	0.005
GO:0006913~nucleocytoplasmic transport	8	0.006
GO:0046930~pore complex	6	0.022
GO:0007498~mesoderm development	5	0.045
mmu00051:Fructose and mannose metabolism	5	0.005
**Cluster 6**		
mmu04610:Complement and coagulation cascades	31	8.09E-21
GO:0002526~acute inflammatory response	26	2.41E-17
GO:0007596~blood coagulation	22	1.83E-14
GO:0019724~B cell mediated immunity	16	5.99E-09
GO:0043292~contractile fiber	18	1.85E-08
GO:0060429~epithelium development	28	1.35E-06
GO:0001944~vasculature development	24	2.98E-05
GO:0019752~carboxylic acid metabolic process	35	1.00E-04
GO:0048732~gland development	17	0.0018
GO:0050873~brown fat cell differentiation	6	0.0032
GO:0007398~ectoderm development	12	0.0079
GO:0031016~pancreas development	5	0.041
**Cluster 7**		
GO:0019827~stem cell maintenance	9	2.69E-09
GO:0048864~stem cell development	9	4.07E-09
GO:0005634~nucleus	115	1.94E-07
GO:0010468~regulation of gene expression	73	5.93E-06
GO:0007283~spermatogenesis	16	6.50E-05
GO:0007498~mesoderm development	6	0.004
GO:0007398~ectoderm development	6	0.095
GO:0007492~endoderm development	3	0.096

As indicated in Table 1, in comparison to the differentiated SCR ESCs, terms associated with tissue development were significantly enriched in Mageb16 depleted 12-, 15- and 22-day EBs (Fig. 3, Cluster 1). The most prominent BPs were mainly associated with development of mesoderm derivatives such as GO:0001568~blood vessel development, GO:0001944~vasculature development, GO:0048706~embryonic skeletal system development, GO:0051216~cartilage development and GO:0001649~osteoblast differentiation. The second set of most prominent BPs were associated with development of ectodermal derivatives such as GO:0007420~brain development, GO:0007417~central nervous system development and GO:0060429~epithelium development. Similarly, highly significant developmental GOs participating in the development of mesoderm derivatives have also been observed by the analysis of Cluster 4 genes indicating higher expression levels in 4-, 12-, 15-, and 22 days KD EBs in comparison to the differentiated SCR ESCs. This is followed by development of ectoderm derivatives such as GO:0007420~brain development, GO:0007417~central nervous system development and gland development. Analysis of the cluster 2 genes which were highly upregulated in the 22 days KD EBs in comparison to 22 days SCR EBs, indicated metabolism as the main KEGG (mmu00982: Drug metabolism) with the enriched biological GO, GO:0070330~aromatase activity. It included several “child” metabolism associated processes such as GO:0006631~fatty acid metabolic process, mmu00590:Arachidonic acid metabolism and other. Additionally, epithelium-, epidermis-, ectoderm-, gland-, brown fat cell- and lung development have also been identified in the Cluster 2 genes. In comparison to the 12-, 15- and 22-day SCR EBs, cluster 3 genes indicated a low expression level in the appropriate 12-, 15- and 22-day KD EBs. A further analysis indicated that BPs associated with the process of meiosis (GO:0007126~meiosis) and spermatogenesis (*Dnmt3a, Rec8, Tex15, Sycp3, Tcfl5, Hsf2, Nr6a1, Mael, Rad18, Nlrp14)* were significantly repressed (see Table 1 and Supplementary Dataset File).

Cluster 5 genes showed similar expression levels in KD ESCs, 2-, and 4-day KD EBs in comparison to the SCR ESCs, 2-, and 4-day SCR EBs. In this context, expression of genes belonging to the GO:0006259~DNA metabolic process, GO:0000279~M phase, GO:0005634~nucleus and GO:0005730~nucleolus were significantly repressed in the differentiated 12-, 15- and 22-day KD EBs in comparison to 12-, 15- and 22-day SCR EBs.

**Figure 4:**
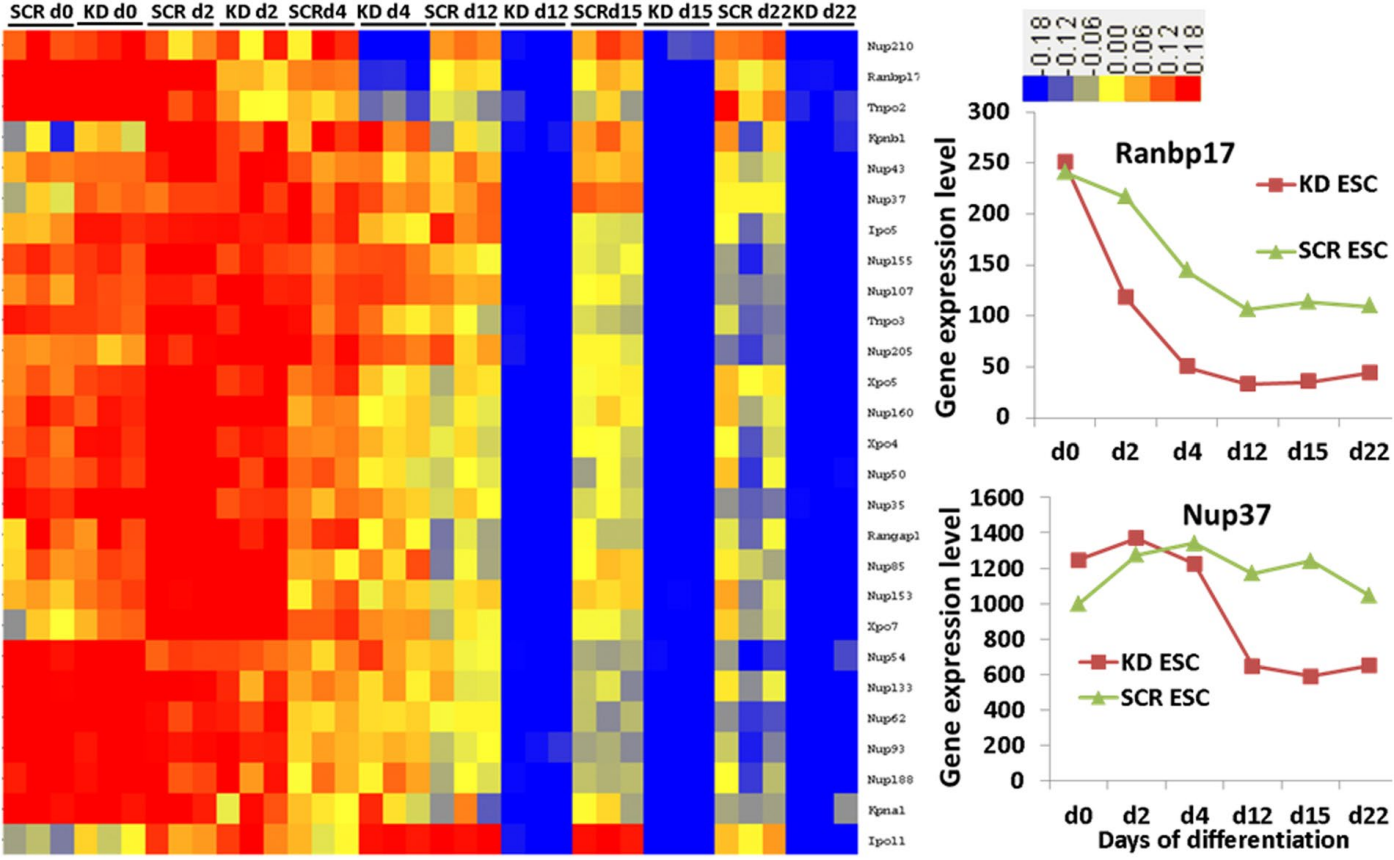
Visualization of differentially expressed genes participating in nucleocytoplasmic trafficking. Replicates are displayed in the vertical axis and genes in the horizontal axis. Log2 transformed signal intensities are depicted in colour code. The heatmap indicates high expression levels in red, intermediate expression level in yellow and low expression levels in blue. Representative diagrams on the right show the gene expression of the *Ranbp17* and *Nup37*.

Interestingly, genes belonging to the GO:0007498~mesoderm, GO:0031080~Nup107–160 complex, GO:0006913~nucleocytoplasmic transport (8 genes) and GO:0034470~ncRNA processing, GOTERM_BP_5,GO:0040029~regulation of gene expression and genes involved in epigenetics (*Xist, Hat1, Lin28a, Dnmt3b, Baz2a, Brca1, Hells*) are also included in cluster 5 genes (Fig. 3, Table 1). The expression pattern of nucleocytoplasmic transport and nuclear pore genes in cluster 5, and in addition, other well annotated genes belonging to these GOs, are shown in Fig. 4. As indicated, gene expression level remained high during differentiation of the KD ESCs and later time points of differentiation in comparison to the SCR EBs. In comparison to SCR ESCs and EBs, several nucleocytoplasmic transport and nuclear pore genes were downregulated after 12-days of differentiation in KD ESCs and EBs (Fig. 4). Some of the genes identified among them are *Ranbp17*, *Nup43*, *Nup133*, *Nup37, Kpnb1* and *Kpna1*. The absolute expression levels of *Ranbp17* and *Nup37* at the different differentiation time points are also shown in Fig. 4(right).

A similar gene expression pattern to cluster 5 has been observed in cluster 7 genes. The differences between the expression level of differentiated KD and SCR ESCs were obvious at 4-day EBs becoming more prominent with increasing time of differentiation. Interestingly, genes belonging to the GO:0019827~stem cell maintenance and GO:0007498~mesoderm development indicated lower expression level in 4 to 22-day KD EBs in comparison to the 4 to 22-day SCR EBs. The comparative expression of a few select genes from these 3 clusters- cluster 3, 5 and 7 is illustrated in the Supplementary Information, Figure S1.

Analysis of cluster 6 genes, which possess higher expression, levels in 12-, 15- and 22 days KD EBs as compared to SCR EBs identified mainly GOs and KEGGs associated with development of mesoderm derivatives. Among them were mmu04610:Complement and coagulation cascades, GO:0001568~blood vessel development, GO:0050873~brown fat cell differentiation, GO:0048732~gland development, GO:0031016~pancreas development and those responsible for functional processes of the heart have been identified.

### Expression of key genes associated with the contractility of cardiomyocytes

In order to figure out whether Mageb16 depletion resulted in impaired cardiomyogenesis, (a process that is driven by the mesoderm) we plotted the expression values of key contractility genes such as *Tnnt2* (Troponin T2), *Actc1* (Alpha-Cardiac Actin), *Myl2* (Cardiac Ventricular Myosin Light Chain 2), *Myl3*, (Cardiac Myosin Light Chain 1), *Myh6* (Myosin Heavy Chain, Cardiac Muscle Alpha Isoform) and *Myh7* (Myosin Heavy Chain, Cardiac Muscle Beta Isoform). As shown in Figure S2, there were no significant differences in the time kinetic in differentiating ESCs and SCR. However, the expression levels of the contractility genes in KD ESCs were significantly higher in differentiating KD ESCs. In addition, we monitored the contractile foci of 13-days SCR and KD EBs, which exhibit more contractile foci in 12-days KD EBs (see representative videos).

## Discussion

The discovery of novel molecular mechanisms controlling embryogenesis under *in vivo* conditions is problematic, cost-intensive and a time-consuming procedure requiring comprehensive animal studies. Nevertheless the developmental network can be partly recapitulated *in vitro* by the use of cultured ESCs which can be applied to recognize critical developmental processes. Recently, we have identified *Mageb16* as a novel regulator of pluripotency and differentiation in ESCs^7^. In this context, we have demonstrated that siRNA knockdown of *Mageb16*- a highly expressed gene marker in the pluripotent state of ESCs, resulted in a high expression of gene markers for all three germ layer- T bra (mesoderm), FGF5 (ectoderm), and HNF4a (endoderm) after 48 h. According to Uniprot database (http://www.uniprot.org/uniprot/Q9CWV4), *Mageb16* encodes for a protein of 363 amino acids (41 kDa). The molecular weight of MAGEB16 protein was verified using Western blotting methodology (Fig. 1). According to the Cell-PLoc 2.0 bioinformatics tool for predicting subcellular localization of proteins in different organisms^9^, MAGEB16 is predicted to be located in the cytosol. We confirmed this prediction using confocal imaging (Fig. 1), wherein, MAGEB16 has been detected in cell surface and/or cytosol.

To identify the mechanism by which MAGEB16 regulates the onset of differentiation in ESCs, we generated KD ESCs in which *Mageb16* is silenced. Applying the classical hanging drop protocol, we differentiated the cells until day 22 and performed microarray studies at different time points of differentiation. In summary, DAVID analysis of our data showed that the level of key pluripotency markers such as *Pou5f1, Nanog* and *Sox2* declined faster during differentiated KD ESCs as compared to both SCR and WT ESCs (Figure S1). Based on the GOs and KEGG pathways enriched in *Mageb16* deficient differentiated KD ESCs, *Mageb16* mainly regulated the development of mesodermal directed lineages such as blood-, blood vessel-, heart-, muscle-, bone-, cartilage-, metanephros-, epithelial- and kidney development. In addition, the enriched KEGG pathways such as ECM-receptor interaction, PPAR signalling pathway (crucial for maintenance of hematopoietic stem cells), linoleic metabolism (associated with mesenchymal stem cells), hypertrophic and dilated cardiomyopathy suggest mesoderm related lineage specification too. However, enrichment of some ectoderm associated biological processes such as neurogenesis, lung development and axonogenesis suggest regulation of ectoderm and epithelial lineages also. Notably, the time kinetic of expression of classic contractility genes (Figure S2) was very similar in differentiating ESCs and SCR. However, the expression values were higher in differentiating KD ESCs which could be correlated to a higher beating activity in 13-days differentiating KD ESCs. These findings suggest a stronger orientation towards cardiomyogenic differentiation in differentiating *Mageb16* KD ESCs.

Notably, analysis of microarray data indicated a faster downregulation of pluripotency genes such as *Pou5f1*, *Nanog* and *Sox2* as compared to SCR ESCs and ESCs (Cluster 7, Figure S1). However, the expression of Pou5f1, Nanog and Sox2 did not completely downregulated in 12-, 15-, and 22-days ESC- and SCR EBs. These results suggest that these factors regulate self-renewal but they also are involved in the differentiation processes of ESCs. In this context, it has been shown that Pou5f1, Nanog and Sox2 not only play an important role in maintaining pluripotency but are also involved in differentiation depending on the presence and expression level of other differentiation factors^10,11^. As demonstrated earlier, a certain amount of Pou5f1 also induces differentiation of ESCs to mesodermal and primitive endoderm cells^10^.

Interestingly, while the expression level of pluripotency factors was very similar in undifferentiated KD ESCs and SCR ESCs, significant differences were observed in 12, 15 and 22-day EBs (Fig. 3, cluster 7, Figure S1). Although *Mageb16* expression level was very low in KD ESCs, the expression of pluripotency genes identified in Cluster 5 (including *Pou5f1*, *Nanog* and *Sox2*), remained very high and similar to that of undifferentiated SCR ESCs. From these findings, we may conclude that *Mageb16* is not directly involved in regulating expression of pluripotency genes but rather acting through fine-tuning the differentiation program of ESCs when differentiation is initiating.

Interestingly, the expression of genes such as *Hat1*, *Lin28a* and *Brca1* participating in the epigenetic regulation of gene expression, as well as in nucleocytoplasmic transport and ncRNA processing indicated the characteristic cluster 4 expression pattern with significant lower expression levels in 12, 15 and 22-day KD EBs in comparison to the corresponding SCR-or the WT EBs (Figure S1). HAT1 plays an important epigenetic role in cellular chromatin assembly^12^. Epigenetic DNA modifications including chromatin rearrangements by histone modifications are involved in mammalian developmental processes and are characteristic of differentiation of ESCs toward somatic cells^13^. *Hat1* has been found to be overexpressed in adult stem cells such as hematopoietic stem cells (HSCs)^14^ and neural stem cells^15^.

LIN28 is an RNA-binding protein promoting pluripotency of ESCs via regulation of the microRNA let-7 regulates transition between pluripotency and committed cell lineages^16^. LIN28 is highly upregulated in undifferentiated cells and its expression declined during differentiation^16^. Our findings also demonstrated high expression levels in SCR ESCs and KD ESCs and the expression declined with increasing time of differentiation with significantly lower levels at 12- to 22-day in KD EBs as compared to corresponding SCR EBs.

There is a clear indication that depletion of *Mageb16* is crucial for differentiation of ESCs specifically towards mesoderm depended adipogenesis, osteogenesis, cardiovascular lineages development, limb morphogenesis along with epithelial differentiation and neurogenesis (Cluster 1 and 4). These crucial developmental processes are well orchestrated by the participation of the highly conserved *Hox* genes (*Hoxd9, Hoxc8 and Hoxa9*). In this context, *Hox* genes were found in Cluster 1 and 4 showing a high expression level in 12- to 22-day *Mageb16* deficient KD EBs in comparison to the corresponding *Mageb16* expressing SCR EBs. Additionally, *Tbx4* (cluster 4) and *Itga4* which regulate osteoblast differentiation^17^ as well as *Shh* and *Igfbp5* (cluster 1) which are participating on the development of cardiovascular system^18^ are also highly upregulated in 12- to 22-day *Mageb16*-deficient KD ESCs in comparison to the corresponding control EBs. In addition, mesodermal genes were significantly faster downregulated in the differentiated *Mageb16* deficient KD ESCs compared to the SCR ESCs (Cluster 7, Table 1) correlating with the prominent formation of mesodermal derivatives.

Interestingly we found in Cluster 5, genes that encode for nuclear pore proteins which are essential for exchanging mRNAs and ncRNA between nucleus and cytosol. The expression level of these genes becomes lower during differentiation of *Mageb16* deficient KD ESCs in comparison to *Mageb16* expressing differentiated SCR ESCs and ESCs. There is increasing evidence that the process of nucleocytoplasmic trafficking (NCT), including nuclear pore complex proteins regulate ESCs differentiation^19–21^. Among those genes showing low expression values with progressive differentiation of *Mageb16* KD ESCs, *Ranbp17*, *Nup43*, *Nup133* and *Nup37* have been identified. *Xpo4, Xpo5* and *Xpo7*, which belong to the exportin gene family mediating the transport of molecules from nucleus to cytosol, also exhibited low expression levels. Interestingly, importins such as *Ipo5* and *Ipo11* and exportins such as *Xpo4, Xpo5* and *Xpo7* are also critical for the regulation of differentiation in ESCs^21–23^. Normally, their expression is tightly regulated during lineage commitment of ESCs^21–23^. Moreover, the expression levels of *Kpnb1*, *Kpna1*, *Tnpo2* and *Tnpo3* which are crucial for nuclear protein export^21–23^ were lower in *Mageb16* deficient differentiated KD ESCs. In conclusion, the rapid downregulation of importins and exportins during differentiation of *Mageb16* deficient KD ESCs correlates well with faster progression of differentiation. In this context, the opposite phenomenon has been observed by *Strip2* deficient KD ESCs. In this context, we recently demonstrated for the first time that *Strip2* is essential for the onset of the differentiation in ESCs^24,25^.

Until now, the functional role of *Mageb16* is unknown. More recently it has been demonstrated that among different tissues, MAGEB16 was highly upregulated only in human and mice testis^6^. Furthermore, authors reported that the CpG islands of the 5′ upstream region of MAGEB16 was highly demethylated in the testis in striking contrast to highly methylated MAGEB16 expressed in other tissues^6^. The authors have also suggested that MAGEB16 is a critical regulator of spermatogenesis. This report^6^ strongly supports our findings, demonstrating that genes involved in the process of meiosis and spermatogenesis (*Dnmt3a, Rec8, Tex15, Sycp3, Tcfl5, Hsf2, Nr6a1, Mael, Rad18, Nlrp14*) were downregulated at later time points of differentiation in the *Mageb16* KD ESCs in comparison to *Mageb16* expressing SCR ESCs (Cluster 3 genes, Table 1 and Table S1). Accordingly, among the spermatogenesis genes, DNMT3A was reported to induce epigenetic modification of DNA via CpG island methylation, which again suggests the important role of MAGEB16 in spermatogenesis. One of the key regulators of spermatogenesis is *Mael* (Supplementary Information, Figure S1) which is expressed in male germ cells and testis but under pathological conditions also in cancer cells^26^. Demethylation of CpG islands in *Mael* is also associated with its expression^26^. Moreover, in the absence of MAGEB16, spermatogenesis genes become downregulated during formation of spermatocytes. From these findings, we may suggest that *Mageb16* is a master regulator of spermatogenesis. Our results additionally demonstrate that MAGEB16 is a key regulator of differentiation processes in ESCs via regulation of the expression of pluripotency factors. In the absence of *Mageb16*, expression level of pluripotency factors declined more rapidly during differentiation of ESCs and their expression level remained very low as compared to MAGEB16 expressing ESCs. Our findings give strong evidences that *Mageb16* could be critical for the expression of genes participating in nucleocytoplasmic trafficking of proteins and RNAs between cytosol and nucleus (and vice versa), which in turn may regulate the expression of pluripotency factors such as *Pou5f1* and *Nanog*. However, the cellular mechanisms by which *Mageb16* affect the nucleocytoplasmic trafficking of factors essential for differentiation of ESCs remain to be elucidated.

## Methods

### Murine ESC culture and *Mageb16* KD stable cell line generation

Permanent knockdown of *Mageb16* has been performed using RNA interference technology that employs a short hairpin RNA targeting *Mageb16*. Culturing of the murine CGR8 ESCs (ECACC 95011018) was performed as reported previously^7^. Four sets of shRNA Plasmid vector (pGFP-V-RS_HuSH ^TM^ Origene) targeting *Mageb16* are used. These plasmids are retroviral silencing plasmids of Origene (Rockville, MD, USA) with GFP plus screening for transfection efficiency and puromycin resistance for stable polyclonal cell line generation. The shMageb16 target sequence on its mRNA was 5′-CATTCCACCAGATCAGTCAGATAGCACAG-3′ from the exon region (ENSMUSE00000697770) and the sequence is common to both *Mageb16* transcript variants 1 and 2. Non-targeting scrambled shRNA plasmid (TR30013) was also purchased from Origene. Transfection complex of Lipofectamine® LTX with Plus™ reagent (Life Technologies Cn: 15338–100) and shRNA plasmid targeting *Mageb16* was prepared and transfected into CGR8 as per the protocol suggested by the producer. A similar transfection complex with shRNA plasmid of non-target was prepared and transfected into CGR8 for Scrambled. The selection process was initiated immediately after 24 hours of transfection with 3.5 µg/ml puromycin to have stable knockdown clones. After 12 days of transfection, the surviving and stably proliferating cells were selected for further investigations.

### Differentiation of ESCs using hanging drop protocol

Random differentiation of *Mageb16* KD ESCs (KD ESCs), scrambled ESCS (SCR ESCs) and wild type ESCs (WT ESCs) was performed using hanging drop method for forming embryoid bodies (EBs) as described previously^7^. EBs from all three-cell populations were collected at different time intervals- 2-, 4-, 12, 15- and day 22 for microarray studies.

### RNA isolation and microarray dataset generation

Total RNA was isolated from ESCs and EBs generated with KD-, SCR- and WT ESCs using RNeasy mini kit (Qiagen, Hilden, Germany). Then, 100 ng total RNA was used for amplified RNA (aRNA) amplification with GeneChip 3′ IVT Express Kit (Affymetrix, Santa Clara, CA, USA). After 16 h of biotinylated *in vitro* transcription, aRNA was purified and 15 *μ*g of purified aRNA was fragmented with fragmentation buffer. Next, 12.5 *μ*g of fragmented aRNA was hybridized with Mouse Genome 430 2.0 arrays (Affymetrix) for 16 h at 45 °C. Arrays were washed and stained with phycoerythrin with Affymetrix Fluidics Station 450 and scanned using Affymetrix Gene-Chip Scanner 3000 7 G (Affymetrix).

### Transcriptome analysis and Gene Ontology (GO) enrichment Analysis

The quality control matrices were confirmed with Affymetrix GCOS software. The raw data were background corrected, summarized and normalized using RMA algorithm executed by R bioconductor packages^27^. A PCA was performed to observe the transcriptome variability between the different time points of differentiation. Significantly regulated transcripts were determined by empirical Bayes linear model applied using the LIMMA package in R^28^. The significance of the change was calculated correcting *P-*value of *t*-score with false discovery rate using Benjamini–Hochberg method at *P* < 0.05 and a 2-fold size of change was calculated. Furthermore significantly differential expressed probe sets were analysed by *k*-mean cluster analysis after a transcript-wise normalization of signal values to a mean of 0 and standard deviation (SD) of 1 using Euclidian distance measurement and *k* = 7, using the Cluster 3.0 tool. To determine the biological significance of differentially expressed transcripts (DETs), the differential expressed genes were analysed by the Database for Annotation, Visualization and Integrated Discovery (DAVID) bioinformatics tool (http://david.abcc.ncifcrf.gov/). DAVID provides biological processes (BPs), molecular function (MF) and cellular component (CC) for the differential expressed transcripts (DET) with an EASE enrichment score at *P* < 0.01. For filtering the highly significant enriched Biological Processes and KEGG pathways, adjusted P value with Benjamini correction was used with the threshold p value < 0.05.

### Validation of microarray data sets with qRT-PCR

ABI 7500 FAST Detection System (Applied Biosystems) was used to read the gene profile generated at quantitative real-time-polymerase chain reaction. Total RNA from KD- SCR- and WT ESCs and from their EBs at day 2-, 4-, 12-, 15- and 22-day old EBs was reverse transcribed using SuperScript VILO cDNA synthesis kit with random hexamers as suggested (Invitrogen GmbH). qRT-PCR was performed in triplicates for every sample using QuantiFast SYBER Green PCR assay (Qiagen) and TaqMan Gene Expression Assay (Life Technologies). QuantiFast SYBER Green PCR assay reactions were performed with the following conditions, beginning with Taq activation at 95 °C for 5 min, thereafter 40 cycles of 10 sec denaturation at 95 °C, 30 sec of combined annealing and extension at 60 °C and finally ending with a melting curve acquisition. Primers used are shown in Table S2.

### Western blotting and immunohistochemistry analysis

Western blot analysis was performed with 10 µg total protein. Total protein extracts were separated using 4–12% Bis-Tris Plus precast polyacrylamide gels (Thermo Fisher, Karlsruhe, Germany) by electrophoresis and blotted onto polyvinylidene fluoride membranes (Thermo Fisher, Karlsruhe, Germany). After blocking the membranes with 5% non-fat milk suspended in T-PBS (0.1% Tween 20, Sigma–Aldrich), the membranes were incubated with primary antibodies in 1% non-fat milk at 4 °C overnight. The proteins were visualized using the ECL Pierce Fast Western Blot system (Thermo Fisher Scientific, 35050).

Polyclonal antibodies against MAGEB16 were generated by Pineda Antibody services by immunization of rabbits with the peptide Mageb16-3: NH2-CQGSPVIPPDQSDSTDL-CONH2 (17 amino acids). Anti-GAPDH (Abcam, ab 9485) was purchased from Abcam (Abcam, Cambridge, UK). For immunocytochemistry analysis, undifferentiated KD and SCR ESCs were plated on gelatine-coated coverslips for 2 days. On day 2, the ESCs were fixed with 99% methanol (Roth, Germany) at −20 °C for 10 min. Thereafter cells were permeabilized with 0.3% triton X-100 (Sigma-Aldrich, Germany) at room temperature (RT) for 20 min. Cells were blocked with 5% bovine serum albumin (BSA) (PAA, Austria) and stained with anti-MAGEB16 (1:50) and anti SSEA-1 (Santa cruz, sc-101462, 1:50). Primary antibodies were detected with species matched Alexa-488 conjugated secondary antibodies (Invitrogen, USA). Alexa-568 conjugated secondary antibodies and DAPI (Invitrogen, USA) were used to stain cell nuclei as recommended by the manufacturer’s protocol. Images were captured with an Axiovert 200 microscope and Axiovision 4.3 software (Carl Zeiss, Germany).

We further visualized the expression of MAGEB16 using confocal microscopy. For this aim immunocytochemistry was performed with 4% PFA fixed undifferentiated SCR ESCs on day2. To visualize MAGEB16, staining was performed with and without permeabilization. Briefly, ESCs were fixed with 4% PFA (Roth, Germany) at room temperature for 10 min. For permeabilization, cells were permeabilized with 0.3% triton X-100 (Sigma-Aldrich, Germany) at room temperature (RT) for 20 min and some cells were preceded without permeabilization to next step (non permeabilized). The cells were then blocked with 5% bovine serum albumin (BSA) (PAA, Austria) and stained with anti-MAGEB16 (1:50) and anti OCT4 (Santa cruz, sc-5279, 1:250). Primary antibodies were detected with species matched Alexa-488 conjugated secondary antibodies (Invitrogen, USA). Alexa-568 conjugated secondary antibodies and Hoechst (Invitrogen, USA) were used to stain cell nuclei as recommended by the manufacturer’s protocol. Images were captured with an Olympus FluoView1000 confocal system (Olympus).

### Data availability statement

The microarray data were submitted to the GEO database with the association number for the full mRNA expression dataset GSE103615.

## Electronic supplementary material

Supplementary figures and tables

13 days SCR EBs

13 days SCR EBs
