# Supplementary material for: Depletion of Mageb16 induces differentiation of pluripotent stem cells predominantly into mesodermal derivatives
Source: Sci Rep. 2017 Oct 27;7:14285. doi: 10.1038/s41598-017-14561-z (PMC5660239; doi:10.1038/s41598-017-14561-z)
Supplement: Supplementary file 1 — Supplementary figures and tables [file 41598_2017_14561_MOESM1_ESM.pdf]

John Antonydas Gaspar[1§], Sureshkumar Perumal Srinivasan[1§], Poornima Sureshkumar[1], Michael Xavier Doss[1], Jürgen Hescheler[1], Symeon Papadopoulos[2], Agapios Sachinidis[1*]

Title: Depletion of Mageb16 induces differentiation of pluripotent stem cells predominantly into mesodermal derivatives

[1]University of Cologne (UKK), Institute of Neurophysiology and Center for Molecular Medicine Cologne (CMMC), Robert-Koch-Str. 39, 50931, Cologne, Germany

[2]University of Cologne, Center of Physiology and Pathophysiology, Institute of Vegetative Physiology, Robert-Koch-Str. 39, 50931 Cologne, Germany

[§]John Antonydas Gaspar and Sureshkumar Perumal Srinivasan: Equally contributed

Corresponding author:
Prof. Dr. Agapios Sachinidis
University of Cologne (UKK),

Institute of Neurophysiology and Center for Molecular Medicine Cologne (CMMC)

Robert-Koch-Str. 39

50931, Cologne, Germany

Tel: +49 (0) 221 478 7373

Fax: +49 (0) 221 478 6965

Email: a.sachinidis@uni-koeln.de

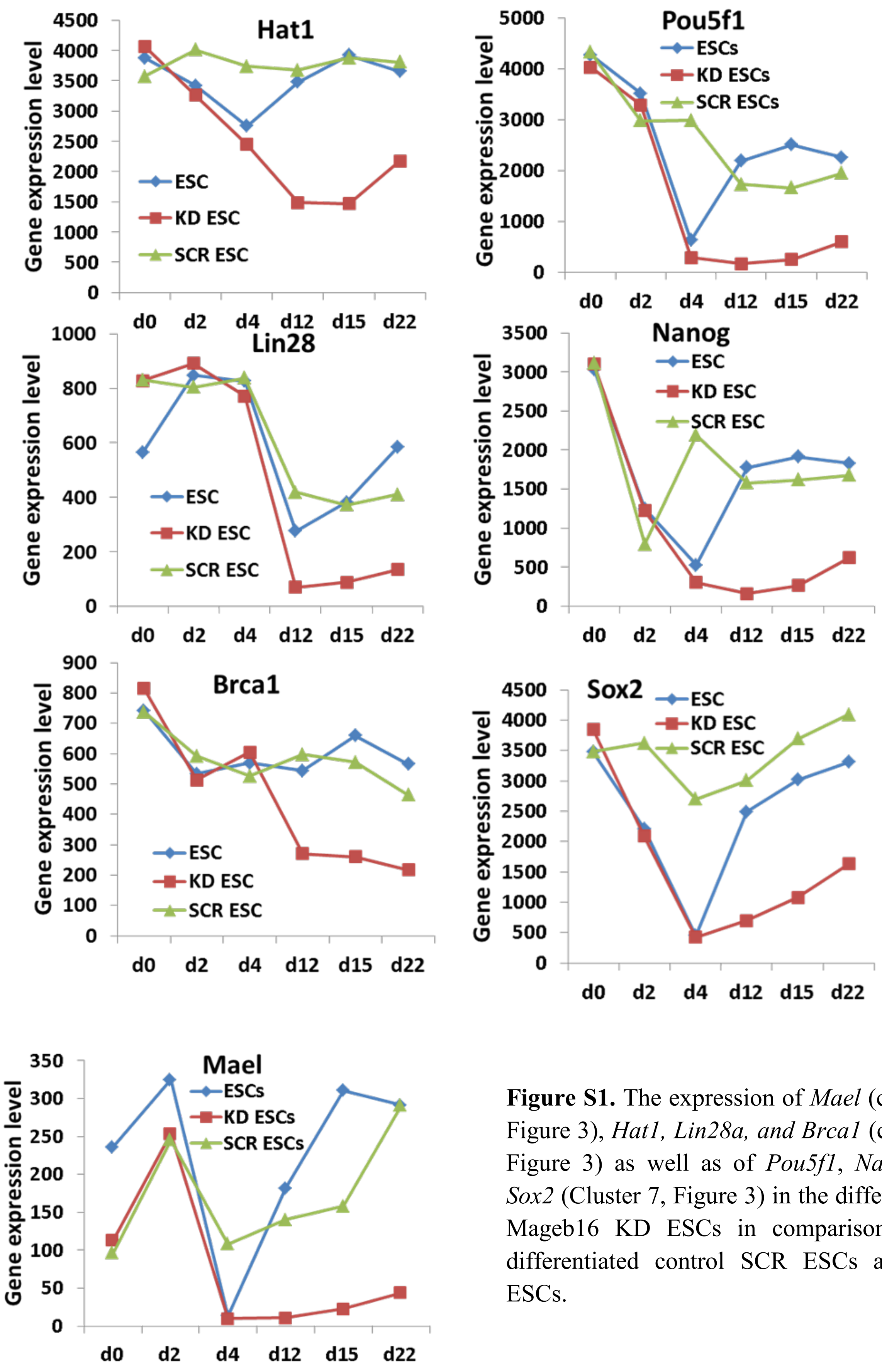

Figure S1. The expression of *Mael* (cluster 3, Figure 3), *Hat1, Lin28a, and Brca1* (cluster 5, Figure 3) as well as of *Pou5f1*, *Nanog* and *Sox2* (Cluster 7, Figure 3) in the differentiated Mageb16 KD ESCs in comparison to the differentiated control SCR ESCs and wild ESCs.

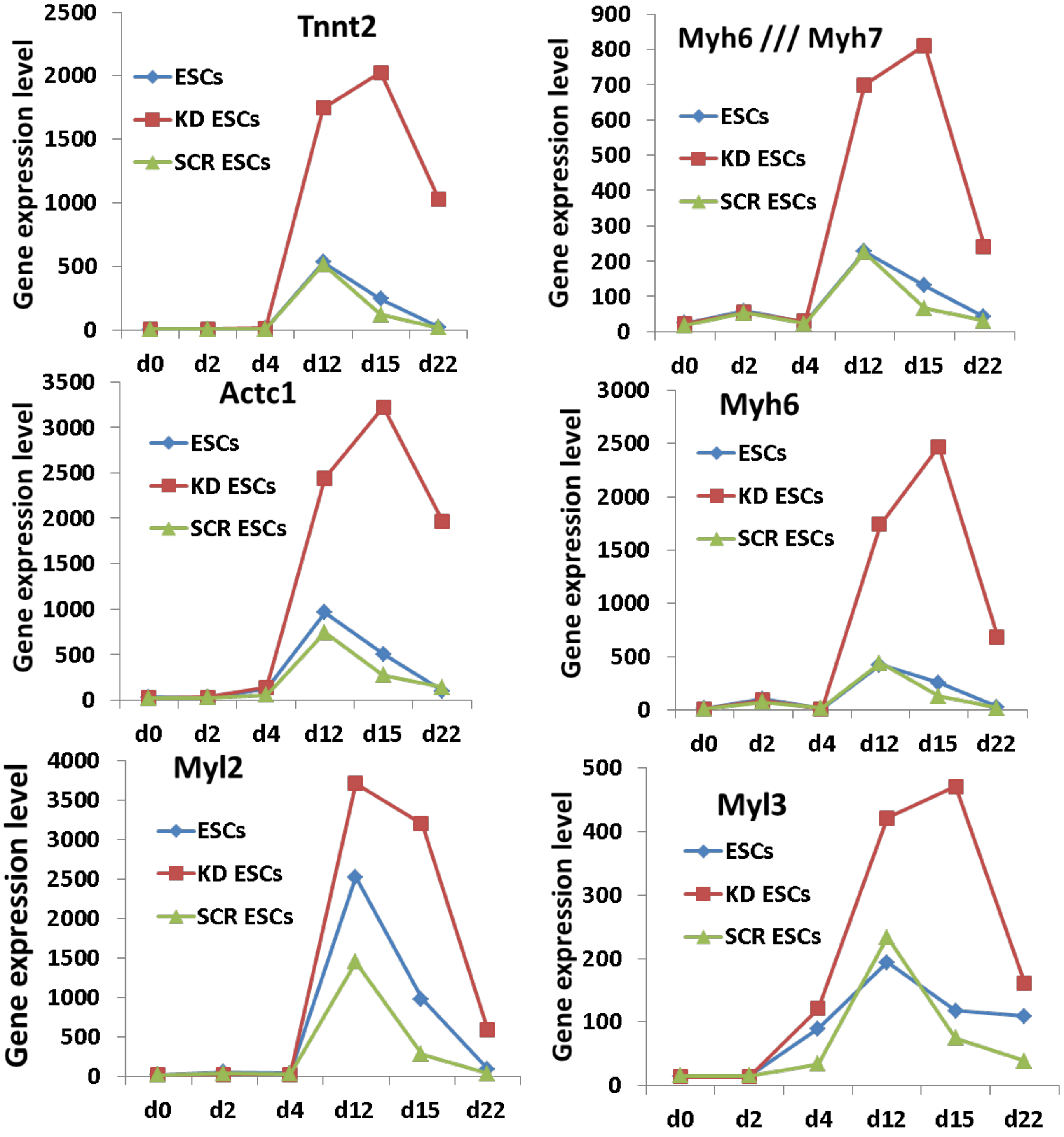

Figure S2. The expression of the cardiomyocytes specific contractile genes, *Tnnt2*, *Actc1*, *Myl2*, *Myl3* (cluster 1, Figure 3), *Myh6* and *Myh7* (cluster 6, Figure 3) in the differentiated Mageb16 KD ESCs in comparison to the differentiated control SCR ESCs and wild ESCs.

Table S1. Complete GO and KEGG pathways of the cluster specific genes indicated in Figure 3				
**Cluster 1**				
Category	Term	Count	PValue	Genes
**GOTERM_BP_5**	**GO:0009888~tissue development**	**49**	**7.76E-12**	**MEF2C, ENPP1, MYL2, PTGS2, FGF9, NPNT, ONECUT2, PRRX1, ZEB2, POSTN, OTOR, TIMP3, SHH, EDNRA, APP, HOXA3, HOXA5, ALDH1A3, BCL2, CHST11, SEMA3C, PTN, ANGPT1, SEMA3A, RARB, NR2F2, GPNMB, RUNX2, PRKCA, EGFR, ACTC1, DMP1, FZD1, IGF1, MECOM, COL5A2, KITL, FOXP2, SHOX2, TNNT2, HOXB3, HOXB4, CYP7B1, RDH10, HOXB5, JUN, PLN, ZFPM2, COL1A1**
GOTERM_BP_5	GO:0010810~regulation of cell-substrate adhesion	12	2.69E-09	A930038C07RIK, NPNT, BCL2, ONECUT2, DMP1, CCDC80, NID1, COL1A1, THBS1, COL8A1, PIK3R1, EMILIN1
**GOTERM_MF_5**	**GO:0005509~calcium ion binding**	**53**	**4.26E-09**	**PDP1, ARSB, CLSTN2, MASP1, LTBP3, NPNT, FAM20C, ANO1, FSTL1, DLK1, EFHD2, GSN, SLC24A3, ANO4, PLCB1, PRKCA, AMY1, PCDHB7, SPARCL1, PCDH11X, MMP12, SLIT2, PLCE1, VSNL1, RELN, MFAP4, SRI, MYL4, FKBP7, MYL2, CD248, GALNT4, PCDHB11, FAT4, PCDHB19, PCDHB16, TTYH2, PCDHB17, MAN1A, TGM2, EFCAB1, THBS1, SLC8A1, CUBN, TRPC6, ITGA1, ANXA1, NID1, SPARC, PCDHB20, PCDH17, ANXA2, CACNA1D**
GOTERM_MF_5	GO:0008201~heparin binding	15	2.98E-08	PTPRC, FGF9, CCDC80, COL25A1, FSTL1, PF4, POSTN, NCAM1, A930038C07RIK, APP, CRISPLD2, PTN, ADAMTS1, THBS1, GPNMB
**GOTERM_BP_5**	**GO:0001568~blood vessel development**	**24**	**5.57E-08**	**MEF2C, EMCN, FGF9, SOCS3, LEPR, IL18, TGFBR2, COL3A1, PRRX1, ELK3, MEIS1, CXCL12, VASH1, SHH, ANXA2, EDNRA, HOXA3, TGM2, RHOB, SEMA3C, ANGPT1, ZFPM2, COL1A1, NR2F2**
**GOTERM_BP_5**	**GO:0001944~vasculature development**	**24**	**8.69E-08**	**MEF2C, EMCN, FGF9, SOCS3, LEPR, IL18, TGFBR2, COL3A1, PRRX1, ELK3, MEIS1, CXCL12, VASH1, SHH, ANXA2, EDNRA, HOXA3, TGM2, RHOB, SEMA3C, ANGPT1, ZFPM2, COL1A1, NR2F2**
**GOTERM_BP_5**	**GO:0048514~blood vessel morphogenesis**	**21**	**1.36E-07**	**MEF2C, EMCN, FGF9, LEPR, IL18, TGFBR2, PRRX1, ELK3, MEIS1, CXCL12, VASH1, SHH, ANXA2, EDNRA, HOXA3, TGM2, RHOB, SEMA3C, ANGPT1, ZFPM2, NR2F2**

**GOTERM_BP_5**	**GO:0048706~embryonic skeletal system development**	**14**	**1.43E-07**	**NDST1, FGF9, TGFBR2, PRRX1, DLK1, SHH, HOXB3, SHOX2, HOXB4, RDH10, HOXA3, HOXA5, HOXB5, CHST11**
**GOTERM_BP_5**	**GO:0048705~skeletal system morphogenesis**	**16**	**9.57E-07**	**MEF2C, NDST1, TGFBR2, PRRX1, OTOR, SHOX2, HOXB3, HOXB4, RDH10, HOXA3, HOXA5, HOXB5, CHST11, COL1A1, RUNX2, IDUA**
GOTERM_BP_5	GO:0045785~positive regulation of cell adhesion	10	9.88E-07	A930038C07RIK, VAV3, NPNT, DMP1, TGM2, CCDC80, NID1, THBS1, COL8A1, EMILIN1
**GOTERM_BP_5**	**GO:0060348~bone development**	**15**	**1.58E-06**	**MEF2C, PTGS2, FGF9, DMP1, IGF1, SHH, SHOX2, HOXB4, CHRDL1, BCL2, PTN, COL1A1, GPNMB, RUNX2, IGFBP5**
**GOTERM_BP_5**	**GO:0009887~organ morphogenesis**	**36**	**2.59E-06**	**MEF2C, TSHZ1, NDST1, SOX1, FGF9, NPNT, PRRX1, ZEB2, OTOR, MEIS1, SHH, HOXA3, HOXA5, BCL2, ALDH1A3, CHST11, SEMA3C, SEMA3A, RARB, COL8A1, RUNX2, IDUA, EGFR, ACTC1, MAFB, TGFBR2, IGF1, HOXB3, TNNT2, SHOX2, HOXB4, CYP7B1, RDH10, HOXB5, ADAMTS1, COL1A1**
**GOTERM_BP_5**	**GO:0048562~embryonic organ morphogenesis**	**17**	**3.09E-06**	**TSHZ1, NDST1, MAFB, FGF9, TGFBR2, PRRX1, SHH, HOXB3, SHOX2, HOXB4, RDH10, HOXA3, HOXA5, ALDH1A3, HOXB5, CHST11, RARB**
**GOTERM_BP_5**	**GO:0048568~embryonic organ development**	**21**	**3.10E-06**	**EGFR, TSHZ1, NDST1, MAFB, FGF9, SOCS3, TGFBR2, PRRX1, SHH, HOXB3, VCAM1, SHOX2, HOXB4, RDH10, HOXA3, HOXA5, HOXB5, ALDH1A3, CHST11, ZFPM2, RARB**
GOTERM_BP_5	GO:0010811~positive regulation of cell-substrate adhesion	8	3.53E-06	A930038C07RIK, NPNT, DMP1, CCDC80, NID1, THBS1, COL8A1, EMILIN1
**GOTERM_BP_5**	**GO:0051216~cartilage development**	**12**	**3.86E-06**	**MEF2C, SHOX2, HOXB3, PRKCA, HOXA3, HOXA5, FGF9, CHST11, PRRX1, COL1A1, OTOR, RUNX2**
**GOTERM_BP_5**	**GO:0007420~brain development**	**23**	**3.90E-06**	**EGFR, EGR2, NDST1, SOX1, MAFB, PLXNA2, TGFBR2, ZEB2, MECOM, PRKG1, CXCL12, SHH, FOXP2, APP, ALDH1A3, BCL2, RELN, ROBO2, ID4, SEPP1, RARB, NR2F2, NFIB**
**GOTERM_BP_5**	**GO:0048704~embryonic skeletal system morphogenesis**	**11**	**4.14E-06**	**SHOX2, HOXB3, HOXB4, RDH10, HOXA3, NDST1, HOXA5, HOXB5, CHST11, TGFBR2, PRRX1**
GOTERM_BP_5	GO:0031325~positive regulation of cellular metabolic process	37	6.13E-06	MEF2C, MEF2A, ONECUT2, NFIX, PRDM16, ZXDA, SHH, APP, BCL2, BCL11B, RARB, RUNX2, ADAM9, PRKCA, EGR1, MAF, PTPRC, H60A, EGR2, IKZF2, MAFB, ESRRG, IGF1, MECOM, SAMD4, KITL, PPARGC1A, FCGR3, SHOX2, RNF180, ATXN1, JUN, EBF1, HIVEP3, ZFPM2, NFIA,

				NFIB
GOTERM_BP_5	GO:0010604~positive regulation of macromolecule metabolic process	36	1.05E-05	MEF2C, MEF2A, ONECUT2, NFIX, PRDM16, ZXDA, SHH, APP, BCL2, BCL11B, RARB, RUNX2, ADAM9, PRKCA, EGR1, MAF, PTPRC, EGR2, IKZF2, MAFB, ESRRG, IGF1, MECOM, SAMD4, KITL, PPARGC1A, FCGR3, SHOX2, RNF180, ATXN1, JUN, EBF1, HIVEP3, ZFPM2, NFIA, NFIB
GOTERM_BP_5	GO:0016477~cell migration	20	1.09E-05	EGFR, PRKCA, PLAT, VAV3, RHOX5, SOX1, PLXNA2, ITGA1, ZEB2, PF4, PRKG1, CXCL12, KITL, SHH, FCGR3, GAB2, SEMA3C, RELN, NR2F2, PLAU
KEGG_PATHWAY	mmu04512:ECM-receptor interaction	12	1.60E-05	CD47, CD36, NPNT, COL3A1, COL6A2, COL1A2, ITGA1, COL6A1, RELN, COL1A1, THBS1, COL5A2
GOTERM_CC_5	GO:0042383~sarcolemma	9	2.00E-05	KCND3, SLC8A1, BGN, COL6A3, COL6A2, CAMK2D, ANXA1, COL6A1, ANXA2
**GOTERM_BP_5**	**GO:0001649~osteoblast differentiation**	**9**	**2.02E-05**	**MEF2C, SHOX2, FGF9, IGF1, COL1A1, GPNMB, RUNX2, SHH, IGFBP5**
**GOTERM_BP_5**	**GO:0007417~central nervous system development**	**25**	**2.20E-05**	**SOX1, NDST1, PLXNA2, ZEB2, PRKG1, CXCL12, SHH, APP, BCL2, ALDH1A3, BCL11B, ROBO2, RARB, SEPP1, NR2F2, EGFR, EGR2, MAFB, ALDH5A1, TGFBR2, MECOM, FOXP2, ID4, RELN, NFIB**
KEGG_PATHWAY	mmu04510:Focal adhesion	18	3.11E-05	EGFR, PRKCA, VAV3, MYL2, COL3A1, ITGA1, IGF1, COL5A2, BCL2, JUN, COL1A2, COL6A2, COL6A1, RELN, COL1A1, THBS1, PIK3R1, PARVA
GOTERM_BP_5	GO:0001525~angiogenesis	14	3.12E-05	EMCN, FGF9, LEPR, IL18, TGFBR2, ELK3, CXCL12, MEIS1, VASH1, SHH, ANXA2, EDNRA, RHOB, ANGPT1
GOTERM_BP_5	GO:0009966~regulation of signal transduction	35	6.09E-05	FGF9, NPNT, ONECUT2, PRRX1, ZEB2, ITPKB, PRDM16, TIMP2, D0H4S114, APP, HTRA1, ALDH1A3, CHST11, TGM2, THBS1, RUNX2, AFAP1, PRKCA, PTPRC, VAV3, SOCS3, PSD3, IGF1, GRIA3, RGS16, KITL, DKK2, NCAM1, ATXN1, SHOX2, CYP7B1, PLCE1, HIPK3, RELN, KALRN
GOTERM_BP_5	GO:0031328~positive regulation of cellular biosynthetic process	31	6.31E-05	MEF2C, MEF2A, ONECUT2, NFIX, PRDM16, ZXDA, SHH, APP, BCL11B, RARB, RUNX2, EGR1, MAF, H60A, IKZF2, EGR2, MAFB, ESRRG, IGF1, MECOM, SAMD4, PPARGC1A, FCGR3, ATXN1, SHOX2, JUN, EBF1, HIVEP3, ZFPM2, NFIA, NFIB
GOTERM_BP_5	GO:0051173~positive regulation of nitrogen compound metabolic process	30	6.63E-05	MEF2C, MEF2A, ONECUT2, NFIX, PRDM16, ZXDA, SHH, APP, BCL11B, RARB, RUNX2, EGR1, MAF, PTPRC, H60A, IKZF2, EGR2, MAFB, ESRRG, IGF1, MECOM, PPARGC1A, ATXN1, SHOX2, JUN, EBF1, HIVEP3, ZFPM2, NFIA, NFIB

GOTERM_BP_5	GO:0045941~positive regulation of transcription	28	7.13E-05	MEF2C, MEF2A, ONECUT2, NFIX, PRDM16, ZXDA, SHH, APP, BCL11B, RARB, RUNX2, EGR1, MAF, IKZF2, EGR2, MAFB, ESRRG, IGF1, MECOM, PPARGC1A, ATXN1, SHOX2, JUN, EBF1, HIVEP3, ZFPM2, NFIA, NFIB
GOTERM_BP_5	GO:0009891~positive regulation of biosynthetic process	31	7.53E-05	MEF2C, MEF2A, ONECUT2, NFIX, PRDM16, ZXDA, SHH, APP, BCL11B, RARB, RUNX2, EGR1, MAF, H60A, IKZF2, EGR2, MAFB, ESRRG, IGF1, MECOM, SAMD4, PPARGC1A, FCGR3, ATXN1, SHOX2, JUN, EBF1, HIVEP3, ZFPM2, NFIA, NFIB
GOTERM_BP_5	GO:0010557~positive regulation of macromolecule biosynthetic process	30	7.58E-05	MEF2C, MEF2A, ONECUT2, NFIX, PRDM16, ZXDA, SHH, APP, BCL11B, RARB, RUNX2, EGR1, MAF, IKZF2, EGR2, MAFB, ESRRG, IGF1, MECOM, SAMD4, PPARGC1A, FCGR3, ATXN1, SHOX2, JUN, EBF1, HIVEP3, ZFPM2, NFIA, NFIB
GOTERM_BP_5	GO:0030900~forebrain development	15	8.57E-05	EGFR, NDST1, SOX1, ZEB2, PRKG1, MECOM, SHH, FOXP2, APP, ALDH1A3, RELN, ID4, RARB, NR2F2, NFIB
GOTERM_CC_5	GO:0005604~basement membrane	10	8.67E-05	NPNT, CCDC80, NID1, ADAMTS1, SPARC, TIMP2, COL8A1, TIMP3, COL4A5, ANXA2
GOTERM_BP_5	GO:0017015~regulation of transforming growth factor beta receptor signaling pathway	7	8.81E-05	D0H4S114, HTRA1, NPNT, CHST11, ONECUT2, THBS1, PRDM16
GOTERM_BP_5	GO:0045935~positive regulation of nucleobase, nucleoside, nucleotide and nucleic acid metabolic process	29	9.54E-05	MEF2C, MEF2A, ONECUT2, NFIX, PRDM16, ZXDA, SHH, APP, BCL11B, RARB, RUNX2, EGR1, MAF, PTPRC, IKZF2, EGR2, MAFB, ESRRG, IGF1, MECOM, PPARGC1A, ATXN1, SHOX2, JUN, EBF1, HIVEP3, ZFPM2, NFIA, NFIB
GOTERM_BP_5	GO:0010628~positive regulation of gene expression	28	1.12E-04	MEF2C, MEF2A, ONECUT2, NFIX, PRDM16, ZXDA, SHH, APP, BCL11B, RARB, RUNX2, EGR1, MAF, IKZF2, EGR2, MAFB, ESRRG, IGF1, MECOM, PPARGC1A, ATXN1, SHOX2, JUN, EBF1, HIVEP3, ZFPM2, NFIA, NFIB
GOTERM_BP_5	GO:0045597~positive regulation of cell differentiation	15	1.41E-04	PTPRC, SOCS3, TGFBR2, ITPKB, KITL, SHH, NTRK3, CD83, CD36, SERPINF1, BCL2, VNN1, ROBO2, RUNX2, FNDC3B
**GOTERM_BP_5**	**GO:0002062~chondrocyte differentiation**	**6**	**1.45E-04**	**MEF2C, SHOX2, PRKCA, FGF9, CHST11, RUNX2**
**GOTERM_BP_5**	**GO:0060429~epithelium development**	**19**	**1.81E-04**	**EGFR, PTGS2, NPNT, ONECUT2, FZD1, IGF1, ZEB2, MECOM, SHH, CYP7B1, HOXB4, RDH10, HOXA5, HOXB5, JUN, ALDH1A3, BCL2, SEMA3C, SEMA3A**
GOTERM_BP_5	GO:0002053~positive regulation of mesenchymal cell proliferation	6	1.84E-04	SHOX2, FGF9, TGFBR2, PRRX1, SHH, FOXP2

GOTERM_BP_5	GO:0045934~negative regulation of nucleobase, nucleoside, nucleotide and nucleic acid metabolic process	24	1.84E-04	EGR1, EID1, DNMT3A, RHOX5, PRRX1, CBX4, FZD1, ZEB2, SMYD1, PRDM16, SHH, FOXP2, EDNRA, ATXN1, SHOX2, HOXB4, HIPK3, TRPS1, ZFPM2, CUX2, RARB, LCOR, NR2F2, HBA-X
GOTERM_BP_5	GO:0016481~negative regulation of transcription	23	1.93E-04	EGR1, EID1, DNMT3A, RHOX5, PRRX1, CBX4, FZD1, ZEB2, SMYD1, PRDM16, SHH, FOXP2, ATXN1, SHOX2, HOXB4, HIPK3, TRPS1, ZFPM2, CUX2, RARB, LCOR, NR2F2, HBA-X
**KEGG_PATHWAY**	**mmu05414:Dilated cardiomyopathy**	**11**	**2.16E-04**	**TNNT2, SLC8A1, ACTC1, ADCY1, ADRB1, MYL2, PLN, ITGA1, IGF1, CACNA1D, SGCB**
GOTERM_BP_5	GO:0051172~negative regulation of nitrogen compound metabolic process	24	2.17E-04	EGR1, EID1, DNMT3A, RHOX5, PRRX1, CBX4, FZD1, ZEB2, SMYD1, PRDM16, SHH, FOXP2, EDNRA, ATXN1, SHOX2, HOXB4, HIPK3, TRPS1, ZFPM2, CUX2, RARB, LCOR, NR2F2, HBA-X
GOTERM_BP_5	GO:0010464~regulation of mesenchymal cell proliferation	6	2.30E-04	SHOX2, FGF9, TGFBR2, PRRX1, SHH, FOXP2
GOTERM_BP_5	**GO:0060485~mesenchyme development**	8	2.41E-04	EDNRA, RDH10, HOXA5, BCL2, SEMA3C, ZEB2, KITL, SHH
GOTERM_BP_5	GO:0031324~negative regulation of cellular metabolic process	27	2.81E-04	EID1, CBX4, PRRX1, ZEB2, PRDM16, SHH, EDNRA, RARB, NR2F2, HBA-X, EGR1, PRKCA, DNMT3A, PTPRC, RHOX5, FZD1, SMYD1, FOXP2, ATXN1, SHOX2, HOXB4, HIPK3, JUN, TRPS1, ZFPM2, CUX2, LCOR
GOTERM_BP_5	GO:0008284~positive regulation of cell proliferation	19	3.21E-04	EGFR, PTPRC, FGF9, IL18, TGFBR2, PRRX1, IGF1, KITL, DDR2, SHH, FOXP2, SHOX2, CYP7B1, HOXA3, BCL2, TGM2, ID4, CALCRL, RUNX2
GOTERM_BP_5	GO:0030204~chondroitin sulfate metabolic process	5	3.27E-04	CSGALNACT1, A930038C07RIK, BGN, CHST11, DCN
GOTERM_BP_5	GO:0045893~positive regulation of transcription, DNA-dependent	24	3.67E-04	MEF2C, EGR1, MAF, IKZF2, EGR2, MAFB, ONECUT2, ESRRG, IGF1, NFIX, PRDM16, MECOM, PPARGC1A, SHH, ATXN1, SHOX2, APP, JUN, BCL11B, ZFPM2, RARB, RUNX2, NFIA, NFIB
GOTERM_BP_5	GO:0009968~negative regulation of signal transduction	14	4.00E-04	PRKCA, PTPRC, SOCS3, FGF9, ONECUT2, IGF1, RGS16, PRDM16, DKK2, ATXN1, CYP7B1, HTRA1, CHST11, RUNX2
GOTERM_BP_5	GO:0032103~positive regulation of response to external stimulus	7	4.00E-04	NTRK3, PRKCA, CD47, TGM2, THBS1, CXCL12, FCGR3
GOTERM_BP_5	GO:0051254~positive regulation of RNA metabolic process	24	4.06E-04	MEF2C, EGR1, MAF, IKZF2, EGR2, MAFB, ONECUT2, ESRRG, IGF1, NFIX, PRDM16, MECOM, PPARGC1A, SHH, ATXN1, SHOX2, APP, JUN, BCL11B, ZFPM2, RARB, RUNX2, NFIA, NFIB
GOTERM_BP_5	GO:0043009~chordate embryonic development	24	4.31E-04	EGFR, ADAM10, NDST1, FGF9, SOCS3, TGFBR2, PRRX1, ZEB2, DLK1, MECOM, SHH, HOXB3, EDNRA,

				VCAM1, SHOX2, HBA-A1, HOXB4, RDH10, HOXA3, HOXA5, SFRP2, HOXB5, CHST11, ZFPM2
GOTERM_BP_5	GO:0007507~heart development	16	5.39E-04	MEF2C, ACTC1, MYL2, TGFBR2, SMYD1, MECOM, SHH, TNNT2, VCAM1, EDNRA, PLN, SEMA3C, ADAMTS1, ZFPM2, RARB, CALCRL
GOTERM_BP_5	GO:0031327~negative regulation of cellular biosynthetic process	24	5.81E-04	EGR1, EID1, DNMT3A, RHOX5, PRRX1, CBX4, FZD1, ZEB2, SMYD1, PRDM16, SHH, FOXP2, EDNRA, ATXN1, SHOX2, HOXB4, HIPK3, TRPS1, ZFPM2, CUX2, RARB, LCOR, NR2F2, HBA-X
GOTERM_BP_5	GO:0009890~negative regulation of biosynthetic process	24	6.64E-04	EGR1, EID1, DNMT3A, RHOX5, PRRX1, CBX4, FZD1, ZEB2, SMYD1, PRDM16, SHH, FOXP2, EDNRA, ATXN1, SHOX2, HOXB4, HIPK3, TRPS1, ZFPM2, CUX2, RARB, LCOR, NR2F2, HBA-X
GOTERM_BP_5	GO:0010629~negative regulation of gene expression	23	7.34E-04	EGR1, EID1, DNMT3A, RHOX5, PRRX1, CBX4, FZD1, ZEB2, SMYD1, PRDM16, SHH, FOXP2, ATXN1, SHOX2, HOXB4, HIPK3, TRPS1, ZFPM2, CUX2, RARB, LCOR, NR2F2, HBA-X
GOTERM_CC_5	GO:0005833~hemoglobin complex	4	8.08E-04	HBA-A1, HBB-BH1, HBA-X, HBB-Y
GOTERM_BP_5	GO:0045892~negative regulation of transcription, DNA-dependent	19	8.38E-04	EGR1, EID1, DNMT3A, RHOX5, PRRX1, CBX4, PRDM16, SHH, FOXP2, SHOX2, HOXB4, HIPK3, TRPS1, ZFPM2, CUX2, RARB, LCOR, NR2F2, HBA-X
GOTERM_BP_5	GO:0045765~regulation of angiogenesis	7	8.79E-04	SERPINF1, SERPINE1, RHOB, PF4, ADAMTS1, THBS1, VASH1
GOTERM_BP_5	GO:0010648~negative regulation of cell communication	14	8.81E-04	PRKCA, PTPRC, SOCS3, FGF9, ONECUT2, IGF1, RGS16, PRDM16, DKK2, ATXN1, CYP7B1, HTRA1, CHST11, RUNX2
GOTERM_BP_5	GO:0007409~axonogenesis	13	8.82E-04	ALCAM, EPHA7, APP, EGR2, BCL2, BCL11B, RELN, ROBO2, SEMA3A, SLITRK6, CXCL12, SHH, SLIT2
GOTERM_BP_5	GO:0051253~negative regulation of RNA metabolic process	19	9.02E-04	EGR1, EID1, DNMT3A, RHOX5, PRRX1, CBX4, PRDM16, SHH, FOXP2, SHOX2, HOXB4, HIPK3, TRPS1, ZFPM2, CUX2, RARB, LCOR, NR2F2, HBA-X
GOTERM_BP_5	GO:0010558~negative regulation of macromolecule biosynthetic process	23	9.40E-04	EGR1, EID1, DNMT3A, RHOX5, PRRX1, CBX4, FZD1, ZEB2, SMYD1, PRDM16, SHH, FOXP2, ATXN1, SHOX2, HOXB4, HIPK3, TRPS1, ZFPM2, CUX2, RARB, LCOR, NR2F2, HBA-X
GOTERM_BP_5	GO:0010605~negative regulation of macromolecule metabolic process	26	0.001069289	EID1, CBX4, PRRX1, ZEB2, PRDM16, SHH, RARB, NR2F2, HBA-X, EGR1, PRKCA, DNMT3A, PTPRC, RHOX5, FZD1, SMYD1, FOXP2, ATXN1,

				SHOX2, HOXB4, HIPK3, JUN, TRPS1, ZFPM2, CUX2, LCOR
GOTERM_BP_5	GO:0014031~mesenchymal cell development	7	0.001111194	EDNRA, RDH10, BCL2, SEMA3C, ZEB2, KITL, SHH
GOTERM_CC_5	GO:0005581~collagen	5	0.001137847	COL3A1, COL1A2, COL1A1, COL5A2, COL4A5
GOTERM_BP_5	GO:0022008~neurogenesis	27	0.001138853	SOX1, CORIN, ONECUT2, PRDM16, TIMP2, PRKG1, CXCL12, MEIS1, SHH, ALCAM, APP, BCL2, BCL11B, ROBO2, RARB, SEMA3A, NR2F2, NEFM, EGR2, IGF1, SLIT2, NTRK3, EPHA7, SERPINF1, RELN, ID4, SLITRK6
GOTERM_CC_5	GO:0005624~membrane fraction	26	0.001185698	CYP1B1, PTGS2, SGPP1, CYP2J6, ITM2B, SHH, APP, BCL2, ELOVL2, RHOB, PPP1R14C, DNAJC1, GLRB, SLC8A1, PSD3, GRIA3, SAMD4, CAMK2N1, ANXA2, CYP7B1, LAMP2, PLCE1, RDH10, ADRB1, BACE1, MGST1
GOTERM_BP_5	GO:0022612~gland morphogenesis	9	0.001248787	EGFR, CYP7B1, BCL2, TGFBR2, TGM2, SEMA3C, IGF1, SEMA3A, SHH
GOTERM_BP_5	GO:0014033~neural crest cell differentiation	6	0.001321943	EDNRA, RDH10, SEMA3C, ZEB2, KITL, SHH
GOTERM_BP_5	GO:0006029~proteoglycan metabolic process	6	0.001321943	CSGALNACT1, A930038C07RIK, BGN, CHST11, IGF1, DCN
GOTERM_BP_5	GO:0014032~neural crest cell development	6	0.001321943	EDNRA, RDH10, SEMA3C, ZEB2, KITL, SHH
GOTERM_BP_5	GO:0048762~mesenchymal cell differentiation	7	0.001387363	EDNRA, RDH10, BCL2, SEMA3C, ZEB2, KITL, SHH
GOTERM_BP_5	GO:0009967~positive regulation of signal transduction	13	0.001405546	PTPRC, FGF9, NPNT, PRRX1, ZEB2, ITPKB, KITL, NCAM1, SHOX2, ALDH1A3, TGM2, RELN, THBS1
GOTERM_BP_5	GO:0014706~striated muscle tissue development	11	0.001410858	MEF2C, TNNT2, APP, ACTC1, MYL2, PLN, ZFPM2, RARB, NR2F2, SHH, FOXP2
GOTERM_BP_5	GO:0002009~morphogenesis of an epithelium	13	0.001476981	EGFR, HOXB4, CYP7B1, RDH10, HOXA5, NPNT, BCL2, ALDH1A3, IGF1, SEMA3C, ZEB2, SEMA3A, SHH
GOTERM_BP_5	GO:0008016~regulation of heart contraction	7	0.00154367	TNNT2, PRKCA, SLC8A1, AGTR2, ADRB1, PLN, SEMA3A
GOTERM_BP_5	GO:0051174~regulation of phosphorus metabolic process	18	0.001674354	EGFR, PRKCA, PTPRC, VAV3, SOCS3, TGFBR2, ZEB2, PRKCE, KITL, SH3BP5, ATXN1, APP, PLCE1, HIPK3, BCL2, JUN, RELN, PPP1R14C
GOTERM_BP_5	GO:0019220~regulation of phosphate metabolic process	18	0.001674354	EGFR, PRKCA, PTPRC, VAV3, SOCS3, TGFBR2, ZEB2, PRKCE, KITL, SH3BP5, ATXN1, APP, PLCE1, HIPK3, BCL2, JUN, RELN, PPP1R14C
GOTERM_BP_5	GO:0048812~neuron projection morphogenesis	13	0.001709491	ALCAM, EPHA7, APP, EGR2, BCL2, BCL11B, RELN, ROBO2, SEMA3A, SLITRK6, CXCL12, SHH, SLIT2
GOTERM_BP_5	GO:0006935~chemotaxis	10	0.001741708	PRKCA, C3AR1, CCL3, ITGA1, PF4, ROBO2, SEMA3A, CXCL12, SLIT2, FCGR3
GOTERM_BP_5	GO:0048858~cell projection morphogenesis	14	0.001862689	EGR2, ONECUT2, CXCL12, SLIT2, SHH, ALCAM, APP, EPHA7, BCL11B,

				BCL2, RELN, ROBO2, SEMA3A, SLITRK6
GOTERM_BP_5	GO:0060439~trachea morphogenesis	3	0.001876363	HOXA5, TGFBR2, SHH
GOTERM_BP_5	GO:0030324~lung development	10	0.001975904	RDH10, LIPA, HOXA5, CRISPLD2, FGF9, TGFBR2, ZFPM2, SHH, FOXP2, NFIB
GOTERM_BP_5	GO:0032268~regulation of cellular protein metabolic process	17	0.002018811	PRKCA, EGFR, PTPRC, SOCS3, ZEB2, PRKCE, KITL, SAMD4, SHH, RNF180, APP, HIPK3, KRT7, BCL2, JUN, DNAJC1, ADAM9
GOTERM_BP_5	GO:0016525~negative regulation of angiogenesis	5	0.002038418	SERPINF1, PF4, ADAMTS1, THBS1, VASH1
GOTERM_BP_5	GO:0030323~respiratory tube development	10	0.002234593	RDH10, LIPA, HOXA5, CRISPLD2, FGF9, TGFBR2, ZFPM2, SHH, FOXP2, NFIB
GOTERM_BP_5	GO:0030335~positive regulation of cell migration	6	0.00223607	PTP4A1, BCL2, ONECUT2, THBS1, CXCL12, PIK3R1
GOTERM_BP_5	GO:0030334~regulation of cell migration	9	0.002241767	PTP4A1, BCL2, ONECUT2, ABHD2, SEMA3A, THBS1, CXCL12, PIK3R1, SHH
GOTERM_BP_5	GO:0048667~cell morphogenesis involved in neuron differentiation	13	0.002265118	ALCAM, EPHA7, APP, EGR2, BCL2, BCL11B, RELN, ROBO2, SEMA3A, SLITRK6, CXCL12, SHH, SLIT2
GOTERM_BP_5	GO:0015671~oxygen transport	4	0.002265567	HBA-A1, HBB-BH1, HBA-X, HBB-Y
GOTERM_BP_5	GO:0007423~sensory organ development	16	0.002267988	MAF, TSHZ1, SOX1, CRYAB, MAFB, FGF9, PRRX1, MEIS1, SHH, FOXP2, RDH10, BCL11B, ALDH1A3, BCL2, RARB, COL8A1
GOTERM_BP_5	GO:0060537~muscle tissue development	11	0.002351612	MEF2C, TNNT2, APP, ACTC1, MYL2, PLN, ZFPM2, RARB, NR2F2, SHH, FOXP2
GOTERM_BP_5	GO:0048754~branching morphogenesis of a tube	9	0.002400231	EDNRA, RDH10, HOXA5, NPNT, BCL2, TGFBR2, IGF1, CXCL12, SHH
GOTERM_BP_5	GO:0032990~cell part morphogenesis	14	0.002842988	EGR2, ONECUT2, CXCL12, SLIT2, SHH, ALCAM, APP, EPHA7, BCL11B, BCL2, RELN, ROBO2, SEMA3A, SLITRK6
GOTERM_BP_5	GO:0048701~embryonic cranial skeleton morphogenesis	5	0.002845825	RDH10, NDST1, CHST11, TGFBR2, PRRX1
GOTERM_BP_5	GO:0048729~tissue morphogenesis	15	0.002910057	EGFR, ACTC1, NPNT, IGF1, ZEB2, SHH, TNNT2, CYP7B1, HOXB4, RDH10, HOXA5, ALDH1A3, BCL2, SEMA3C, SEMA3A
GOTERM_BP_5	GO:0048645~organ formation	4	0.002964624	RDH10, HOXA3, TGFBR2, SHH
GOTERM_BP_5	GO:0010647~positive regulation of cell communication	13	0.003062854	PTPRC, FGF9, NPNT, PRRX1, ZEB2, ITPKB, KITL, NCAM1, SHOX2, ALDH1A3, TGM2, RELN, THBS1
GOTERM_BP_5	GO:0030203~glycosaminoglycan metabolic process	6	0.003173819	CSGALNACT1, A930038C07RIK, BGN, NDST1, CHST11, DCN
GOTERM_BP_5	GO:0001656~metanephros development	7	0.003323072	RDH10, NPNT, BCL2, ROBO2, RARB, SHH, SLIT2
GOTERM_BP_5	GO:0051272~positive regulation of cell motion	6	0.003541264	PTP4A1, BCL2, ONECUT2, THBS1, CXCL12, PIK3R1

GOTERM_BP_5	GO:0031175~neuron projection development	14	0.003611705	EGR2, PRKG1, CXCL12, SLIT2, SHH, ALCAM, APP, EPHA7, BCL11B, BCL2, RELN, ROBO2, SEMA3A, SLITRK6
GOTERM_BP_5	GO:0002026~regulation of the force of heart contraction	4	0.003782487	PRKCA, SLC8A1, ADRB1, PLN
GOTERM_BP_5	GO:0048699~generation of neurons	24	0.003823321	EGR2, CORIN, SOX1, ONECUT2, TIMP2, PRKG1, MEIS1, CXCL12, SLIT2, SHH, ALCAM, NTRK3, EPHA7, APP, SERPINF1, BCL11B, BCL2, RELN, ROBO2, ID4, SEMA3A, SLITRK6, NR2F2, NEFM
GOTERM_BP_5	GO:0007167~enzyme linked receptor protein signaling pathway	16	0.00397084	PLAT, EGFR, NDST1, LTBP3, FGF9, TGFBR2, AXL, IGF1, DDR2, EPHA3, NTRK3, EPHA7, ANGPT1, PIK3R1, CSF1R, ADAM9
GOTERM_BP_5	GO:0045664~regulation of neuron differentiation	9	0.004254853	NTRK3, BCL2, ID4, ROBO2, SEMA3A, TIMP2, MEIS1, SHH, NEFM
GOTERM_BP_5	GO:0007435~salivary gland morphogenesis	5	0.004429062	EGFR, TGM2, SEMA3C, SEMA3A, SHH
GOTERM_BP_5	GO:0007517~muscle organ development	12	0.005103162	MEF2C, TNNT2, APP, ACTC1, MYL2, CRYAB, PLN, ZFPM2, RARB, NR2F2, SHH, FOXP2
GOTERM_BP_5	GO:0030003~cellular cation homeostasis	11	0.005217842	PRKCA, PTPRC, SLC8A1, APP, TRPC6, SLC24A3, PLN, BCL2, TGM2, STC1, PRNP
GOTERM_BP_5	GO:0000902~cell morphogenesis	17	0.005298848	EGFR, EGR2, LIPA, ONECUT2, CXCL12, SLIT2, SHH, ALCAM, APP, EPHA7, BCL11B, BCL2, RELN, ROBO2, SEMA3A, SLITRK6, IDUA
GOTERM_BP_5	GO:0001822~kidney development	9	0.00567791	RDH10, NPNT, BCL2, ROBO2, NID1, ADAMTS1, RARB, SHH, SLIT2
GOTERM_BP_5	GO:0050767~regulation of neurogenesis	10	0.00627542	NTRK3, SERPINF1, BCL2, ID4, ROBO2, SEMA3A, TIMP2, MEIS1, SHH, NEFM
GOTERM_BP_5	GO:0001654~eye development	11	0.006526954	MAF, RDH10, SOX1, CRYAB, BCL11B, ALDH1A3, RARB, COL8A1, MEIS1, SHH, FOXP2
GOTERM_BP_5	GO:0030902~hindbrain development	7	0.006786118	EGR2, MAFB, PLXNA2, BCL2, SHH, FOXP2, NFIB
GOTERM_BP_5	GO:0030512~negative regulation of transforming growth factor beta receptor signaling pathway	4	0.007002543	HTRA1, CHST11, ONECUT2, PRDM16
GOTERM_BP_5	GO:0045580~regulation of T cell differentiation	6	0.007012224	CD83, PTPRC, TGFBR2, VNN1, ITPKB, SHH
GOTERM_BP_5	GO:0048666~neuron development	16	0.00732144	EGR2, SOX1, ONECUT2, PRKG1, CXCL12, SLIT2, SHH, ALCAM, APP, EPHA7, BCL11B, BCL2, RELN, ROBO2, SEMA3A, SLITRK6
GOTERM_BP_5	GO:0050679~positive regulation of epithelial cell proliferation	5	0.007337791	EGFR, CYP7B1, FGF9, SHH, FOXP2
GOTERM_BP_5	GO:0007431~salivary gland development	5	0.007337791	EGFR, TGM2, SEMA3C, SEMA3A, SHH
GOTERM_BP_5	GO:0050801~ion homeostasis	16	0.007546173	PRKCA, PTPRC, GLRB, SLC8A1, EGR2, TRPC6, ATXN1, APP, SLC24A3, PLN, BCL2, SLC30A4, TGM2, STC1, PRNP, SLC4A4
GOTERM_BP_5	GO:0000904~cell morphogenesis	13	0.00760958	ALCAM, EPHA7, APP, EGR2, BCL2,

	involved in differentiation		2	BCL11B, RELN, ROBO2, SEMA3A, SLITRK6, CXCL12, SHH, SLIT2
GOTERM_BP_5	GO:0051246~regulation of protein metabolic process	18	0.00793048 6	PRKCA, EGFR, PTPRC, SOCS3, IGF1, ZEB2, PRKCE, KITL, SAMD4, SHH, RNF180, APP, HIPK3, KRT7, BCL2, JUN, DNAJC1, ADAM9
GOTERM_MF_5	GO:0004175~endopeptidase activity	21	0.00802732 5	PLAT, ADAM10, CORIN, MASP1, APH1B, MMP12, PAPPA, HTRA1, ATG4A, CTSO, FAP, BACE1, SERPINE1, ADAMTS1, ADAMTS12, PRSS23, ADAM12, PRSS35, PLAU, ADAMTS4, ADAM9
GOTERM_BP_5	GO:0045582~positive regulation of T cell differentiation	5	0.00822247 5	CD83, PTPRC, TGFBR2, VNN1, ITPKB
GOTERM_BP_5	GO:0060425~lung morphogenesis	4	0.00834609 5	RDH10, HOXA5, TGFBR2, SHH
KEGG_PATHWAY	mmu05410:Hypertrophic cardiomyopathy (HCM)	8	0.00861550 6	TNNT2, SLC8A1, ACTC1, MYL2, ITGA1, IGF1, CACNA1D, SGCB
GOTERM_BP_5	GO:0035108~limb morphogenesis	9	0.00867453 3	SHOX2, RDH10, FGF9, CHST11, PRRX1, RARB, MECOM, SHH, IDUA
GOTERM_BP_5	GO:0048703~embryonic viscerocranium morphogenesis	3	0.00892053 9	RDH10, NDST1, CHST11
GOTERM_BP_5	GO:0060438~trachea development	3	0.00892053 9	HOXA5, TGFBR2, SHH
GOTERM_BP_5	GO:0006022~aminoglycan metabolic process	6	0.00981026 9	CSGALNACT1, A930038C07RIK, BGN, NDST1, CHST11, DCN
GOTERM_BP_5	GO:0060445~branching involved in salivary gland morphogenesis	4	0.00983064 2	TGM2, SEMA3C, SEMA3A, SHH
**Cluster 2**				
**Category**	**Term**	**Coun t**	**PValue**	**Genes**
**KEGG_PATHWA Y**	**mmu00982:Drug metabolism**	**23**	**3.08E-18**	**CYP2C70, CYP2D9, GSTA2, CYP2C65, CYP2D10, CYP2C55, CYP3A16, CYP3A25, CYP3A13, CYP3A11, UGT2B1, CYP2C29, ADH7, CYP1A2, CYP2A12, UGT2B36, ADH1, FMO2, CYP3A41A, UGT2B5, CYP2D26, GSTO1, CYP2A4**
GOTERM_MF_5	GO:0070330~aromatase activity	16	1.41E-16	CYP2C70, CYP2D9, CYP2J5, CYP2D10, CYP2C55, CYP3A25, CYP3A16, CYP3A13, CYP3A11, CYP2C29, CYP1A2, CYP2A12, CYP4A12A, CYP3A41A, CYP2D26, CYP2A4
GOTERM_CC_5	GO:0042598~vesicular fraction	28	3.37E-16	CYP2J5, CYP2D9, CYP2D10, CYP3A25, CYP2F2, PTGS2, CYP2A12, RDH7, CYP4A12A, FMO2, CYP3A41A, CYP2C70, CYP2C55, CYP3A16, CYP3A13, CYP3A11, CYP2C29, EPHX1, CYP1A2, CYB5, IYD, CYP4A10, G6PC, CYP4A31, UGT2B5, CYP2D26, DIO1, SLC27A2, CYP2A4
KEGG_PATHWAY	mmu00980:Metabolism of xenobiotics by cytochrome P450	19	1.90E-14	CYP2C70, GSTA2, CYP2C65, CYP2C55, CYP3A25, CYP3A16, CYP2F2, CYP3A13, CYP3A11, UGT2B1, CYP2C29, EPHX1, ADH7,

				CYP1A2, UGT2B36, ADH1, CYP3A41A, UGT2B5, GSTO1
KEGG_PATHWAY	mmu00830:Retinol metabolism	19	3.39E-14	CYP2C70, CYP2C65, CYP2C55, CYP3A16, CYP3A25, CYP3A13, CYP3A11, UGT2B1, CYP2C29, ADH7, CYP1A2, CYP2A12, CYP4A10, UGT2B36, CYP4A12A, ADH1, CYP3A41A, CYP4A31, UGT2B5, CYP2A4
GOTERM_BP_5	GO:0030855~epithelial cell differentiation	16	9.70E-09	KRT6A, AR, KRT6B, CEBPB, PTGS2, PPARG, ESR1, EHF, PGR, KRT17, SPRR2A1, AGT, KRT14, SPRR3, KRT4, IVL
KEGG_PATHWAY	mmu00591:Linoleic acid metabolism	12	1.33E-08	CYP2C70, CYP2J5, CYP2C65, CYP2C55, CYP3A25, CYP3A16, CYP3A13, AKR1C6, CYP3A11, CYP2C29, CYP3A41A, CYP1A2
GOTERM_BP_5	GO:0002526~acute inflammatory response	13	3.34E-08	MBL1, IL6, C4B, REG3B, ORM1, C1RA, SERPINA3N, C4BP, SAA2, NUPR1, SAA1, REG3G, ORM2
GOTERM_BP_5	GO:0009888~tissue development	34	1.14E-07	KRT6A, FGF7, CRYGS, KRT6B, PTGS2, PPARG, EDN1, ELN, EHF, PGR, CD44, AGT, MYOG, KRT4, IVL, SPP1, AR, CEBPB, ACTA1, MYF5, KRTDAP, ESR1, MMP13, JUNB, KRT17, SPRR2A1, ADM, SERPINB5, KRT14, MYH11, SPRR3, GHRL, AREG, ASPRV1
GOTERM_BP_5	GO:0060429~epithelium development	21	1.92E-07	AR, KRT6A, KRT6B, CEBPB, CRYGS, PTGS2, PPARG, ESR1, EHF, PGR, ADM, SPRR2A1, CD44, KRT17, SERPINB5, AGT, KRT14, SPRR3, KRT4, AREG, IVL
GOTERM_CC_5	GO:0005624~membrane fraction	30	4.80E-07	CYP2J5, CYP2D9, CYP2D10, CYP3A25, CYP2F2, PTGS2, SLC15A2, CYP2A12, RDH7, CYP4A12A, FMO2, CYP3A41A, CYP2C70, CYP2C55, CYP3A16, CYP3A13, CYP3A11, SPHK1, CYP2C29, EPHX1, CYP1A2, CYB5, IYD, CYP4A10, G6PC, CYP4A31, UGT2B5, CYP2D26, DIO1, SLC27A2, CYP2A4
KEGG_PATHWAY	mmu00140:Steroid hormone biosynthesis	10	1.50E-06	UGT2B36, CYP3A25, CYP3A16, CYP3A13, HSD17B2, CYP3A11, UGT2B1, CYP3A41A, UGT2B5, HSD11B1
GOTERM_MF_5	GO:0004867~serine-type endopeptidase inhibitor activity	14	1.65E-06	A2M, SERPINA11, SERPINB1A, SPINK4, SPINK5, SERPINB9C, SERPINA3N, SERPINA7, SERPINB5, AGT, SERPINB2, SERPINB11, SERPINB12, SERPINB9D
**KEGG_PATHWAY**	**mmu00983:Drug metabolism**	**10**	**2.66E-06**	**CYP2A12, UGT2B36, CYP3A25, CYP3A16, CYP3A13, CYP3A11, UGT2B1, CYP3A41A, UGT2B5, CYP2A4**
GOTERM_MF_5	GO:0004175~endopeptidase activity	26	7.90E-06	MMP7, HP, MMP3, C1RA, PRSS2, PRSS3, PRSS32, 1810009J06RIK, CELA3B, CTRB1, F9, CTSR, TRY4,

				MMP13, PROC, TMPRSS4, MMP10, CTSK, CTSJ, CTSQ, BACE2, SERPINB2, 2210010C04RIK, PAPPA2, CELA2A, ASPRV1
GOTERM_BP_5	GO:0008202~steroid metabolic process	14	1.08E-05	HSD17B2, ESR1, RDH9, 1600014K23RIK, G6PC, HMGCS2, SULT1B1, AKR1C6, SAA1, CH25H, SULT1A1, APOC3, HSD11B1, SLC27A5
GOTERM_BP_5	GO:0043627~response to estrogen stimulus	7	3.02E-05	KCNMA1, SPRR2A1, ESR1, GHRL, AREG, MMP13, LCT
GOTERM_BP_5	GO:0006631~fatty acid metabolic process	14	4.45E-05	ACOX2, ACOX1, PTGS2, CYB5, ACSM3, CYP4A10, ACSM1, CYP4A12A, CH25H, HAO2, CYP4A31, ACAA1B, SLC27A2, DEGS2, SLC27A5
GOTERM_MF_5	GO:0005509~calcium ion binding	38	4.93E-05	GALNT3, MBL1, NKD2, LTBP2, TNNC2, S100A9, MYL1, MMP7, MMP3, ASGR1, C1RA, ANXA8, CALML3, RPTN, PRSS2, SFTPD, KCNMA1, 1810030J14RIK, AMY2A4, VIL1, F9, MMP13, S100A14, PROC, IGSF5, ABP1, CDH13, MMP10, ANXA10, S100B, CDH17, PLSCR2, FBLN5, CALM4, ANXA13, PROS1, CASQ2, AOC3
GOTERM_BP_5	GO:0007589~body fluid secretion	5	7.65E-05	KCNMA1, KCNN4, EDN1, GUCA2B, AGR2
GOTERM_MF_5	GO:0008236~serine-type peptidase activity	16	8.21E-05	CELA3B, CTRB1, F9, HP, TRY4, PROC, TMPRSS4, C1RA, PRSS2, PRSS3, SERPINB2, PRSS32, LTF, 2210010C04RIK, CELA2A, 1810009J06RIK
GOTERM_MF_5	GO:0004252~serine-type endopeptidase activity	15	8.72E-05	CELA3B, CTRB1, F9, HP, TRY4, PROC, TMPRSS4, C1RA, PRSS2, PRSS3, SERPINB2, PRSS32, 2210010C04RIK, CELA2A, 1810009J06RIK
GOTERM_BP_5	GO:0019752~carboxylic acid metabolic process	23	1.02E-04	ACOX2, ACOX1, PTGS2, HAL, ADH7, FBP2, CYB5, BBOX1, ACSM3, RDH9, CYP4A10, ASPA, ACSM1, G6PC, CYP4A12A, ADH1, CH25H, HAO2, CYP4A31, GSTO1, ACAA1B, SLC27A2, SLC27A5, DEGS2
GOTERM_BP_5	GO:0051048~negative regulation of secretion	6	1.30E-04	INHBB, IL6, OIT1, EDN1, GHRL, ADA
GOTERM_BP_5	GO:0031424~keratinization	6	1.56E-04	KRT6A, KRT6B, KRT17, SPRR2A1, SPRR3, IVL
**KEGG_PATHWAY**	**mmu03320:PPAR signaling pathway**	**10**	**1.66E-04**	**CYP4A10, ACOX2, ACOX1, CYP4A12A, HMGCS2, APOC3, CYP4A31, PPARG, SLC27A2, ACAA1B, SLC27A5**
GOTERM_BP_5	GO:0042035~regulation of cytokine biosynthetic process	8	1.91E-04	INHBB, IL6, MYD88, CEBPB, S100B, BCL3, TLR3, GHRL
GOTERM_BP_5	GO:0046888~negative regulation of hormone secretion	5	2.40E-04	INHBB, IL6, OIT1, EDN1, GHRL
GOTERM_BP_5	GO:0030574~collagen catabolic process	5	2.40E-04	MMP10, PRSS2, MMP7, MMP3, MMP13
KEGG_PATHWAY	mmu00590:Arachidonic acid	10	2.43E-04	CYP2C70, GPX2, CYP4A10, CYP2J5,

	metabolism			CYP2C65, CYP2C55, CYP4A12A, CBR2, PTGS2, CYP2C29, CYP4A31
GOTERM_CC_5	GO:0031226~intrinsic to plasma membrane	25	3.42E-04	KCNMA1, STEAP4, AQP9, SLC15A2, AQP5, SLC6A14, AQP4, SECTM1B, SLCO1A5, THY1, SLCO1B2, KCNN4, VSIG2, FCGR2B, SGCG, CDH17, CLEC2D, ABCC3, CP, SLC4A4, CLEC2H, KCTD14, SCARA5, KCNQ1, KCNE3
**GOTERM_BP_5**	**GO:0009913~epidermal cell differentiation**	**7**	**3.70E-04**	**KRT6A, KRT6B, KRT17, PTGS2, SPRR2A1, SPRR3, IVL**
GOTERM_BP_5	GO:0002009~morphogenesis of an epithelium	12	4.28E-04	PGR, AR, KRT6A, CRYGS, KRT6B, KRT17, ADM, CD44, SERPINB5, AGT, ESR1, AREG
GOTERM_BP_5	GO:0046883~regulation of hormone secretion	6	5.30E-04	GCG, INHBB, IL6, OIT1, EDN1, GHRL
GOTERM_BP_5	GO:0008544~epidermis development	10	5.85E-04	KRT6A, FGF7, KRT6B, KRT17, PTGS2, SPRR2A1, SPRR3, KRTDAP, IVL, ASPRV1
**GOTERM_BP_5**	**GO:0007398~ectoderm development**	**10**	**9.15E-04**	**KRT6A, FGF7, KRT6B, KRT17, PTGS2, SPRR2A1, SPRR3, KRTDAP, IVL, ASPRV1**
GOTERM_CC_5	GO:0031225~anchored to membrane	13	0.001010704	LY6C1, HFE2, ITLN1, THY1, 2210415F13RIK, LY6A, CDH13, LY6D, SEMA7A, MSLN, PSCA, CP, VNN3
GOTERM_BP_5	GO:0022612~gland morphogenesis	8	0.001012412	PGR, AR, IL6, FGF7, CD44, SERPINB5, ESR1, AREG
GOTERM_CC_5	GO:0005887~integral to plasma membrane	23	0.00112404	KCNMA1, STEAP4, AQP9, SLC15A2, AQP5, SLC6A14, AQP4, SECTM1B, SLCO1A5, SLCO1B2, KCNN4, VSIG2, FCGR2B, SGCG, CDH17, CLEC2D, ABCC3, SLC4A4, CLEC2H, KCTD14, SCARA5, KCNQ1, KCNE3
GOTERM_BP_5	GO:0019724~B cell mediated immunity	7	0.001363161	MBL1, C1RA, C4BP, MYD88, FCGR2B, C4B, BCL3
GOTERM_BP_5	GO:0045109~intermediate filament organization	4	0.001637534	KRT6A, KRT6B, KRT17, KRT14
GOTERM_BP_5	GO:0048754~branching morphogenesis of a tube	8	0.001836555	PGR, AR, CD44, AGT, EDN1, ESR1, AREG, MYCN
GOTERM_BP_5	GO:0048729~tissue morphogenesis	13	0.001837338	PGR, AR, KRT6A, FGF7, CRYGS, KRT6B, KRT17, ADM, CD44, SERPINB5, AGT, ESR1, AREG
GOTERM_BP_5	GO:0042036~negative regulation of cytokine biosynthetic process	4	0.002055346	INHBB, IL6, BCL3, GHRL
GOTERM_BP_5	GO:0031348~negative regulation of defense response	5	0.002282003	GPX2, FCGR2B, CLEC2D, GHRL, ADA
GOTERM_BP_5	GO:0051046~regulation of secretion	9	0.002485776	GCG, INHBB, KCNN4, IL6, RAB3D, OIT1, EDN1, GHRL, ADA
KEGG_PATHWAY	mmu04610:Complement and coagulation cascades	8	0.002904576	MBL1, C1RA, A2M, C4BP, C4B, F9, PROS1, PROC
GOTERM_MF_5	GO:0004062~aryl sulfotransferase activity	3	0.002910357	SULT1B1, SULT1A1, SULT1D1
GOTERM_BP_5	GO:0002449~lymphocyte mediated immunity	7	0.003040959	MBL1, C1RA, C4BP, MYD88, FCGR2B, C4B, BCL3
GOTERM_BP_5	GO:0042742~defense response to bacterium	8	0.00426693	MBL1, DEFA24, MYD88, DEFA5, DEFA4, PGLYRP1, BCL3, DEFB1

GOTERM_BP_5	GO:0060745~mammary gland branching involved in pregnancy	3	0.005034259	PGR, AR, ESR1
GOTERM_BP_5	GO:0015718~monocarboxylic acid transport	4	0.0051049	PPARG, ABCC3, SLC27A2, SLC27A5
GOTERM_BP_5	GO:0060444~branching involved in mammary gland duct morphogenesis	4	0.0051049	PGR, AR, ESR1, AREG
KEGG_PATHWAY	mmu00071:Fatty acid metabolism	6	0.005519141	CYP4A10, ACOX1, CYP4A12A, ADH1, CYP4A31, ADH7, ACAA1B
GOTERM_BP_5	GO:0001890~placenta development	7	0.005924314	CEBPB, ADM, SOCS3, PPARG, GHRL, ADA, JUNB
GOTERM_BP_5	GO:0030850~prostate gland development	5	0.006003135	AR, CD44, SERPINB5, ESR1, AHR
GOTERM_BP_5	GO:0006508~proteolysis	32	0.00643009	MBL1, MMP7, HP, MMP3, C1RA, PRSS2, PRSS3, PRSS32, 1810009J06RIK, CELA3B, C4B, SOCS3, CTRB1, F9, CTSR, TRY4, MMP13, PROC, TMPRSS4, MMP10, CTSK, C4BP, CTSJ, CTSQ, BACE2, UBD, 2210010C04RIK, PAPPA2, CELA2A, CPB1, PMEPA1, ASPRV1
GOTERM_BP_5	GO:0050678~regulation of epithelial cell proliferation	6	0.006555631	PGR, AR, IL6, FGF7, SERPINB5, KRT4
GOTERM_BP_5	GO:0007588~excretion	4	0.006815642	KCNMA1, AGT, EDN1, GUCA2B
GOTERM_BP_5	GO:0045073~regulation of chemokine biosynthetic process	3	0.006960803	IL6, MYD88, TLR3
GOTERM_CC_5	GO:0019898~extrinsic to membrane	19	0.007336124	CYP2C70, CYP2D9, CYP2J5, CYP2D10, CYP2C55, CYP3A16, CYP3A25, PTGS2, CYP2F2, CYP3A13, CYP3A11, CYP2C29, CYP1A2, CYP2A12, CYP4A10, CYP4A12A, CYP3A41A, CYP4A31, CYP2D26, CYP2A4
GOTERM_BP_5	GO:0050728~negative regulation of inflammatory response	4	0.007783595	GPX2, FCGR2B, GHRL, ADA
GOTERM_BP_5	GO:0050796~regulation of insulin secretion	4	0.007783595	GCG, INHBB, OIT1, GHRL
KEGG_PATHWAY	mmu00040:Pentose and glucuronate interconversions	4	0.008605693	AKR1B8, UGT2B36, UGT2B1, UGT2B5
KEGG_PATHWAY	mmu00150:Androgen and estrogen metabolism	5	0.009497256	UGT2B36, HSD17B2, UGT2B1, UGT2B5, HSD11B1
GOTERM_BP_5	GO:0008285~negative regulation of cell proliferation	11	0.009870056	AR, NUPR1, PTGS2, FCGR2B, AGT, PPARG, GHRL, KRT4, TFF1, SCGB1A1, DPT
GOTERM_MF_5	GO:0008009~chemokine activity	5	0.009972034	CXCL1, CCL12, CXCL14, CXCL5, CCL6
**Cluster 3**				
**Category**	**Term**	**Count**	**PValue**	**Genes**
**GOTERM_BP_5**	**GO:0007126~meiosis**	**11**	**5.73E-07**	**REC8, TEX15, SYCP3, RSPH1, MRE11A, MAEL, SYCE2, CPEB1, PTTG1, SMC1B, SUV39H2**
GOTERM_BP_5	GO:0051327~M phase of meiotic cell cycle	11	5.73E-07	REC8, TEX15, SYCP3, RSPH1, MRE11A, MAEL, SYCE2, CPEB1, PTTG1, SMC1B, SUV39H2

GOTERM_BP_5	GO:0043414~biopolymer methylation	10	8.37E-07	PRMT3, 2410022L05RIK, DNMT3A, PRDM5, MAEL, ETF1, DNMT3B, MPHOSPH8, HELLS, SUV39H2
GOTERM_BP_5	GO:0070192~chromosome organization involved in meiosis	6	3.35E-06	REC8, TEX15, SYCP3, MAEL, SYCE2, CPEB1
GOTERM_BP_5	GO:0007129~synapsis	6	3.35E-06	REC8, TEX15, SYCP3, MAEL, SYCE2, CPEB1
**GOTERM_CC_5**	**GO:0005634~nucleus**	**89**	**7.74E-06**	**TEX19.2, CRXOS1, NR6A1, MAEL, GM13139, LEMD1, PTTG1, HMGN5, ZIC2, KIF2C, GABPB1, G2E3, HSF2, PHTF2, MAGOHB, MYB, PMS1, TERT, CDC7, SGK1, GEN1, NOL8, DMRT3, MTA3, OTX2, EOMES, GSTT2, CCNC, GRHL2, MYCN, ELL2, PNPLA7, REC8, SYCP3, TCFL5, AA467197, SERBP1, PRDM5, SIX1, ESRP1, RAD18, CPSF6, BRDT, MED1, MRE11A, STK17B, RRAGD, OBFC2A, HESX1, MORC3, BUB1, WDR12, POU3F1, GM4924, DNMT3B, HELLS, DNMT3A, TCF7, DTX1, EME1, KIF18A, SYCE2, GM4349, KLF15, DPPA3, CAPN3, RPF2, ZFP709, DDX4, SUV39H2, 2410022L05RIK, DUSP4, ZFP106, UBTFL1, PHF19, ZIC5, GTF2F2, IRF1, PHF21A, KDM4C, RAD54B, 2010317E24RIK, ALKBH2, RPP40, DNAJB6, MPHOSPH8, PLEKHA1, SMC1B, ZBTB8A**
**GOTERM_BP_5**	**GO:0007127~meiosis I**	**7**	**1.01E-05**	**REC8, TEX15, SYCP3, MAEL, SYCE2, CPEB1, PTTG1**
GOTERM_CC_5	GO:0044454~nuclear chromosome part	10	1.16E-05	DNMT3A, REC8, SYCP3, MAEL, BUB1, SYCE2, RAD18, DNMT3B, SMC1B, SUV39H2
GOTERM_CC_5	GO:0000228~nuclear chromosome	10	4.23E-05	DNMT3A, REC8, SYCP3, MAEL, BUB1, SYCE2, RAD18, DNMT3B, SMC1B, SUV39H2
GOTERM_BP_5	GO:0000279~M phase	15	6.85E-05	MRE11A, HAUS2, MAEL, KIF18A, SYCE2, PTTG1, CPEB1, SUV39H2, REC8, TEX15, SYCP3, RSPH1, BUB1, HELLS, SMC1B
GOTERM_MF_5	GO:0004089~carbonate dehydratase activity	5	1.02E-04	CAR13, CAR14, CAR4, CAR2, CAR3
GOTERM_CC_5	GO:0044427~chromosomal part	15	1.24E-04	DNMT3A, AURKC, MAEL, SYCE2, HMGN5, SUV39H2, REC8, SYCP3, BUB1, RAD18, DNMT3B, MPHOSPH8, TERT, HELLS, SMC1B
GOTERM_BP_5	GO:0006259~DNA metabolic process	18	1.42E-04	DNMT3A, GEN1, MRE11A, EME1, MAEL, PTTG1, OBFC2A, DNA2, RAD18, RAD54B, ALKBH2, DNMT3B, MPHOSPH8, PMS1, TERT, HELLS, MED1, SGIP1
KEGG_PATHWAY	mmu00910:Nitrogen metabolism	5	1.78E-04	CAR13, CAR14, CAR4, CAR2, CAR3
GOTERM_CC_5	GO:0005694~chromosome	16	2.23E-04	DNMT3A, AURKC, MAEL, SYCE2, GM4349, HMGN5, SUV39H2, REC8,

				SYCP3, BUB1, RAD18, DNMT3B, MPHOSPH8, HELLS, TERT, SMC1B
GOTERM_BP_5	GO:0007130~synaptonemal complex assembly	4	2.40E-04	TEX15, SYCP3, SYCE2, CPEB1
GOTERM_BP_5	GO:0006304~DNA modification	5	3.70E-04	DNMT3A, MAEL, DNMT3B, MPHOSPH8, HELLS
GOTERM_CC_5	GO:0000792~heterochromatin	6	6.43E-04	DNMT3A, MAEL, RAD18, DNMT3B, HELLS, SUV39H2
KEGG_PATHWAY	mmu00270:Cysteine and methionine metabolism	5	7.46E-04	LDHB, DNMT3A, MTR, DNMT3B, SMS
GOTERM_CC_5	GO:0000775~chromosome, centromeric region	8	8.26E-04	DNMT3A, REC8, BUB1, AURKC, DNMT3B, HELLS, SMC1B, SUV39H2
GOTERM_CC_5	GO:0000795~synaptonemal complex	4	0.002423321	REC8, SYCP3, SYCE2, SMC1B
GOTERM_BP_5	GO:0016568~chromatin modification	11	0.002430774	2410022L05RIK, DNMT3A, PHF16, PRDM5, PHF21A, KDM4C, GM4349, BRDT, DNMT3B, HELLS, SUV39H2
GOTERM_BP_5	GO:0007140~male meiosis	4	0.002489686	REC8, TEX15, MAEL, SUV39H2
GOTERM_CC_5	GO:0000794~condensed nuclear chromosome	5	0.002535465	REC8, SYCP3, BUB1, SYCE2, SMC1B
GOTERM_CC_5	GO:0000800~lateral element	3	0.003739904	REC8, SYCP3, SMC1B
GOTERM_BP_5	GO:0006281~DNA repair	10	0.005247435	DNA2, GEN1, MRE11A, EME1, RAD18, RAD54B, PTTG1, OBFC2A, ALKBH2, PMS1
GOTERM_CC_5	GO:0000790~nuclear chromatin	5	0.006732522	DNMT3A, MAEL, RAD18, DNMT3B, SUV39H2
GOTERM_BP_5	GO:0019219~regulation of nucleobase, nucleoside, nucleotide and nucleic acid metabolic process	49	0.007443685	CRXOS1, NR6A1, MAEL, GM13139, HMGN5, GABPB1, HSF2, PHTF2, MYB, DMRT3, MTA3, OTX2, EOMES, CCNC, GRHL2, MYCN, ELL2, TCFL5, PRDM5, SIX1, ESRP1, RAD18, BRDT, MED1, CALCR, HESX1, POU3F1, GM4924, DNMT3B, HELLS, GUCA1A, DNMT3A, TCF7, 6030426L16RIK, GM4349, KLF15, ZFP709, SUV39H2, 2410022L05RIK, DNA2, PHF19, TEX15, GTF2F2, IRF1, PHF21A, KDM4C, RAD54B, DNAJB6, ZBTB8A
GOTERM_CC_5	GO:0000785~chromatin	8	0.007639915	DNMT3A, MAEL, RAD18, HMGN5, DNMT3B, MPHOSPH8, HELLS, SUV39H2
GOTERM_CC_5	GO:0044428~nuclear part	28	0.010733657	NR6A1, MAEL, LEMD1, OBFC2A, HMGN5, G2E3, MORC3, BUB1, WDR12, POU3F1, DNMT3B, DNMT3A, NOL8, EME1, SYCE2, RPF2, SUV39H2, ELL2, REC8, ZFP106, SYCP3, SIX1, GTF2F2, PHF21A, RAD18, RPP40, MED1, SMC1B
GOTERM_BP_5	GO:0007283~spermatogenesis	10	0.012554559	DNMT3A, REC8, TEX15, SYCP3, TCFL5, HSF2, NR6A1, MAEL, RAD18, NLRP14
GOTERM_BP_5	GO:0010468~regulation of gene expression	49	0.013377863	CRXOS1, NR6A1, MAEL, CPEB1, GM13139, HMGN5, GABPB1, HSF2, PHTF2, MYB, DMRT3, MTA3, OTX2,

				EOMES, CCNC, GRHL2, MYCN, ELL2, TCFL5, PRDM5, SIX1, ESRP1, BRDT, MED1, CALCR, HESX1, MORC3, POU3F1, GM4924, DNMT3B, HELLS, DNMT3A, TCF7, 6030426L16RIK, GM4349, KLF15, ZFP709, SUV39H2, 2410022L05RIK, PHF19, GTF2F2, PLCG2, IRF1, PHF21A, KDM4C, RAD54B, MPHOSPH8, DNAJB6, ZBTB8A
KEGG_PATHWAY	mmu04114:Oocyte meiosis	6	0.01508663	REC8, MAPK12, BUB1, CPEB1, PTTG1, SMC1B
GOTERM_MF_5	GO:0004519~endonuclease activity	6	0.015464285	DNA2, GEN1, MRE11A, EME1, ALKBH2, RPP40
GOTERM_BP_5	GO:0006006~glucose metabolic process	7	0.016674299	ALDOA, PDK1, LDHB, PYGL, GM5506, PPP1R1A, PFKM
GOTERM_CC_5	GO:0005874~microtubule	9	0.017615224	2310057J16RIK, KIFC2, KIF2C, HAUS2, TUBA3A, NINL, KIF18A, TUBE1, JAKMIP1
GOTERM_BP_5	GO:0040029~regulation of gene expression, epigenetic	5	0.019028635	DNMT3A, MAEL, DNMT3B, MPHOSPH8, HELLS
GOTERM_MF_5	GO:0032559~adenyl ribonucleotide binding	31	0.021521461	MYO5A, KIFC2, IDE, STK17B, AURKC, ATP10D, KIF2C, MORC3, BUB1, PMS1, HELLS, ACSL6, PDK1, CDC7, SGK1, PDXK, KIF18A, PFKM, PIM2, DDX4, RIMKLB, NUBPL, DNA2, CBWD1, MAPK12, MAPK4, GTF2F2, RAD54B, NLRP14, UBE2T, SMC1B
GOTERM_MF_5	GO:0008757~S-adenosylmethionine-dependent methyltransferase activity	5	0.022683661	DNMT3A, MTR, GM4349, DNMT3B, SUV39H2
GOTERM_CC_5	GO:0005741~mitochondrial outer membrane	5	0.024018936	TOMM20, CYB5B, PMAIP1, GPAT2, ACSL6
**KEGG_PATHWAY**	**mmu00480:Glutathione metabolism**	**4**	**0.027984638**	**GGCT, GSTT2, GPX7, SMS**
GOTERM_BP_5	GO:0002285~lymphocyte activation during immune response	3	0.030746595	PLCG2, EOMES, RAB27A
GOTERM_CC_5	GO:0031968~organelle outer membrane	5	0.031428983	TOMM20, CYB5B, PMAIP1, GPAT2, ACSL6
**GOTERM_CC_5**	**GO:0043073~germ cell nucleus**	**3**	**0.03596978**	**REC8, SYCP3, TCFL5**
GOTERM_BP_5	GO:0019318~hexose metabolic process	7	0.037267902	ALDOA, PDK1, LDHB, PYGL, GM5506, PPP1R1A, PFKM
GOTERM_MF_5	GO:0016462~pyrophosphatase activity	16	0.037691006	MYO5A, KIFC2, EIF2S3X, ENPP3, TUBA3A, KIF18A, ATP10D, DDX4, KIF2C, DNA2, GTF2F2, TUBE1, RAD54B, GNG3, HELLS, RAB27A
GOTERM_BP_5	GO:0031326~regulation of cellular biosynthetic process	47	0.039786038	CALCR, CRXOS1, NR6A1, MAEL, GM13139, CPEB1, HMGN5, HESX1, GABPB1, HSF2, PHTF2, POU3F1, MYB, GM4924, DNMT3B, HELLS, DNMT3A, GUCA1A, TCF7, DMRT3, 6030426L16RIK, MTA3, OTX2, EOMES, GM4349, CCNC, KLF15, ZFP709, GRHL2, SUV39H2, ELL2, MYCN, 2410022L05RIK, DNA2,

				PHF19, TCFL5, SIX1, GTF2F2, PRDM5, IRF1, BRDT, KDM4C, PHF21A, RAD54B, DNAJB6, MED1, ZBTB8A
GOTERM_BP_5	GO:0042063~gliogenesis	4	0.040053845	FGF5, PLP1, DTX1, POU3F1
GOTERM_BP_5	GO:0019320~hexose catabolic process	4	0.040053845	ALDOA, LDHB, GM5506, PFKM
GOTERM_BP_5	GO:0045814~negative regulation of gene expression, epigenetic	3	0.040372917	DNMT3A, DNMT3B, HELLS
GOTERM_MF_5	GO:0009008~DNA-methyltransferase activity	2	0.041958523	DNMT3A, DNMT3B
GOTERM_BP_5	GO:0045449~regulation of transcription	43	0.042268561	CRXOS1, NR6A1, MAEL, GM13139, HMGN5, HESX1, GABPB1, HSF2, PHTF2, POU3F1, MYB, GM4924, DNMT3B, HELLS, DNMT3A, TCF7, DMRT3, 6030426L16RIK, MTA3, OTX2, EOMES, GM4349, CCNC, KLF15, ZFP709, GRHL2, SUV39H2, ELL2, MYCN, 2410022L05RIK, PHF19, TCFL5, SIX1, GTF2F2, PRDM5, IRF1, KDM4C, PHF21A, BRDT, RAD54B, DNAJB6, ZBTB8A, MED1
GOTERM_BP_5	GO:0032402~melanosome transport	2	0.043126456	MYO5A, RAB27A
GOTERM_BP_5	GO:0046365~monosaccharide catabolic process	4	0.044025989	ALDOA, LDHB, GM5506, PFKM
GOTERM_CC_5	GO:0015630~microtubule cytoskeleton	12	0.045535249	2310057J16RIK, KIFC2, KIF2C, CEP72, HAUS2, TUBA3A, CEP78, NINL, KIF18A, AURKC, TUBE1, JAKMIP1
KEGG_PATHWAY	mmu03440:Homologous recombination	3	0.045662129	MRE11A, EME1, RAD54B
**Cluster 4**				
**Category**	**Term**	**Count**	**PValue**	**Genes**
GOTERM_BP_5	GO:0009887~organ morphogenesis	47	3.07E-15	FRAS1, WNT5A, FGFR2, FGFR3, THRA, NRP1, EFNA1, CSF1, TBX20, FHL2, ZEB2, COL2A1, TPM1, MMP2, TGFB2, ALDH1A2, HOXC8, WNT4, GPC3, FLI1, CD44, SMARCD3, CXCR4, HAND2, PLXND1, TBX2, FOXA1, TBX4, IGF2, ISL1, PROX1, TNNI1, COL5A1, SLIT3, HOXD9, FOXF1A, HOXD8, HOXB2, FREM2, NAB1, HOXB6, PDGFRA, TGFBR3, PDGFRB, FOXC2, PBX1, TGFB1I1
GOTERM_BP_5	GO:0009888~tissue development	46	1.87E-13	FRAS1, LOR, WNT5A, FGFR2, FGFR3, NRP1, THRA, CSF1, FHL2, ZEB2, COL2A1, TPM1, ITGB1, TGFB2, ALDH1A2, APP, WNT4, GPC3, CD44, HAND2, GATA6, LHX1, PLXND1, NFATC1, BMP1, TBX2, FOXA1, TBX4, CYP26A1, ISL1, PROX1, KITL, TNNI1, COL5A1, KDR, HOXD9, FOXF1A, HOXB2, FREM2, TGFBR3, PDGFRB, FOXC2, PBX1, TGFB1I1, BMP5,

				EPB4.1L5
**GOTERM_BP_5**	**GO:0001568~blood vessel development**	**27**	**4.74E-12**	**FGFR2, NRP1, TBX20, ELK3, CXCL12, MMP2, TGFB2, SEMA5A, ALDH1A2, MYOCD, HAND2, CXCR4, HEY2, PLXND1, TBX4, ITGA4, MMP14, ARHGAP24, PROX1, COL5A1, KDR, FOXF1A, LAMA4, ECSCR, FOXC2, TGFBR3, TNFAIP2**
**GOTERM_BP_5**	**GO:0001944~vasculature development**	**27**	**8.28E-12**	**FGFR2, NRP1, TBX20, ELK3, CXCL12, MMP2, TGFB2, SEMA5A, ALDH1A2, MYOCD, HAND2, CXCR4, HEY2, PLXND1, TBX4, ITGA4, MMP14, ARHGAP24, PROX1, COL5A1, KDR, FOXF1A, LAMA4, ECSCR, FOXC2, TGFBR3, TNFAIP2**
GOTERM_BP_5	GO:0048754~branching morphogenesis of a tube	17	5.44E-11	WNT5A, NRP1, CSF1, FOXA1, TBX20, MMP14, CXCL12, KDR, SEMA5A, FOXF1A, WNT4, GPC3, CD44, CXCR4, FOXC2, PBX1, PLXND1
GOTERM_BP_5	GO:0048729~tissue morphogenesis	25	1.05E-10	FRAS1, FGFR2, WNT5A, FGFR3, NRP1, CSF1, FOXA1, TBX4, ZEB2, PROX1, TPM1, TNNI1, TGFB2, ALDH1A2, FOXF1A, WNT4, GPC3, HOXB2, CD44, FREM2, TGFBR3, FOXC2, PBX1, TGFB1I1, PLXND1
GOTERM_BP_5	GO:0048514~blood vessel morphogenesis	22	6.45E-10	FGFR2, NRP1, TBX4, TBX20, ITGA4, ELK3, ARHGAP24, MMP14, CXCL12, PROX1, KDR, TGFB2, SEMA5A, FOXF1A, MYOCD, HAND2, CXCR4, HEY2, ECSCR, FOXC2, PLXND1, TNFAIP2
GOTERM_MF_5	GO:0005509~calcium ion binding	44	1.38E-09	FRAS1, SRI, PXDN, LTBP1, GALNT7, TNNC1, MYL3, MMP2, TPM4, MYL9, PRRG1, RASGRP3, SLC25A24, FBN2, BMP1, EFEMP2, FBN1, EFEMP1, COLEC12, ITGA4, COLEC11, PCDH7, CAPN2, MMP14, ANXA5, ANXA3, SLIT2, ITPR2, PCDH18, SLIT3, FBLN1, LRP1, FBLN2, FREM2, NUCB2, PLSCR3, FKBP14, DSC2, VCAN, MCC, SGCE, FKBP10, RCN3, CDH11
GOTERM_BP_5	GO:0002009~morphogenesis of an epithelium	20	2.41E-09	FRAS1, WNT5A, FGFR2, FGFR3, NRP1, CSF1, FOXA1, TBX4, ZEB2, PROX1, ALDH1A2, WNT4, FOXF1A, GPC3, HOXB2, CD44, FREM2, PBX1, TGFB1I1, PLXND1
GOTERM_BP_5	GO:0003007~heart morphogenesis	14	2.78E-09	TBX2, TBX20, FHL2, COL2A1, ISL1, TPM1, PROX1, TNNI1, COL5A1, ALDH1A2, HAND2, SMARCD3, FOXC2, TGFBR3
**GOTERM_BP_5**	**GO:0007507~heart development**	**22**	**5.64E-09**	**NRP1, TBX2, TBX20, FHL2, COL2A1, ITGA4, ISL1, PROX1, ITGB1, TPM1, COL5A1, TNNI1, TGFB2, ALDH1A2, MYOCD, SMARCD3, GATA6, HAND2, FOXC2, TGFBR3, VCAN, NFATC1**
**GOTERM_BP_5**	**GO:0060429~epithelium**	**23**	**3.70E-08**	**FRAS1, WNT5A, LOR, FGFR2, NRP1,**

	**development**			**FGFR3, CSF1, FOXA1, TBX4, ZEB2, PROX1, KDR, ALDH1A2, WNT4, FOXF1A, GPC3, HOXB2, CD44, GATA6, FREM2, PBX1, TGFB1I1, PLXND1**
GOTERM_BP_5	GO:0001525~angiogenesis	16	8.54E-08	FGFR2, NRP1, TBX4, TBX20, ELK3, ARHGAP24, MMP14, CXCL12, KDR, SEMA5A, CXCR4, HAND2, ECSCR, FOXC2, PLXND1, TNFAIP2
GOTERM_BP_5	GO:0048705~skeletal system morphogenesis	15	4.14E-07	FGFR2, THRA, TBX4, COL2A1, MMP2, TGFB2, HOXD9, HOXC8, HOXB2, HOXD8, NAB1, HOXB6, PDGFRA, FOXC2, PDGFRB
GOTERM_BP_5	GO:0048738~cardiac muscle tissue development	10	2.07E-06	TBX2, GATA6, TGFBR3, FHL2, FOXC2, PROX1, TPM1, ITGB1, TNNI1, TGFB2
**GOTERM_BP_5**	**GO:0030324~lung development**	**13**	**2.71E-06**	**WNT5A, FGFR2, LIPA, TBX4, FOXA1, MMP14, KDR, ALDH1A2, CTTNBP2, FOXF1A, GATA6, PDGFRA, TES**
GOTERM_BP_5	GO:0030323~respiratory tube development	13	3.27E-06	WNT5A, FGFR2, LIPA, TBX4, FOXA1, MMP14, KDR, ALDH1A2, CTTNBP2, FOXF1A, GATA6, PDGFRA, TES
**GOTERM_BP_5**	**GO:0048762~mesenchymal cell differentiation**	**9**	**5.98E-06**	**FGFR2, ALDH1A2, HAND2, FOXC2, CYP26A1, ZEB2, ISL1, KITL, NFATC1**
GOTERM_BP_5	GO:0001569~patterning of blood vessels	7	6.81E-06	SEMA5A, NRP1, CXCR4, TBX20, FOXC2, PLXND1, CXCL12
GOTERM_BP_5	GO:0060485~mesenchyme development	9	7.00E-06	FGFR2, ALDH1A2, HAND2, FOXC2, CYP26A1, ZEB2, ISL1, KITL, NFATC1
GOTERM_BP_5	GO:0048732~gland development	16	1.27E-05	WNT5A, FGFR2, NRP1, TBX2, CSF1, FOXA1, ISL1, HOXD9, CTTNBP2, CCND1, WNT4, CD44, HOXA9, PBX1, PLXND1, TES
GOTERM_BP_5	GO:0007517~muscle organ development	15	1.51E-05	TBX2, FHL2, TPM1, ITGB1, PROX1, TNNI1, TGFB2, HOXD9, APP, TAGLN, GATA6, FOXC2, PDGFRB, TGFBR3, UNC45B
GOTERM_BP_5	GO:0009952~anterior/posterior pattern formation	14	1.52E-05	CER1, CYP26A1, ZEB2, ZBTB16, HOXD9, ALDH1A2, HOXC8, HOXB2, HOXD8, LHX1, HOXB6, HOXA9, FOXC2, PBX1
GOTERM_CC_5	GO:0005604~basement membrane	10	1.73E-05	FRAS1, LAMA4, FBLN1, HMCN1, FREM2, VWC2, COL2A1, TIMP2, COL5A1, COL4A6
**GOTERM_BP_5**	**GO:0060537~muscle tissue development**	**13**	**2.22E-05**	**TBX2, FHL2, TPM1, ITGB1, PROX1, TNNI1, TGFB2, HOXD9, APP, GATA6, FOXC2, PDGFRB, TGFBR3**
GOTERM_BP_5	GO:0009966~regulation of signal transduction	31	3.36E-05	FGFR2, CER1, FGFR3, EFNA1, CSF1, AKAP13, RABGAP1L, ZEB2, TIMP2, RGS10, WNT4, APP, GPC3, RASGRP3, HEY2, VWC2, RAMP2, TBC1D2B, VAV3, ADAMTS20, ARHGEF17, SOCS5, KITL, FARP2, CCND1, LRP1, DKK1, BMPER, RGS3, RGS5, ARAP3
**GOTERM_BP_5**	**GO:0014031~mesenchymal cell development**	**8**	**4.31E-05**	**ALDH1A2, HAND2, FOXC2, CYP26A1, ZEB2, ISL1, KITL, NFATC1**
**GOTERM_BP_5**	**GO:0014033~neural crest cell differentiation**	**7**	**4.80E-05**	**ALDH1A2, HAND2, FOXC2, CYP26A1, ZEB2, ISL1, KITL**

**GOTERM_BP_5**	**GO:0014032~neural crest cell development**	**7**	**4.80E-05**	**ALDH1A2, HAND2, FOXC2, CYP26A1, ZEB2, ISL1, KITL**
GOTERM_BP_5	GO:0009968~negative regulation of signal transduction	14	4.92E-05	CER1, SOCS5, RGS10, WNT4, CCND1, LRP1, GPC3, BMPER, DKK1, RGS3, RGS5, HEY2, VWC2, ARAP3
**GOTERM_BP_5**	**GO:0014706~striated muscle tissue development**	**12**	**5.79E-05**	**HOXD9, APP, TBX2, GATA6, TGFBR3, FHL2, FOXC2, PROX1, TPM1, ITGB1, TNNI1, TGFB2**
GOTERM_BP_5	GO:0030879~mammary gland development	10	6.33E-05	FGFR2, HOXD9, WNT5A, CTTNBP2, CCND1, WNT4, TBX2, CSF1, HOXA9, TES
KEGG_PATHWAY	mmu04510:Focal adhesion	14	6.91E-05	VAV3, COL2A1, ITGA4, CAPN2, ITGB1, COL4A6, COL5A1, MYL9, KDR, CCND1, LAMA4, PDGFRA, PDGFRB, PARVA
**GOTERM_BP_5**	**GO:0022008~neurogenesis**	**26**	**1.07E-04**	**FGFR3, NRP1, EFNA1, TIMP2, CXCL12, TGFB2, SEMA5A, ALDH1A2, CTTNBP2, HOXC8, APP, CXCR4, LHX1, VWC2, RUNX1, TES, FOXA1, ISL1, MMP14, SLIT2, FARP2, SLIT3, HOXD9, CDKN1C, NAB1, PBX1**
GOTERM_BP_5	GO:0010648~negative regulation of cell communication	14	1.16E-04	CER1, SOCS5, RGS10, WNT4, CCND1, LRP1, GPC3, BMPER, DKK1, RGS3, RGS5, HEY2, VWC2, ARAP3
**GOTERM_BP_5**	**GO:0035108~limb morphogenesis**	**11**	**1.22E-04**	**FGFR2, HOXD9, WNT5A, ALDH1A2, DKK1, TBX4, HOXA9, PBX1, COL2A1, FBN2, ZBTB16**
KEGG_PATHWAY	mmu04512:ECM-receptor interaction	9	1.39E-04	LAMA4, SDC1, CD44, COL2A1, ITGA4, ITGB1, COL5A1, SDC2, COL4A6
**KEGG_PATHWAY**	**mmu04360:Axon guidance**	**11**	**1.46E-04**	**SEMA5A, NRP1, RGS3, CXCR4, EFNA1, UNC5C, CXCL12, ITGB1, SLIT2, SLIT3, NFATC1**
**GOTERM_BP_5**	**GO:0060348~bone development**	**11**	**1.51E-04**	**FGFR2, THRA, BMP1, NAB1, FHL2, FOXC2, COL2A1, IGF2, MMP14, MMP2, BMP5**
GOTERM_CC_5	GO:0016323~basolateral plasma membrane	12	1.54E-04	CTTNBP2, CD44, STARD8, SH3KBP1, DSP, SLCO3A1, MSN, TGFB1I1, NEXN, ARHGAP24, PARVA, TES
GOTERM_BP_5	GO:0007167~enzyme linked receptor protein signaling pathway	17	1.57E-04	FGFR2, NRP1, LTBP1, FGFR3, PTPRE, EFNA1, SOCS5, KDR, TGFB2, ROR1, PDGFRA, FOXC2, PDGFRB, TGFBR3, BAMBI, GFRA2, GHR
GOTERM_BP_5	GO:0001656~metanephros development	8	1.70E-04	FGFR2, WNT4, GPC3, CD44, LHX1, FOXC2, PBX1, SLIT2
GOTERM_CC_5	GO:0070161~anchoring junction	11	2.19E-04	CTTNBP2, STARD8, SH3KBP1, DSP, DSC2, TGFB1I1, NEXN, ARHGAP24, PARVA, TES, EPB4.1L5
GOTERM_BP_5	GO:0048699~generation of neurons	24	2.22E-04	NRP1, FGFR3, EFNA1, FOXA1, TIMP2, ISL1, CXCL12, SLIT2, TGFB2, FARP2, SLIT3, CDKN1C, HOXD9, SEMA5A, ALDH1A2, CTTNBP2, APP, HOXC8, LHX1, CXCR4, VWC2, PBX1, RUNX1, TES
**GOTERM_BP_5**	**GO:0022612~gland morphogenesis**	**9**	**3.12E-04**	**FGFR2, WNT5A, WNT4, NRP1, CD44, TBX2, CSF1, FOXA1, PLXND1**
**GOTERM_BP_5**	**GO:0001822~kidney**	**10**	**3.40E-04**	**FGFR2, WNT4, GPC3, CD44, LHX1,**

	**development**			**PDGFRB, FOXC2, PBX1, ZBTB16, SLIT2**
GOTERM_BP_5	GO:0045596~negative regulation of cell differentiation	13	3.63E-04	CCND1, WNT4, NRP1, LMO2, TGFBR3, PBX1, ZBTB16, TGFB1I1, SOCS5, ISL1, ITGB1, TWIST2, TGFB2
GOTERM_CC_5	GO:0030055~cell-substrate junction	8	3.91E-04	CTTNBP2, STARD8, SH3KBP1, TGFB1I1, NEXN, ARHGAP24, PARVA, TES
GOTERM_BP_5	GO:0055008~cardiac muscle tissue morphogenesis	5	4.17E-04	TGFBR3, FOXC2, PROX1, TPM1, TNNI1
GOTERM_BP_5	GO:0060415~muscle tissue morphogenesis	5	4.17E-04	TGFBR3, FOXC2, PROX1, TPM1, TNNI1
GOTERM_BP_5	GO:0016477~cell migration	15	4.21E-04	CER1, VAV3, NRP1, ZEB2, ITGA4, MMP14, ISL1, CXCL12, ITGB1, KITL, KDR, TGFB2, CTTNBP2, CXCR4, TES
GOTERM_BP_5	GO:0045637~regulation of myeloid cell differentiation	7	5.74E-04	TAL1, LMO2, ETS1, CSF1, ZBTB16, RUNX1, KITL
GOTERM_BP_5	GO:0007417~central nervous system development	19	5.91E-04	WNT5A, FGFR2, FGFR3, CYP26A1, ZEB2, ZBTB16, ISL1, CXCL12, PCDH18, ALDH1A2, CTTNBP2, APP, HOXB2, DKK1, LHX1, CXCR4, UNC5C, RUNX1, TES
GOTERM_BP_5	GO:0008284~positive regulation of cell proliferation	16	7.45E-04	FGFR2, FGFR3, FOSL2, CSF1, ITGB1, PROX1, VASH2, KITL, TGFB2, KDR, ALDH1A2, MYOCD, LHX1, HEY2, PBX1, CD28
GOTERM_BP_5	GO:0030326~embryonic limb morphogenesis	9	8.25E-04	HOXD9, WNT5A, ALDH1A2, DKK1, TBX4, HOXA9, PBX1, FBN2, ZBTB16
GOTERM_BP_5	GO:0035113~embryonic appendage morphogenesis	9	8.25E-04	HOXD9, WNT5A, ALDH1A2, DKK1, TBX4, HOXA9, PBX1, FBN2, ZBTB16
GOTERM_BP_5	GO:0007420~brain development	16	8.65E-04	WNT5A, FGFR2, FGFR3, ZEB2, ISL1, CXCL12, PCDH18, ALDH1A2, CTTNBP2, APP, HOXB2, DKK1, LHX1, CXCR4, UNC5C, TES
KEGG_PATHWAY	mmu05200:Pathways in cancer	16	8.69E-04	WNT5A, FGFR2, FGFR3, RUNX1T1, ZBTB16, KITL, ITGB1, MMP2, COL4A6, TGFB2, CCND1, WNT4, LAMA4, PDGFRA, PDGFRB, RUNX1
**KEGG_PATHWAY**	**mmu05410:Hypertrophic cardiomyopathy (HCM)**	**8**	**8.97E-04**	**TNNC1, MYL3, ITGA4, TPM2, TPM1, ITGB1, TPM4, TGFB2**
GOTERM_BP_5	GO:0045597~positive regulation of cell differentiation	12	9.44E-04	TAL1, FGFR3, ETS1, GATA6, ADAMTS20, CSF1, FOXA1, VWC2, SOCS5, RUNX1, KITL, TGFB2
GOTERM_BP_5	GO:0043009~chordate embryonic development	20	0.001051267	FGFR2, ZEB2, ITGA4, CAPN2, ITGB1, TPM1, HOXD9, HBA-A1, FOXF1A, HOXB2, GATA6, HAND2, HOXB6, PDGFRA, HOXA9, FOXC2, PDGFRB, TGFBR3, PBX1, RUNX1
GOTERM_BP_5	GO:0008354~germ cell migration	4	0.001235274	CXCR4, CXCL12, KITL, ITGB1
GOTERM_BP_5	GO:0048568~embryonic organ development	14	0.001382479	FGFR2, HOXD9, ALDH1A2, FOXF1A, HOXB2, EFNA1, HOXB6, FOXF2, FOXC2, PBX1, COL2A1, ITGA4, RUNX1, PROX1
GOTERM_BP_5	GO:0000904~cell morphogenesis involved in differentiation	13	0.001396355	NRP1, ISL1, PROX1, CXCL12, SLIT2, TGFB2, SLIT3, SEMA5A, APP, WNT4,

				CXCR4, ANTXR1, NFATC1
**KEGG_PATHWAY**	**mmu05414:Dilated cardiomyopathy**	**8**	**0.00153563**	**TNNC1, MYL3, ITGA4, TPM2, TPM1, ITGB1, TPM4, TGFB2**
GOTERM_BP_5	GO:0045639~positive regulation of myeloid cell differentiation	5	0.001540829	TAL1, ETS1, CSF1, RUNX1, KITL
KEGG_PATHWAY	mmu04670:Leukocyte transendothelial migration	9	0.001590912	CLDN7, VAV3, CXCR4, MSN, ITGA4, CXCL12, ITGB1, MMP2, MYL9
KEGG_PATHWAY	mmu04060:Cytokine-cytokine receptor interaction	13	0.001817484	CSF1, KITL, CXCL12, TGFB2, KDR, TNFRSF1A, IFNAR2, IL10RB, CXCR4, PDGFRA, PDGFRB, IFNGR2, GHR
GOTERM_BP_5	GO:0060562~epithelial tube morphogenesis	9	0.001974508	FGFR2, WNT5A, WNT4, FOXF1A, GPC3, CD44, CSF1, PBX1, ZEB2
GOTERM_BP_5	GO:0030097~hemopoiesis	14	0.001987149	ZBTB16, TGFB2, KDR, MLF1, HBA-A1, TAL1, GADD45G, AHSP, HOXA9, TGFBR3, HBB-B1, PBX1, RUNX1, CD28
GOTERM_BP_5	GO:0001667~ameboidal cell migration	6	0.002155228	CER1, CXCR4, ZEB2, ISL1, CXCL12, KITL
GOTERM_BP_5	GO:0030334~regulation of cell migration	8	0.00275548	LAMA4, CXCR4, CSF1, TGFBR3, UNC5C, NEXN, ARAP3, CXCL12
GOTERM_BP_5	GO:0016310~phosphorylation	27	0.003300271	WNT5A, FGFR2, FGFR3, EFNA1, LATS2, TGFB2, CTTNBP2, APP, 2610018G03RIK, SPEG, WNK4, B230120H23RIK, MAP3K8, TES, PTPRE, PKDCC, KDR, MAST4, CCND1, GADD45G, PDGFRA, ROR1, TGFBR3, PDGFRB, NRK, RIPK4, LRRK1
GOTERM_BP_5	GO:0051174~regulation of phosphorus metabolic process	15	0.003619681	FGFR3, VAV3, EFNA1, CSF1, ZEB2, KITL, LATS2, CDKN1C, APP, GADD45G, PDGFRB, NRK, HBB-B1, DNAJC3, GHR
GOTERM_BP_5	GO:0019220~regulation of phosphate metabolic process	15	0.003619681	FGFR3, VAV3, EFNA1, CSF1, ZEB2, KITL, LATS2, CDKN1C, APP, GADD45G, PDGFRB, NRK, HBB-B1, DNAJC3, GHR
GOTERM_MF_5	GO:0004672~protein kinase activity	22	0.003697967	FGFR2, FGFR3, NRP1, PKDCC, LATS2, KDR, MAST4, CTTNBP2, CCND1, 2610018G03RIK, SPEG, WNK4, B230120H23RIK, MAP3K8, ROR1, PDGFRA, PDGFRB, TGFBR3, NRK, RIPK4, LRRK1, TES
GOTERM_BP_5	GO:0030855~epithelial cell differentiation	9	0.00373811	LOR, FGFR2, WNT4, FOXF1A, GATA6, FOXA1, TGFB1I1, PROX1, KDR
GOTERM_BP_5	GO:0060443~mammary gland morphogenesis	5	0.004386141	FGFR2, WNT5A, WNT4, TBX2, CSF1
GOTERM_BP_5	GO:0035051~cardiac cell differentiation	5	0.004386141	GATA6, FHL2, PROX1, ITGB1, TGFB2
GOTERM_BP_5	GO:0000902~cell morphogenesis	15	0.004506602	NRP1, LIPA, ISL1, CXCL12, PROX1, SLIT2, TGFB2, SLIT3, SEMA5A, WNT4, FOXF1A, APP, CXCR4, ANTXR1, NFATC1
GOTERM_BP_5	GO:0051216~cartilage development	7	0.005186785	WNT5A, THRA, FGFR3, BMP1, COL2A1, BMP5, TGFB2
GOTERM_BP_5	GO:0048534~hemopoietic or lymphoid organ development	14	0.005253983	ZBTB16, TGFB2, KDR, MLF1, HBA-A1, TAL1, GADD45G, AHSP, HOXA9, TGFBR3, HBB-B1, PBX1, RUNX1,

				CD28
GOTERM_BP_5	GO:0060284~regulation of cell development	10	0.005402894	NRP1, MYOCD, EFNA1, FOXA1, TGFBR3, VWC2, PBX1, TIMP2, ISL1, PROX1
GOTERM_MF_5	GO:0005540~hyaluronic acid binding	4	0.006134713	HAPLN1, LYVE1, CD44, VCAN
GOTERM_BP_5	GO:0008285~negative regulation of cell proliferation	12	0.006400925	CER1, WNT5A, CTTNBP2, GPC3, FGFR3, TGFBR3, ZBTB16, TIMP2, PROX1, SMARCA2, TES, TGFB2
GOTERM_BP_5	GO:0060419~heart growth	3	0.008176327	TGFBR3, FOXC2, TGFB2
GOTERM_BP_5	GO:0060038~cardiac muscle cell proliferation	3	0.008176327	TGFBR3, FOXC2, TGFB2
GOTERM_BP_5	GO:0001936~regulation of endothelial cell proliferation	4	0.008562683	ALDH1A2, CTTNBP2, VASH2, TES
GOTERM_BP_5	GO:0001701~in utero embryonic development	13	0.008906045	FGFR2, ITGA4, CAPN2, TPM1, ITGB1, HBA-A1, FOXF1A, HAND2, GATA6, PDGFRA, PDGFRB, TGFBR3, RUNX1
GOTERM_BP_5	GO:0051146~striated muscle cell differentiation	7	0.009754117	APP, GATA6, FHL2, CAPN2, PROX1, ITGB1, TMOD1
GOTERM_CC_5	GO:0009897~external side of plasma membrane	11	0.010049966	FRAS1, COL23A1, LRP1, GPC3, CD44, FREM2, GPC6, GYPA, ITGA4, MMP14, CD28
GOTERM_BP_5	GO:0009954~proximal/distal pattern formation	4	0.013946596	HOXD9, ALDH1A2, HOXA9, PBX1
GOTERM_BP_5	GO:0060603~mammary gland duct morphogenesis	4	0.015531972	FGFR2, WNT5A, WNT4, CSF1
GOTERM_BP_5	GO:0060216~definitive hemopoiesis	3	0.01682609	TGFBR3, HOXA9, RUNX1
GOTERM_CC_5	GO:0030054~cell junction	18	0.01703885	CLDN7, INADL, MAGI3, LPP, ARHGAP24, NEXN, CTTNBP2, HMCN1, WNK4, STARD8, SH3KBP1, DSP, DSC2, TGFB1I1, ADD3, PARVA, TES, EPB4.1L5
GOTERM_BP_5	GO:0048666~neuron development	13	0.017057059	NRP1, ISL1, CXCL12, SLIT2, TGFB2, FARP2, SLIT3, HOXD9, CDKN1C, SEMA5A, APP, CXCR4, RUNX1
GOTERM_BP_5	GO:0035136~forelimb morphogenesis	4	0.017214682	HOXD9, ALDH1A2, HOXA9, ZBTB16
GOTERM_BP_5	GO:0043549~regulation of kinase activity	10	0.017275653	APP, VAV3, EFNA1, CSF1, GADD45G, NRK, ZEB2, DNAJC3, KITL, LATS2
GOTERM_BP_5	GO:0051329~interphase of mitotic cell cycle	5	0.017625708	CCND1, APP, ITGB1, LATS2, NFATC1
KEGG_PATHWAY	mmu04672:Intestinal immune network for IgA production	5	0.017698049	CXCR4, ITGA4, CXCL12, CD28, TGFB2
GOTERM_BP_5	GO:0045664~regulation of neuron differentiation	7	0.018187011	NRP1, EFNA1, FOXA1, VWC2, PBX1, TIMP2, ISL1
GOTERM_BP_5	GO:0045449~regulation of transcription	60	0.018295266	MEF2A, THRA, FOSL2, TBX20, ZEB2, HOXC8, APP, FLI1, MYOCD, SMARCD3, GATA6, HEY2, FOXF2, LRRFIP1, TWIST2, EGR3, RUNX1T1, ZFP467, DMRT2, PROX1, MXD4, HOXD9, IFNAR2, HOXD8, NAB1, FOXC2, SMARCA2, ZFP711, CRTC3, CREM, FHL2, SOX7, ELK3, ZBTB16, ZFP618, TNFRSF1A, TAL1, HAND2,

				LHX1, HOXA9, RUNX1, NFATC1, IKZF2, TBX2, FOXA1, TBX4, ZFP672, ISL1, SNAI2, CDKN1C, MSL3, FOXF1A, HOXB2, ETS1, HOXB6, BNC1, TGFBR3, HIVEP2, PBX1, ZBTB1
GOTERM_BP_5	GO:0001894~tissue homeostasis	5	0.018859151	LIPA, CSF1, PDGFRB, COL2A1, KDR
GOTERM_BP_5	GO:0007409~axonogenesis	9	0.01888318	SEMA5A, APP, NRP1, CXCR4, ISL1, CXCL12, SLIT2, SLIT3, TGFB2
GOTERM_BP_5	GO:0055007~cardiac muscle cell differentiation	4	0.018995201	GATA6, FHL2, PROX1, ITGB1
GOTERM_BP_5	GO:0000082~G1/S transition of mitotic cell cycle	4	0.018995201	CCND1, ITGB1, LATS2, NFATC1
GOTERM_BP_5	GO:0010604~positive regulation of macromolecule metabolic process	22	0.019874127	MEF2A, THRA, IKZF2, FGFR3, FOXA1, TBX20, ISL1, KITL, HOXD9, TNFRSF1A, APP, GPC3, MYOCD, SMARCD3, ETS1, HAND2, GATA6, FOXC2, PBX1, RUNX1, CD28, GHR
GOTERM_BP_5	GO:0034101~erythrocyte homeostasis	5	0.020144806	HBA-A1, TAL1, HOXB6, AHSP, TGFBR3
GOTERM_BP_5	GO:0051325~interphase	5	0.020144806	CCND1, APP, ITGB1, LATS2, NFATC1
GOTERM_BP_5	GO:0060343~trabecula formation	3	0.020290716	TGFBR3, FHL2, MMP2
GOTERM_BP_5	GO:0006355~regulation of transcription, DNA-dependent	42	0.022463973	MEF2A, FOSL2, THRA, CREM, TBX20, FHL2, ZEB2, SOX7, ZBTB16, ELK3, TNFRSF1A, TAL1, APP, HOXC8, FLI1, MYOCD, HAND2, GATA6, LHX1, HEY2, FOXF2, HOXA9, RUNX1, TWIST2, NFATC1, IKZF2, TBX2, FOXA1, TBX4, RUNX1T1, DMRT2, ISL1, HOXD9, CDKN1C, IFNAR2, FOXF1A, HOXD8, HOXB2, ETS1, HOXB6, FOXC2, PBX1
GOTERM_BP_5	GO:0045646~regulation of erythrocyte differentiation	3	0.024024408	TAL1, LMO2, ETS1
GOTERM_BP_5	GO:0060420~regulation of heart growth	3	0.024024408	FGFR2, HEY2, PROX1
GOTERM_BP_5	GO:0006935~chemotaxis	7	0.02434283	LSP1, ECSCR, CMTM8, CXCL12, CMTM3, SLIT2, TGFB2
GOTERM_BP_5	GO:0019219~regulation of nucleobase, nucleoside, nucleotide and nucleic acid metabolic process	62	0.024866178	MEF2A, THRA, FOSL2, TBX20, ZEB2, APP, HOXC8, FLI1, MYOCD, SMARCD3, GATA6, HEY2, FOXF2, LRRFIP1, TWIST2, EGR3, RUNX1T1, ZFP467, DMRT2, PROX1, MXD4, HOXD9, IFNAR2, HOXD8, NAB1, FOXC2, SMARCA2, ZFP711, CRTC3, CREM, FHL2, SOX7, ELK3, ZBTB16, TIMP2, ZFP618, TNFRSF1A, TAL1, HAND2, LHX1, HOXA9, RUNX1, CD28, NFATC1, IKZF2, TBX2, FOXA1, TBX4, ZFP672, ISL1, SNAI2, CDKN1C, MSL3, FOXF1A, HOXB2, ETS1, HOXB6, BNC1, TGFBR3, HIVEP2, PBX1, ZBTB1
KEGG_PATHWAY	mmu04010:MAPK signaling pathway	11	0.025288913	FGFR2, TNFRSF1A, FGFR3, DUSP2, RASGRP3, B230120H23RIK, MAP3K8,

				GADD45G, PDGFRA, PDGFRB, TGFB2
GOTERM_CC_5	GO:0015629~actin cytoskeleton	10	0.025495232	CTTNBP2, ARHGAP6, SH3PXD2A, MYL3, CALD1, TPM2, TPM1, BMF, TPM4, MYL9
GOTERM_CC_5	GO:0005887~integral to plasma membrane	19	0.025605186	PAM, IGDCC4, GYPA, ITGA4, PCDH7, SIRPA, ITGB1, KDR, PCDH18, PROM1, GPC3, GPC6, ROR1, PDGFRA, SDCBP, PDGFRB, SGCE, SCN7A, SLCO3A1
GOTERM_BP_5	GO:0048706~embryonic skeletal system development	6	0.02748282	HOXD9, HOXB2, HOXB6, FOXC2, HOXA9, PBX1
GOTERM_BP_5	GO:0042551~neuron maturation	3	0.028014801	CDKN1C, APP, FARP2
GOTERM_BP_5	GO:0002763~positive regulation of myeloid leukocyte differentiation	3	0.028014801	CSF1, RUNX1, KITL
GOTERM_BP_5	GO:0051252~regulation of RNA metabolic process	42	0.028157094	MEF2A, FOSL2, THRA, CREM, TBX20, FHL2, ZEB2, SOX7, ZBTB16, ELK3, TNFRSF1A, TAL1, APP, HOXC8, FLI1, MYOCD, HAND2, GATA6, LHX1, HEY2, FOXF2, HOXA9, RUNX1, TWIST2, NFATC1, IKZF2, TBX2, FOXA1, TBX4, RUNX1T1, DMRT2, ISL1, HOXD9, CDKN1C, IFNAR2, FOXF1A, HOXD8, HOXB2, ETS1, HOXB6, FOXC2, PBX1
GOTERM_BP_5	GO:0048812~neuron projection morphogenesis	9	0.028283991	SEMA5A, APP, NRP1, CXCR4, ISL1, CXCL12, SLIT2, SLIT3, TGFB2
GOTERM_BP_5	GO:0010468~regulation of gene expression	63	0.031682416	MEF2A, THRA, FOSL2, TBX20, ZEB2, APP, HOXC8, FLI1, MYOCD, SMARCD3, GATA6, HEY2, FOXF2, LRRFIP1, TWIST2, EGR3, RUNX1T1, ZFP467, DMRT2, PROX1, MXD4, HOXD9, IFNAR2, HOXD8, AHSP, NAB1, FOXC2, SMARCA2, ZFP711, CRTC3, AIRN, CREM, FHL2, SOX7, COL2A1, ELK3, ZBTB16, ZFP618, TNFRSF1A, TAL1, HAND2, LHX1, HOXA9, RUNX1, NFATC1, IKZF2, TBX2, FOXA1, TBX4, ZFP672, ISL1, SNAI2, CDKN1C, MSL3, FOXF1A, HOXB2, ETS1, HOXB6, BNC1, TGFBR3, HIVEP2, PBX1, ZBTB1
GOTERM_BP_5	GO:0045787~positive regulation of cell cycle	4	0.031739606	APP, PROX1, CD28, TGFB2
GOTERM_BP_5	GO:0048566~embryonic gut development	3	0.032249922	ALDH1A2, FOXF1A, FOXF2
GOTERM_BP_5	GO:0045935~positive regulation of nucleobase, nucleoside, nucleotide and nucleic acid metabolic process	18	0.033035638	MEF2A, THRA, IKZF2, FOXA1, TBX20, ISL1, HOXD9, TNFRSF1A, APP, MYOCD, ETS1, HAND2, GATA6, SMARCD3, FOXC2, PBX1, RUNX1, CD28
GOTERM_BP_5	GO:0048667~cell morphogenesis involved in neuron differentiation	9	0.033572856	SEMA5A, APP, NRP1, CXCR4, ISL1, CXCL12, SLIT2, SLIT3, TGFB2
GOTERM_CC_5	GO:0019898~extrinsic to membrane	17	0.034213009	MAGI3, INADL, CAP2, CYP26A1, FARP2, SH3GLB1, GNG10, SH3KBP1,

				SDCBP, PON2, RIPK4, MSN, ARAP3, PHLDB2, GRB14, EPB4.1L5, GHR
GOTERM_BP_5	GO:0042981~regulation of apoptosis	19	0.03447573	CFLAR, FGFR3, ADAMTS20, DMRT2, COL2A1, ZBTB16, SNAI2, KITL, TGFB2, IKBIP, CASP6, SERPINB9, 2610018G03RIK, SH3GLB1, B230120H23RIK, FOXC2, PPP1R13B, BMF, CD28
GOTERM_BP_5	GO:0045941~positive regulation of transcription	17	0.034857772	MEF2A, THRA, IKZF2, FOXA1, TBX20, ISL1, HOXD9, TNFRSF1A, APP, MYOCD, ETS1, HAND2, GATA6, SMARCD3, FOXC2, PBX1, RUNX1
GOTERM_CC_5	GO:0031226~intrinsic to plasma membrane	19	0.035727486	PAM, IGDCC4, GYPA, ITGA4, PCDH7, SIRPA, ITGB1, KDR, PCDH18, PROM1, GPC3, GPC6, ROR1, PDGFRA, SDCBP, PDGFRB, SGCE, SCN7A, SLCO3A1
GOTERM_BP_5	GO:0055001~muscle cell development	5	0.035968785	APP, FHL2, PROX1, ITGB1, TMOD1
GOTERM_BP_5	GO:0010927~cellular component assembly involved in morphogenesis	4	0.036767892	PMP22, PROX1, ITGB1, TMOD1
GOTERM_BP_5	GO:0043067~regulation of programmed cell death	19	0.038311003	CFLAR, FGFR3, ADAMTS20, DMRT2, COL2A1, ZBTB16, SNAI2, KITL, TGFB2, IKBIP, CASP6, SERPINB9, 2610018G03RIK, SH3GLB1, B230120H23RIK, FOXC2, PPP1R13B, BMF, CD28
GOTERM_BP_5	GO:0050678~regulation of epithelial cell proliferation	5	0.039795937	FGFR2, WNT5A, FGFR3, TGFBR3, KDR
GOTERM_CC_5	GO:0005862~muscle thin filament tropomyosin	2	0.040566792	TPM2, TPM1
GOTERM_BP_5	GO:0031325~positive regulation of cellular metabolic process	21	0.041347555	MEF2A, THRA, IKZF2, FGFR3, FOXA1, TBX20, ISL1, KITL, HOXD9, TNFRSF1A, APP, MYOCD, SMARCD3, ETS1, HAND2, GATA6, FOXC2, PBX1, RUNX1, CD28, GHR
GOTERM_BP_5	GO:0001502~cartilage condensation	3	0.041408331	THRA, COL2A1, TGFB2
GOTERM_BP_5	GO:0043087~regulation of GTPase activity	6	0.041854387	TBC1D2B, ARHGAP6, VAV3, RASGRP3, RABGAP1L, ARAP3
GOTERM_BP_5	GO:0051173~positive regulation of nitrogen compound metabolic process	18	0.042301007	MEF2A, THRA, IKZF2, FOXA1, TBX20, ISL1, HOXD9, TNFRSF1A, APP, MYOCD, ETS1, HAND2, GATA6, SMARCD3, FOXC2, PBX1, RUNX1, CD28
GOTERM_BP_5	GO:0016481~negative regulation of transcription	14	0.042551701	THRA, TBX2, TBX20, FOXA1, ZEB2, ZBTB16, MXD4, CDKN1C, HOXC8, HOXD8, MYOCD, NAB1, FOXF2, TWIST2
GOTERM_BP_5	GO:0010628~positive regulation of gene expression	17	0.042702298	MEF2A, THRA, IKZF2, FOXA1, TBX20, ISL1, HOXD9, TNFRSF1A, APP, MYOCD, ETS1, HAND2, GATA6, SMARCD3, FOXC2, PBX1, RUNX1
GOTERM_BP_5	GO:0010556~regulation of macromolecule biosynthetic process	61	0.043311151	MEF2A, THRA, FOSL2, TBX20, ZEB2, HOXC8, APP, FLI1, MYOCD, SMARCD3, GATA6, HEY2, FOXF2,

				LRRFIP1, TWIST2, EGR3, RUNX1T1, ZFP467, DMRT2, PROX1, MXD4, HOXD9, IFNAR2, HOXD8, NAB1, FOXC2, SMARCA2, ZFP711, CRTC3, CREM, FHL2, SOX7, ELK3, ZBTB16, ZFP618, TNFRSF1A, TAL1, HAND2, LHX1, HOXA9, RUNX1, CD28, NFATC1, IKZF2, TBX2, FOXA1, TBX4, ZFP672, ISL1, SNAI2, CDKN1C, MSL3, FOXF1A, HOXB2, ETS1, HOXB6, BNC1, TGFBR3, HIVEP2, PBX1, ZBTB1
GOTERM_BP_5	GO:0010557~positive regulation of macromolecule biosynthetic process	18	0.04460825	MEF2A, THRA, IKZF2, FOXA1, TBX20, ISL1, HOXD9, TNFRSF1A, APP, MYOCD, ETS1, HAND2, GATA6, SMARCD3, FOXC2, PBX1, RUNX1, CD28
GOTERM_BP_5	GO:0051056~regulation of small GTPase mediated signal transduction	10	0.044846251	TBC1D2B, VAV3, RASGRP3, CSF1, RABGAP1L, AKAP13, ARHGEF17, ARAP3, KITL, FARP2
GOTERM_BP_5	GO:0030111~regulation of Wnt receptor signaling pathway	4	0.04502611	CCND1, LRP1, DKK1, ZEB2
GOTERM_BP_5	GO:0048565~gut development	4	0.04502611	WNT5A, ALDH1A2, FOXF1A, FOXF2
GOTERM_CC_5	GO:0005938~cell cortex	7	0.045159491	HMCN1, CALD1, EXOC6, ADD3, TPM4, ITPR2, TMOD1
GOTERM_BP_5	GO:0045766~positive regulation of angiogenesis	3	0.046309525	ERAP1, RUNX1, VASH2
GOTERM_BP_5	GO:0045893~positive regulation of transcription, DNA-dependent	15	0.047107714	IKZF2, THRA, FOXA1, TBX20, ISL1, HOXD9, TNFRSF1A, APP, MYOCD, ETS1, HAND2, GATA6, FOXC2, PBX1, RUNX1
GOTERM_BP_5	GO:0048562~embryonic organ morphogenesis	8	0.047508566	FGFR2, HOXD9, HOXB2, EFNA1, HOXB6, FOXC2, COL2A1, PROX1
GOTERM_BP_5	GO:0010810~regulation of cell-substrate adhesion	4	0.047966243	FBLN2, CSF1, VWC2, MMP14
KEGG_PATHWAY	mmu04810:Regulation of actin cytoskeleton	9	0.04879881	FGFR2, VAV3, FGFR3, PDGFRA, PDGFRB, MSN, ITGA4, ITGB1, MYL9
GOTERM_BP_5	GO:0051254~positive regulation of RNA metabolic process	15	0.049248193	IKZF2, THRA, FOXA1, TBX20, ISL1, HOXD9, TNFRSF1A, APP, MYOCD, ETS1, HAND2, GATA6, FOXC2, PBX1, RUNX1
**Cluster 5**				
**Category**	**Term**	**Count**	**PValue**	**Genes**
**GOTERM_CC_5**	**GO:0005634~nucleus**	**208**	**2.83E-40**	**KIFC1, NAF1, LEMD1, WDR74, CDCA8, CDCA7, SIN3A, ZFP473, CDCA2, NUP37, RPL12, CCNA2, CDCA5, GTPBP4, RCOR2, TAF4B, LIG1, 4632434I11RIK, ESPL1, ESCO2, NCAPD2, RFC5, UHRF1, AQR, RCC2, MTF2, PRDM5, MYBBP1A, NUP43, SDAD1, NEK2, NOC3L, IGF2BP1, CHEK1, CHEK2, MYBL1, RCC1, OBFC2A, FOXH1, PIR, FBXO5, NAT10, DNMT3B, UTP23, RANBP17, UTF1, NDC80, ATR, RAD54L, ATM,**

				**NOLC1, SUPT16H, UTP14A, PARP1, CHAF1B, RPP40, UTP18, TAF1D, TCOF1, CDT1, CCNE1, FANCM, RRP1B, FANCI, NUBP2, SEH1L, EXOSC5, CCNF, TLE4, TTF2, RAD51, ZSCAN10, FANCD2, NSD1, NHP2, WDR43, USP7, UNG, PML, POLA1, NUFIP1, BC066028, GM13212, C79407, NPAT, THAP1, ETV5, BAZ2A, RBM25, POLR3G, NUCKS1, GMNN, DLGAP5, NASP, ILF3, PNLDC1, ATMIN, CCNB1, ZFP106, RPS6KA2, ZFP7, KIF20B, CHTF18, USP48, BARD1, 5730507C01RIK, XPO5, XPO4, DBF4, KNTC1, MCM10, SETX, MUTYH, TDGF1, DDX21, DNAJC2, GNL3, NUP133, SNRPN, DTL, SGOL2, SGOL1, GABPA, MND1, SIX4, DDX39, TIMELESS, MGA, RAD18, DSCC1, HMGB2, BLM, MAPKAPK3, ANLN, BEX4, LIN28A, ZFP568, ZFP566, TFDP2, DYRK3, PALB2, HELLS, GINS1, TRP53, ZMYM1, PIF1, WDR5, TAF7, ARID3B, CASC5, BRCA2, ATAD5, ATF7IP2, BRCA1, NRF1, SALL4, POLD1, SALL1, HIST1H3A, TSX, HDAC6, LYAR, CAD, KIF2C, MCM8, MCM7, DKC1, PCGF6, CDK12, RTEL1, PHC1, FTSJ3, KIF11, NOL9, NOL8, MCM2, HLTF, MCM3, HMGA1, MCM5, APITD1, RIF1, NCOA3, PKM2, NSL1, IPO5, BUB1B, RANGRF, TXNRD1, MATR3, ING5, HAT1, CALCA, HIC2, HMGXB4, GEMIN5, TERF1, EXO1, RAD51AP1, L3MBTL2, MSH2, CENPP, CENPF, MLF1IP, SMC2, GSG2, CENPH, SRFBP1**
GOTERM_BP_5	GO:0000279~M phase	46	1.66E-25	KIFC1, NEK2, USP9X, HAUS1, KNTC1, ANLN, RCC1, C79407, CDCA8, SEH1L, F630043A04RIK, CDCA2, FBXO5, NUP37, SKA1, ZWILCH, CCNA2, CDCA5, HELLS, ERCC6L, TERF1, EXO1, CCNB1IP1, KIF11, DLGAP5, SGOL2, SGOL1, CCNF, CENPF, MND1, BRCA2, NDC80, ESPL1, SMC2, NCAPD2, CENPH, RAD51, CCNB1, RCC2, TIMELESS, FANCD2, RPS6KA2, NSL1, KIF20B, BUB1B, NUP43
GOTERM_BP_5	GO:0000087~M phase of mitotic cell cycle	38	6.19E-24	KIFC1, NEK2, USP9X, HAUS1, KNTC1, ANLN, RCC1, C79407, CDCA8, SEH1L, F630043A04RIK, CDCA2, FBXO5, NUP37, SKA1, ZWILCH, CCNA2, CDCA5, HELLS, TERF1, ERCC6L, KIF11, DLGAP5, SGOL1, CCNF, CENPF, NDC80, SMC2, NCAPD2, CENPH, CCNB1, RCC2, TIMELESS, RPS6KA2,

				NSL1, KIF20B, BUB1B, NUP43
GOTERM_CC_5	GO:0015630~microtubule cytoskeleton	23	3.85E-04	KIFC1, KIF11, NEK2, DLGAP5, HAUS1, KNTC1, CENPF, GTSE1, BRCA1, ATM, HOOK1, CCNB1, KIF2C, SPRY2, CDCA8, RCC2, RPS6KA2, MCPH1, F630043A04RIK, KIF20B, FBXO5, ODF2, SKA1
GOTERM_BP_5	GO:0051323~metaphase	4	3.88E-04	CCNB1IP1, RPS6KA2, FBXO5, NDC80
GOTERM_BP_5	GO:0010332~response to gamma radiation	5	5.06E-04	TRP53, PML, BRCA2, CHEK2, PNP
GOTERM_CC_5	GO:0045120~pronucleus	5	5.41E-04	BLM, RIF1, CENPF, CCNA2, NCAPD2
KEGG_PATHWAY	mmu00240:Pyrimidine metabolism	9	5.54E-04	POLR3G, RRM2, POLD1, POLA1, UPP1, TXNRD1, CAD, UCK2, PNP
GOTERM_BP_5	GO:0006275~regulation of DNA replication	5	6.39E-04	TRP53, GTPBP4, GMNN, BRCA2, CDT1
KEGG_PATHWAY	mmu03430:Mismatch repair	5	7.93E-04	RFC5, EXO1, MSH2, POLD1, LIG1
GOTERM_BP_5	GO:0051329~interphase of mitotic cell cycle	7	8.36E-04	SIN3A, DBF4, POLA1, CENPF, CHEK1, DNAJC2, CDCA5
**GOTERM_BP_5**	**GO:0034470~ncRNA processing**	**12**	**0.001023385**	**UTP23, NAF1, RRP1B, DKC1, UTP18, EXOSC5, POP1, LIN28A, UTP14A, NHP2, FTSJ3, RPP40**
GOTERM_BP_5	GO:0051325~interphase	7	0.001035784	SIN3A, DBF4, POLA1, CENPF, CHEK1, DNAJC2, CDCA5
GOTERM_BP_5	GO:0043414~biopolymer methylation	8	0.001165213	PRDM5, ILF3, ETF1, DNMT3B, NSD1, BAZ2A, FTSJ3, HELLS
GOTERM_CC_5	GO:0030529~ribonucleoprotein complex	22	0.001315705	NAF1, SNRPN, NUFIP1, LIN28A, TTF2, BRCA1, AQR, MRPS18B, MRPL15, DKC1, NOLC1, RRP1B, RPS6KA2, RPS4Y2, WDR89, GM5879, RPL12, UTP14A, NHP2, RPP40, GEMIN5, RPS24
GOTERM_CC_5	GO:0005657~replication fork	5	0.001694728	RFC5, TRP53, BLM, POLA1, CHEK1
GOTERM_MF_5	GO:0000287~magnesium ion binding	19	0.001708776	AMHR2, EXO1, ALPL, NUDT17, PFKL, NEK2, POLG, LIG1, GM5081, PFKP, CHEK2, GSG2, ACVR2B, RPS6KA2, PKM2, DYRK3, ENOPH1, B4GALT6, ENO1, PRPS1
KEGG_PATHWAY	mmu03440:Homologous recombination	5	0.001764033	BLM, POLD1, BRCA2, RAD54L, RAD51
**GOTERM_BP_5**	**GO:0034660~ncRNA metabolic process**	**13**	**0.002398898**	**UTP23, NAF1, UTP18, EXOSC5, DARS2, LIN28A, DKC1, RRP1B, POP1, UTP14A, NHP2, FTSJ3, RPP40**
KEGG_PATHWAY	mmu00250:Alanine, aspartate and glutamate metabolism	5	0.002632381	GLS2, GOT1, GFPT2, CAD, PPAT
GOTERM_CC_5	GO:0000792~heterochromatin	6	0.005023992	RAD18, ATR, DNMT3B, BAZ2A, PHC1, HELLS
**GOTERM_BP_5**	**GO:0040029~regulation of gene expression, epigenetic**	**7**	**0.005256034**	**XIST, HAT1, LIN28A, DNMT3B, BAZ2A, BRCA1, HELLS**
GOTERM_BP_5	GO:0016481~negative regulation of transcription	18	0.005328265	TRP53, RCOR2, NODAL, GABPA, PML, CENPF, HAT1, TLE4, FOXH1, SIN3A, SALL4, TIMELESS, PCGF6, NSD1, BAZ2A, MYBBP1A, DNMT3B, HELLS
KEGG_PATHWAY	mmu00051:Fructose and mannose metabolism	5	0.005708222	PFKL, AKR1B3, HK2, PFKP, PMM1

GOTERM_CC_5	GO:0044430~cytoskeletal part	29	0.006272299	KIFC1, ENAH, NEK2, HAUS1, KNTC1, ANLN, GTSE1, HOOK1, KIF2C, SPRY2, CDCA8, F630043A04RIK, FBXO5, CHRNA7, ODF2, SKA1, KIF11, DLGAP5, CENPF, ACTN3, BRCA1, ATM, CCNB1, MYO10, RCC2, RPS6KA2, MCPH1, KIF20B, SPNA1
GOTERM_BP_5	**GO:0006913~nucleocytoplasmic transport**	8	0.006446672	TRP53, NUP133, RANBP17, XPO5, IPO5, PML, RANGRF, MYBBP1A
GOTERM_BP_5	GO:0010564~regulation of cell cycle process	7	0.006447793	DBF4, PML, FBXO5, BRCA2, CENPF, ATM, CDT1
GOTERM_BP_5	GO:0019320~hexose catabolic process	6	0.006558643	PFKL, PKM2, HK2, PFKP, PDHA1, ENO1
GOTERM_CC_5	GO:0005635~nuclear envelope	10	0.006570017	NUP133, RANBP17, SEH1L, IPO5, TFDP2, POLA1, CENPF, NUP37, LEMD1, NUP43
GOTERM_BP_5	GO:0051169~nuclear transport	8	0.007200665	TRP53, NUP133, RANBP17, XPO5, IPO5, PML, RANGRF, MYBBP1A
GOTERM_BP_5	GO:0051310~metaphase plate congression	3	0.007373944	SEH1L, CENPF, CDCA5
KEGG_PATHWAY	mmu03410:Base excision repair	5	0.007550306	MUTYH, UNG, POLD1, LIG1, PARP1
GOTERM_BP_5	GO:0046365~monosaccharide catabolic process	6	0.007691752	PFKL, PKM2, HK2, PFKP, PDHA1, ENO1
GOTERM_BP_5	GO:0009411~response to UV	5	0.00806061	TRP53, MSH2, PML, RAD18, BRCA2
GOTERM_BP_5	GO:0007127~meiosis I	5	0.00806061	CCNB1IP1, FANCD2, FBXO5, ESPL1, RAD51
**GOTERM_BP_5**	**GO:0007281~germ cell development**	**8**	**0.008451608**	**CCNB1IP1, CALCA, HOOK1, HMGB2, MSH2, RPS6KA2, FBXO5, BRCA2**
GOTERM_BP_5	GO:0001701~in utero embryonic development	14	0.008618963	TRP53, CCNB1IP1, GINS1, MSH2, NODAL, NASP, GABPA, BRCA2, CCNB1, SIN3A, SALL4, RRN3, TDGF1, ENO1
**GOTERM_CC_5**	**GO:0005643~nuclear pore**	**6**	**0.00929673**	**NUP133, RANBP17, SEH1L, IPO5, NUP37, NUP43**
**GOTERM_BP_5**	**GO:0016458~gene silencing**	**6**	**0.009636354**	**XPO5, HAT1, LIN28A, DNMT3B, BAZ2A, HELLS**
GOTERM_CC_5	GO:0000785~chromatin	10	0.010532312	HIST1H3A, POLA1, RAD18, NUFIP1, ATR, DNMT3B, HMGA1, BAZ2A, PHC1, HELLS
**KEGG_PATHWAY**	**mmu00010:Glycolysis / Gluconeogenesis**	**6**	**0.010547776**	**PFKL, PKM2, HK2, PFKP, PDHA1, ENO1**
GOTERM_BP_5	GO:0044275~cellular carbohydrate catabolic process	6	0.011894935	PFKL, PKM2, HK2, PFKP, PDHA1, ENO1
**GOTERM_CC_5**	**GO:0031080~Nup107-160 complex**	**3**	**0.012263963**	**NUP133, SEH1L, NUP43**
GOTERM_CC_5	GO:0016363~nuclear matrix	5	0.013124767	POLA1, PML, NUFIP1, CENPF, MATR3
GOTERM_BP_5	GO:0045814~negative regulation of gene expression, epigenetic	4	0.013254005	HAT1, DNMT3B, BAZ2A, HELLS
**GOTERM_BP_5**	**GO:0000154~rRNA modification**	**3**	**0.01335401**	**DKC1, NHP2, FTSJ3**
GOTERM_BP_5	GO:0051303~establishment of chromosome localization	3	0.01335401	SEH1L, CENPF, CDCA5
GOTERM_CC_5	GO:0005874~microtubule	12	0.016871185	HOOK1, SPRY2, KIF2C, KIFC1, KIF11, RCC2, F630043A04RIK, HAUS1, KIF20B, ODF2, SKA1, GTSE1

**GOTERM_BP_5**	**GO:0043009~chordate embryonic development**	**18**	**0.016881011**	**TRP53, CCNB1IP1, GINS1, ENAH, MSH2, NODAL, NASP, GABPA, BRCA2, SIX4, BRCA1, ATM, CCNB1, SIN3A, SALL4, RRN3, TDGF1, ENO1**
GOTERM_BP_5	GO:0046112~nucleobase biosynthetic process	3	0.016912105	CAD, PPAT, GART
**GOTERM_CC_5**	**GO:0046930~pore complex**	**6**	**0.021819322**	**NUP133, RANBP17, SEH1L, IPO5, NUP37, NUP43**
**GOTERM_BP_5**	**GO:0007498~mesoderm development**	**5**	**0.045896924**	**FOXH1, NUP133, ACVR2B, NODAL, TXNRD1**
GOTERM_CC_5	GO:0005732~small nucleolar ribonucleoprotein complex	3	0.046856184	NAF1, RPL12, NHP2
**Cluster 6**				
**Category**	**Term**	**Count**	**PValue**	**Genes**
**KEGG_PATHWAY**	**mmu04610:Complement and coagulation cascades**	**31**	**8.09E-21**	**C3AR1, C9, MASP1, C3, C1S, C1QC, F13B, FGG, SERPINA1B, FGA, FGB, SERPINA1A, KLKB1, CFH, SERPINC1, CFI, C2, F11, KNG1, F12, F10, HC, SERPING1, PLG, C8A, C1QA, C1QB, SERPINF2, F2, SERPIND1, CPB2**
**GOTERM_BP_5**	**GO:0002526~acute inflammatory response**	**26**	**2.41E-17**	**PTGER3, C9, MASP1, HC, C3, CRP, SAA3, SERPING1, TLR4, C1S, FCGR1, C1QC, TRF, STAT3, AHSG, C8A, C1QA, C1QB, SERPINA1B, SERPINF2, SERPINA1A, F2, CFH, CFI, C2, LBP**
**GOTERM_BP_5**	**GO:0007596~blood coagulation**	**22**	**1.83E-14**	**KNG1, F11, F12, F10, C9, PLEK, MST1, SERPING1, PLG, F13B, FGG, PROCR, FGA, FGB, KLKB1, PROZ, F2, SERPINC1, APOH, SERPIND1, TFPI2, PAPSS2**
GOTERM_BP_5	GO:0006956~complement activation	13	2.06E-09	C1QA, C8A, C1QB, C9, MASP1, HC, C3, CFH, SERPING1, CFI, C1S, C2, C1QC
GOTERM_BP_5	GO:0019724~B cell mediated immunity	16	5.99E-09	C9, HC, C3, IGH-6, SERPING1, C1S, C1QC, FCGR1, C8A, C1QA, C1QB, FCGR2B, FCER1G, FAS, C2, CFI
GOTERM_BP_5	GO:0030193~regulation of blood coagulation	9	6.04E-09	F11, F12, PLEK, KLKB1, APOH, TLR4, TMPRSS6, CPB2, PLG
**GOTERM_CC_5**	**GO:0043292~contractile fiber**	**18**	**1.85E-08**	**ACTA2, PDLIM5, MYBPC3, LDB3, MYOZ2, ANKRD1, MYH6, TNNI3, TTN, CSRP3, VCL, KRT19, ABCC9, PGM5, KCNJ8, SPNB2, ITGB1BP2, MYOM1**
GOTERM_CC_5	GO:0044449~contractile fiber part	17	2.73E-08	ACTA2, PDLIM5, MYBPC3, LDB3, MYOZ2, ANKRD1, MYH6, TNNI3, TTN, CSRP3, VCL, KRT19, ABCC9, PGM5, SPNB2, ITGB1BP2, MYOM1
GOTERM_BP_5	GO:0051604~protein maturation	18	4.08E-08	C9, MASP1, HC, C3, SERPING1, C1S, MMP14, C1QC, CPZ, AMBP, C8A, C1QA, C1QB, CFH, CFI, C2, CASP1, ADAMTS2
GOTERM_BP_5	GO:0006958~complement activation, classical pathway	11	4.69E-08	C1QA, C8A, C1QB, C9, HC, C3, SERPING1, CFI, C1S, C2, C1QC
**GOTERM_BP_5**	**GO:0002253~activation of**	**17**	**4.97E-08**	**C9, MASP1, HC, C3, IGH-6, TLR4,**

	**immune response**			**SERPING1, C1S, C1QC, C8A, C1QA, C1QB, CFH, FCER1G, CLEC7A, C2, CFI**
GOTERM_BP_5	GO:0002449~lymphocyte mediated immunity	16	5.72E-08	C9, HC, C3, IGH-6, SERPING1, C1S, C1QC, FCGR1, C8A, C1QA, C1QB, FCGR2B, FCER1G, FAS, C2, CFI
GOTERM_CC_5	GO:0030016~myofibril	17	6.28E-08	PDLIM5, MYBPC3, LDB3, MYOZ2, ANKRD1, MYH6, TNNI3, TTN, CSRP3, VCL, KRT19, ABCC9, PGM5, KCNJ8, SPNB2, ITGB1BP2, MYOM1
GOTERM_BP_5	GO:0050778~positive regulation of immune response	21	6.82E-08	C9, MASP1, HC, C3, IGH-6, SERPING1, TLR4, C1S, C1QC, FCGR1, C8A, C1QA, C1QB, HPX, CFH, FCER1G, CFI, LBP, CLEC7A, C2, CD28
GOTERM_BP_5	GO:0050727~regulation of inflammatory response	14	7.27E-08	PRKCA, C3, SERPING1, FCGR1, AHSG, FCGR2B, CFH, TGM2, FABP4, FCER1G, BCL6, LBP, NT5E, CD28
GOTERM_BP_5	GO:0016485~protein processing	17	9.69E-08	C9, MASP1, HC, C3, SERPING1, C1S, MMP14, C1QC, CPZ, C8A, C1QA, C1QB, CFH, CFI, C2, CASP1, ADAMTS2
GOTERM_BP_5	GO:0031347~regulation of defense response	18	1.20E-07	PRKCA, C3, MYO1F, TLR4, SERPING1, FCGR1, AHSG, FCGR2B, HPX, CFH, TGM2, FCER1G, FABP4, BCL6, LBP, CLEC7A, NT5E, CD28
GOTERM_CC_5	GO:0030017~sarcomere	15	4.47E-07	PDLIM5, MYBPC3, LDB3, MYOZ2, ANKRD1, MYH6, TNNI3, TTN, CSRP3, ABCC9, KRT19, PGM5, SPNB2, ITGB1BP2, MYOM1
**GOTERM_BP_5**	**GO:0060429~epithelium development**	**28**	**1.35E-06**	**KRT6A, ONECUT1, PGF, ELF5, ONECUT2, PTGS1, HOXD13, EHF, NR3C1, JAG1, GREM1, WT1, ALDH1A1, TCF21, WNT4, XBP1, PPL, CASP8, TRP63, SEMA3C, EGFR, CEBPB, SCEL, FZD6, CCL11, HHEX, SPRR1A, IRF6**
GOTERM_BP_5	GO:0006935~chemotaxis	17	1.45E-06	PRKCA, C3AR1, CCL2, CREB3, CCR1, CCL9, ITGA1, ITGB2, CCL7, CCL6, CXCL10, CCL11, CXCL15, CX3CR1, FCER1G, LBP, LECT2
GOTERM_CC_5	GO:0005911~cell-cell junction	21	1.79E-06	CLDN8, OCLN, CLDN5, PCDH12, CXADR, GJA4, CLDN23, GJB1, CDH5, VCL, GJA3, GJB2, PGM5, MARVELD2, PPL, CLDN1, DSC2, ESAM, PERP, AHNAK, SYNPO
GOTERM_MF_5	GO:0004867~serine-type endopeptidase inhibitor activity	17	4.24E-06	SERPINA10, PZP, CD109, SERPING1, AMBP, SERPINA1B, SERPINA6, SERPINA1A, SERPINF2, SERPINA3G, SERPINA1C, SERPINC1, ITIH4, ITIH2, SERPIND1, ITIH3, TFPI2
KEGG_PATHWAY	mmu04060:Cytokine-cytokine receptor interaction	30	5.12E-06	IL1R1, CCL2, OSMR, CCR1, CCL9, TGFB3, CCL7, CCL6, CXCL10, CLCF1, IL1RAP, IL11RA2, CSF2RB, IL2RG, PDGFC, FAS, IL13RA1, FIGF, IFNGR1, CSF1R, EGFR, LTBR, MET, LIFR, CCL11, INHBA, PPBP, CCR5, CXCL15, CX3CR1

GOTERM_BP_5	GO:0010810~regulation of cell-substrate adhesion	10	8.68E-06	SMOC2, ONECUT1, ONECUT2, CCDC80, BCL6, VTN, COL1A1, MMP14, PIK3R1, ABI3BP
GOTERM_BP_5	GO:0050729~positive regulation of inflammatory response	8	1.41E-05	PRKCA, C3, TGM2, FCER1G, FABP4, LBP, FCGR1, CD28
GOTERM_BP_5	GO:0002673~regulation of acute inflammatory response	7	2.54E-05	FCGR2B, C3, CFH, FCER1G, SERPING1, LBP, FCGR1
GOTERM_BP_5	GO:0001944~vasculature development	24	2.98E-05	MEF2C, CAV1, PGF, MMP19, ENPEP, TNNI3, MMP14, GJA4, WT1, PLG, CDH5, EDNRA, TCF21, MEOX2, DLL4, CASP8, TGM2, SEMA3C, SOX18, COL1A1, LOX, ANGPTL3, FIGF, TNFAIP2
GOTERM_MF_5	GO:0004175~endopeptidase activity	34	3.03E-05	MASP1, PAMR1, MST1, C1S, TTN, KLKB1, PROZ, CASP8, CFI, C2, ADAM8, CASP1, HGFAC, F11, F12, CYM, F10, MMP19, CTSS, MMP14, CELA1, TMPRSS6, TINAG, PLG, PGM5, F2, CASP12, CTSE, TMPRSS11A, CTSB, ADAM12, ADAMTS2, ADAMTS5, ADAMDEC1
GOTERM_BP_5	GO:0030855~epithelial cell differentiation	16	3.05E-05	KRT6A, CEBPB, ONECUT1, ELF5, PTGS1, ONECUT2, EHF, WT1, SCEL, TCF21, WNT4, IRF6, SPRR1A, XBP1, PPL, TRP63
GOTERM_BP_5	GO:0030595~leukocyte chemotaxis	8	3.31E-05	PRKCA, CCR1, CXCL15, CX3CR1, ITGA1, FCER1G, ITGB2, LBP
GOTERM_BP_5	GO:0060326~cell chemotaxis	8	3.31E-05	PRKCA, CCR1, CXCL15, CX3CR1, ITGA1, FCER1G, ITGB2, LBP
GOTERM_CC_5	GO:0016327~apicolateral plasma membrane	14	4.58E-05	CLDN8, OCLN, CLDN5, CXADR, CDH5, CLDN23, FZD6, MARVELD2, PPL, CLDN1, DSC2, ESAM, PERP, SYNPO
GOTERM_MF_5	GO:0008201~heparin binding	13	4.59E-05	F11, LPL, CCDC80, VTN, ABI3BP, CCL7, SMOC2, CRISPLD2, SERPINC1, APOH, CFH, SERPIND1, THBS2
GOTERM_BP_5	GO:0001568~blood vessel development	23	6.05E-05	MEF2C, CAV1, PGF, MMP19, ENPEP, TNNI3, MMP14, GJA4, WT1, PLG, CDH5, EDNRA, MEOX2, DLL4, CASP8, TGM2, SEMA3C, SOX18, COL1A1, LOX, ANGPTL3, FIGF, TNFAIP2
GOTERM_BP_5	GO:0006937~regulation of muscle contraction	9	8.23E-05	ANXA6, PRKCA, CAV1, ATP2A2, NCF1, GUCY1A3, TNNI3, CASQ1, DMPK
GOTERM_BP_5	GO:0019752~carboxylic acid metabolic process	35	1.00E-04	THA1, KYNU, ECH1, EHHADH, ALDOB, PTGS1, PAH, ALDH1L2, 1700113I22RIK, ALDH1A1, ARG1, APOA2, ATP8B1, ALDH1A7, HPGDS, TMEM195, ACSL5, PLD1, NCF1, OTC, GGH, FBP1, FADS3, FTCD, HGD, CPS1, UGT1A1, CSGALNACT1, ACADVL, BHMT, FABP4, GAMT, UROC1, HPGD, MGST2
GOTERM_BP_5	GO:0050900~leukocyte migration	9	1.17E-04	PRKCA, CD34, CCR1, CXCL15, CX3CR1, ITGA1, FCER1G, ITGB2, LBP
GOTERM_CC_5	GO:0034364~high-density lipoprotein particle	6	1.43E-04	APOA2, APOA1, APOF, SAA3, APOM, GPIHBP1

GOTERM_CC_5	GO:0043296~apical junction complex	13	1.61E-04	CLDN8, OCLN, MARVELD2, PPL, CLDN5, CLDN1, DSC2, ESAM, PERP, CXADR, CLDN23, CDH5, SYNPO
GOTERM_BP_5	GO:0031349~positive regulation of defense response	10	1.67E-04	PRKCA, C3, TGM2, FCER1G, FABP4, TLR4, CLEC7A, LBP, FCGR1, CD28
GOTERM_BP_5	GO:0050819~negative regulation of coagulation	5	1.73E-04	PROCR, KLKB1, TMPRSS6, CPB2, PLG
KEGG_PATHWAY	mmu04670:Leukocyte transendothelial migration	17	1.76E-04	CLDN8, PRKCA, MYL7, OCLN, NCF1, NCF4, CLDN5, CD99, ITGB2, CLDN23, CDH5, VCL, VCAM1, CYBB, CLDN1, ESAM, PIK3R1
GOTERM_MF_5	GO:0008238~exopeptidase activity	11	2.18E-04	AEBP1, CPA2, GGH, ACE2, CPA1, ENPEP, CPB2, DPEP1, TMEM27, CPZ, CPN1
GOTERM_CC_5	GO:0009897~external side of plasma membrane	19	2.42E-04	MMP19, ITGA1, IGH-6, ENPEP, MMP14, FCGR1, ALCAM, CD86, EMR1, FCGR2B, CCR5, CD34, FCER1G, IL2RG, FAS, CLEC7A, CTSB, CD200R4, CD28
GOTERM_BP_5	GO:0006957~complement activation, alternative pathway	5	2.81E-04	C8A, C9, HC, C3, CFH
GOTERM_CC_5	GO:0031674~I band	9	3.92E-04	KRT19, PGM5, PDLIM5, ITGB1BP2, LDB3, MYOZ2, ANKRD1, TTN, CSRP3
GOTERM_BP_5	GO:0032103~positive regulation of response to external stimulus	8	3.96E-04	PRKCA, C3, TGM2, FCER1G, FABP4, LBP, FCGR1, CD28
KEGG_PATHWAY	mmu05322:Systemic lupus erythematosus	15	3.97E-04	HIST1H2BC, C9, HC, C3, FCGR4, C1S, C1QC, FCGR1, C1QA, C8A, C1QB, CD86, FCGR2B, C2, CD28
GOTERM_MF_5	GO:0008009~chemokine activity	8	3.99E-04	CCL11, CCL2, PPBP, CXCL15, CCL9, CCL7, CCL6, CXCL10
GOTERM_BP_5	GO:0042730~fibrinolysis	4	4.25E-04	KLKB1, TMPRSS6, CPB2, PLG
GOTERM_MF_5	GO:0019864~IgG binding	4	4.64E-04	FCGR2B, FCER1G, FCGRT, FCGR1
GOTERM_MF_5	GO:0042379~chemokine receptor binding	8	4.71E-04	CCL11, CCL2, PPBP, CXCL15, CCL9, CCL7, CCL6, CXCL10
GOTERM_MF_5	GO:0008237~metallopeptidase activity	18	5.10E-04	AEBP1, ADAMTSL1, MMP19, ENPEP, MMP14, CPZ, TMEM27, CPN1, ACE2, CPA2, CPA1, ADAM8, ADAM12, CPB2, ADAMTS2, ADAMDEC1, DPEP1, ADAMTS5
GOTERM_MF_5	GO:0004252~serine-type endopeptidase activity	18	5.77E-04	F11, F12, F10, MASP1, PAMR1, MST1, C1S, CELA1, TMPRSS6, PLG, PGM5, KLKB1, PROZ, F2, TMPRSS11A, CFI, C2, HGFAC
GOTERM_BP_5	GO:0050864~regulation of B cell activation	9	6.79E-04	CD38, CDKN1A, FCGR2B, BCL6, TLR4, IL2RG, IGH-6, FAS, CD28
GOTERM_BP_5	GO:0048514~blood vessel morphogenesis	18	7.18E-04	MEF2C, CAV1, PGF, MMP19, ENPEP, TNNI3, MMP14, WT1, EDNRA, MEOX2, DLL4, CASP8, TGM2, SEMA3C, SOX18, ANGPTL3, FIGF, TNFAIP2
GOTERM_BP_5	GO:0019221~cytokine-mediated signaling pathway	9	7.68E-04	CEBPA, IL1R1, CCL2, IL1RAP, CX3CR1, LIFR, IIGP1, CSF2RB, STAT3
GOTERM_MF_5	GO:0004896~cytokine receptor activity	9	8.19E-04	IL1R1, OSMR, IL1RAP, CX3CR1, IL11RA2, LIFR, CSF2RB, IL2RG, IL13RA1
GOTERM_BP_5	GO:0030195~negative regulation	4	8.28E-04	KLKB1, TMPRSS6, CPB2, PLG

	of blood coagulation			
GOTERM_CC_5	GO:0030018~Z disc	8	8.58E-04	KRT19, PGM5, PDLIM5, ITGB1BP2, LDB3, MYOZ2, TTN, CSRP3
GOTERM_BP_5	GO:0002026~regulation of the force of heart contraction	5	8.78E-04	PRKCA, ATP2A2, PLN, MYH6, CSRP3
**GOTERM_CC_5**	**GO:0016529~sarcoplasmic reticulum**	**7**	**9.01E-04**	**TRDN, TMEM109, SLN, ATP2A2, PLN, MRVI1, CASQ1**
**GOTERM_BP_5**	**GO:0007507~heart development**	**19**	**0.001040263**	**MEF2C, MYBPC3, SMYD1, MYH6, TNNI3, TTN, CXADR, CSRP3, VCAM1, EDNRA, HHEX, OSR1, KCNJ8, PLN, CASP8, PKD2, SEMA3C, HTR2B, MB**
GOTERM_BP_5	GO:0030003~cellular cation homeostasis	15	0.00105516	PRKCA, CAV1, PTGER3, CCL2, PRND, TNNI3, 1300017J02RIK, CSRP3, TRF, ATP2A2, PLN, PKD2, TGM2, RGN, TRFR2
GOTERM_BP_5	GO:0015918~sterol transport	6	0.001106602	APOA1, CD36, MSR1, CFTR, APOM, GPIHBP1
GOTERM_BP_5	GO:0050766~positive regulation of phagocytosis	6	0.001106602	FCGR2B, C3, FCER1G, CLEC7A, FCGR1, AHSG
GOTERM_BP_5	GO:0030301~cholesterol transport	6	0.001106602	APOA1, CD36, MSR1, CFTR, APOM, GPIHBP1
GOTERM_BP_5	GO:0045807~positive regulation of endocytosis	7	0.00113473	FCGR2B, C3, FCER1G, IGH-6, CLEC7A, FCGR1, AHSG
GOTERM_BP_5	GO:0001952~regulation of cell-matrix adhesion	5	0.001194541	ONECUT1, ONECUT2, BCL6, MMP14, PIK3R1
GOTERM_CC_5	GO:0016528~sarcoplasm	7	0.001231652	TRDN, TMEM109, SLN, ATP2A2, PLN, MRVI1, CASQ1
GOTERM_BP_5	GO:0002009~morphogenesis of an epithelium	16	0.001314192	EGFR, KRT6A, PGF, HOXD13, NR3C1, JAG1, GREM1, FZD6, CCL11, ALDH1A1, HHEX, TCF21, WNT4, CASP8, TRP63, SEMA3C
KEGG_PATHWAY	mmu04510:Focal adhesion	21	0.001340283	EGFR, PRKCA, CAV3, CAV2, MYL7, CAV1, PGF, MYLK3, MET, ITGA1, VTN, VCL, LAMA2, ITGB6, COL6A2, PDGFC, COL1A1, FIGF, THBS2, MYLK, PIK3R1
GOTERM_BP_5	GO:0010812~negative regulation of cell-substrate adhesion	4	0.001410263	BCL6, COL1A1, MMP14, PIK3R1
GOTERM_BP_5	GO:0032760~positive regulation of tumor necrosis factor production	5	0.001583467	FCER1G, TLR4, CLEC7A, LBP, CD14
GOTERM_BP_5	GO:0007162~negative regulation of cell adhesion	6	0.001647772	B4GALNT2, MYO1F, BCL6, COL1A1, MMP14, PIK3R1
GOTERM_BP_5	GO:0050764~regulation of phagocytosis	6	0.001647772	FCGR2B, C3, FCER1G, CLEC7A, FCGR1, AHSG
**GOTERM_CC_5**	**GO:0015629~actin cytoskeleton**	**17**	**0.001759674**	**MYL7, AIF1, ACTA2, MYBPC3, MYO1D, MYO1F, MYOZ2, MYH6, TTN, PALLD, VCL, ARPC1B, SPNB2, SVIL, GYS2, MYOM1, SYNPO**
GOTERM_BP_5	GO:0050871~positive regulation of B cell activation	7	0.001792603	CD38, CDKN1A, BCL6, TLR4, IL2RG, IGH-6, CD28
GOTERM_BP_5	GO:0048732~gland development	17	0.001824055	EGFR, CAV1, CEBPB, ELF5, MET, TGFB3, HOXD13, NR3C1, CCL11, HHEX, WNT4, APOA1, IRF6, XBP1, TRP63, TGM2, SEMA3C
GOTERM_MF_5	GO:0008236~serine-type	18	0.00203693	F11, F12, F10, MASP1, PAMR1,

	peptidase activity		4	MST1, C1S, CELA1, TMPRSS6, PLG, PGM5, KLKB1, PROZ, F2, TMPRSS11A, CFI, C2, HGFAC
GOTERM_BP_5	GO:0030168~platelet activation	5	0.00205245 1	FGG, PLEK, FGA, FGB, F2
GOTERM_BP_5	GO:0032680~regulation of tumor necrosis factor production	6	0.00236144 3	TLR1, FCER1G, TLR4, CLEC7A, LBP, CD14
GOTERM_CC_5	GO:0045121~membrane raft	10	0.00236529 1	CAV3, CAV2, CAV1, CD36, PTRF, SDPR, PRND, FCER1G, ITGB2, CD14
GOTERM_BP_5	GO:0012502~induction of programmed cell death	15	0.00257658 5	PRKCA, CEBPB, C9, TGFB3, CIDEB, MAL, FCGR1, PLG, CASP12, TRP63, TGM2, FAS, PERP, CASP1, CD28
GOTERM_BP_5	GO:0030593~neutrophil chemotaxis	5	0.00260926 9	PRKCA, CXCL15, ITGA1, FCER1G, ITGB2
GOTERM_BP_5	GO:0051272~positive regulation of cell motion	7	0.00307585 2	ONECUT1, ONECUT2, MYO1F, BCL6, ANGPTL3, PIK3R1, CXCL10
GOTERM_BP_5	GO:0019627~urea metabolic process	4	0.00320881 8	CEBPA, ARG1, OTC, CPS1
GOTERM_BP_5	GO:0002861~regulation of inflammatory response to antigenic stimulus	5	0.00326151 5	FCGR2B, C3, FCER1G, FCGR1, CD28
GOTERM_BP_5	GO:0050873~brown fat cell differentiation	6	0.00327663 3	CEBPA, RARRES2, CEBPB, FABP4, SELENBP1, MB
GOTERM_BP_5	GO:0043068~positive regulation of programmed cell death	19	0.00363320 4	PRKCA, CEBPB, C9, CIDEB, TGFB3, MAL, NR3C1, FCGR1, PLG, ALDH1A1, CDKN1A, CASP12, TRP63, TGM2, FAS, PERP, CASP1, IGFBP3, CD28
GOTERM_BP_5	GO:0010942~positive regulation of cell death	19	0.00398113	PRKCA, CEBPB, C9, CIDEB, TGFB3, MAL, NR3C1, FCGR1, PLG, ALDH1A1, CDKN1A, CASP12, TRP63, TGM2, FAS, PERP, CASP1, IGFBP3, CD28
GOTERM_BP_5	GO:0002697~regulation of immune effector process	10	0.00402143 4	FCGR2B, C3, HPX, CFH, FCER1G, BCL6, SERPING1, LBP, FCGR1, CD28
GOTERM_BP_5	GO:0050801~ion homeostasis	21	0.00412960 5	PRKCA, CAV3, SLC9A9, CAV1, CCL2, PTGER3, PRND, HFE, TNNI3, CSRP3, 1300017J02RIK, TRF, DMPK, ATP2A2, PLN, NAB2, PKD2, TGM2, RGN, SLC4A4, TRFR2
GOTERM_BP_5	GO:0043604~amide biosynthetic process	4	0.00446370 9	CEBPA, ARG1, OTC, CPS1
GOTERM_BP_5	GO:0042116~macrophage activation	4	0.00446370 9	CX3CR1, TLR1, TLR4, LBP
**KEGG_PATHWAY**	**mmu03320:PPAR signaling pathway**	**11**	**0.00450511**	**LPL, APOA2, APOA1, CD36, HMGCS2, OLR1, EHHADH, FABP4, FABP1, FABP2, ACSL5**
**KEGG_PATHWAY**	**mmu05414:Dilated cardiomyopathy**	**12**	**0.00459945 4**	**LAMA2, ATP2A2, ADCY7, PLN, ITGB6, MYBPC3, TGFB3, ITGA1, LMNA, MYH6, TNNI3, TTN**
GOTERM_CC_5	GO:0042383~sarcolemma	7	0.00495685 3	CAV3, LAMA2, KRT19, KCNJ8, COL6A2, TGFB3, SSPN
GOTERM_BP_5	GO:0060191~regulation of lipase activity	6	0.00509125 9	APOA2, PTGER3, TGM2, NPR3, HTR2B, ANGPTL3
GOTERM_BP_5	GO:0001892~embryonic placenta development	8	0.00574538 9	CEBPA, EGFR, VCAM1, KRT19, CEBPB, PKD2, PCDH12, PLG
GOTERM_BP_5	GO:0002675~positive regulation of acute inflammatory response	4	0.00597680 5	C3, FCER1G, LBP, FCGR1

KEGG_PATHWAY	mmu05020:Prion diseases	7	0.006062319	C1QA, C8A, C1QB, C9, HC, CASP12, C1QC
**GOTERM_BP_5**	**GO:0030324~lung development**	**11**	**0.006181926**	**CEBPA, MAN2A1, TCF21, HHEX, CRISPLD2, TGFB3, MGP, CFTR, LOX, MMP14, ADAMTS2**
GOTERM_BP_5	GO:0050880~regulation of blood vessel size	7	0.006813616	KNG1, EDNRA, CAV1, KCNJ8, ACTA2, GUCY1A3, CFTR
GOTERM_BP_5	GO:0010959~regulation of metal ion transport	7	0.006813616	CAV3, CAV1, SLN, PLN, PKD2, NEDD4L, DMPK
GOTERM_CC_5	GO:0031672~A band	4	0.006853692	SPNB2, MYBPC3, MYOM1, TTN
KEGG_PATHWAY	mmu00500:Starch and sucrose metabolism	7	0.006986143	ENPP1, UGT2B34, HK3, MGAM, GYS2, UGT1A1, UGP2
GOTERM_BP_5	GO:0030323~respiratory tube development	11	0.006995937	CEBPA, MAN2A1, TCF21, HHEX, CRISPLD2, TGFB3, MGP, CFTR, LOX, MMP14, ADAMTS2
GOTERM_BP_5	GO:0032490~detection of molecule of bacterial origin	3	0.00723735	LY96, TLR1, TLR4
KEGG_PATHWAY	mmu04514:Cell adhesion molecules (CAMs)	16	0.007328262	CLDN8, H2-Q10, OCLN, ICAM2, CLDN5, CD99, ITGB2, CLDN23, CDH5, ALCAM, VCAM1, CD86, CD34, CLDN1, ESAM, CD28
GOTERM_BP_5	GO:0055082~cellular chemical homeostasis	19	0.007464642	PRKCA, CAV3, CAV1, PTGER3, CCL2, FOXA3, PRND, TNNI3, CSRP3, 1300017J02RIK, TRF, DMPK, ATP2A2, PLN, NAB2, PKD2, TGM2, RGN, TRFR2
GOTERM_BP_5	GO:0043065~positive regulation of apoptosis	18	0.007565382	PRKCA, CEBPB, C9, CIDEB, TGFB3, MAL, NR3C1, FCGR1, PLG, ALDH1A1, CASP12, TRP63, TGM2, FAS, PERP, CASP1, IGFBP3, CD28
GOTERM_BP_5	GO:0007398~ectoderm development	12	0.007897887	EGFR, NTF5, KRT6A, SPRR1A, IRF6, PPL, ELF5, PTGS1, TRP63, COL1A1, ADAMTS2, SCEL
GOTERM_CC_5	GO:0031226~intrinsic to plasma membrane	31	0.007934438	CAV2, CAV1, C9, SGMS2, OSMR, ITGB2, AQP1, LAPTM5, ITGB6, SCN9A, FCER1G, SCN7A, SLC4A4, PPAP2A, GPIHBP1, TMEM45A, KCNE4, CLCA1, HC, ICAM2, SLC6A13, ITGA1, PCDH12, UGT1A1, C8A, FCGR2B, KCNJ8, DLL4, TMPRSS11A, CP, PERP
GOTERM_BP_5	GO:0009968~negative regulation of signal transduction	14	0.008156779	PRKCA, CAV3, CAV1, NEPN, ONECUT1, ONECUT2, TGFB3, GREM1, AHSG, HHEX, WNT4, HNF4A, BCL6, LECT2
GOTERM_BP_5	GO:0031960~response to corticosteroid stimulus	5	0.008201729	ALDOB, FABP4, COL1A1, FAS, UGT1A1
GOTERM_BP_5	GO:0008016~regulation of heart contraction	7	0.008314216	PRKCA, ATP2A2, PLN, MYBPC3, MYH6, CSRP3, DMPK
**GOTERM_BP_5**	**GO:0009913~epidermal cell differentiation**	**7**	**0.009147318**	**KRT6A, SPRR1A, IRF6, PPL, PTGS1, TRP63, SCEL**
GOTERM_BP_5	GO:0060537~muscle tissue development	12	0.009275366	MEF2C, CAV2, CAV1, MEOX2, PLN, MET, TRP63, MYH6, MBNL1, TTN, CXADR, CSRP3
GOTERM_BP_5	GO:0030335~positive regulation	6	0.00953206	ONECUT1, ONECUT2, MYO1F,

	of cell migration			ANGPTL3, PIK3R1, CXCL10
GOTERM_BP_5	GO:0002863~positive regulation of inflammatory response to antigenic stimulus	4	0.009825557	C3, FCER1G, FCGR1, CD28
GOTERM_BP_5	GO:0002864~regulation of acute inflammatory response to antigenic stimulus	4	0.009825557	FCGR2B, C3, FCER1G, FCGR1
GOTERM_BP_5	GO:0022612~gland morphogenesis	9	0.009949627	CCL11, EGFR, WNT4, CAV1, TRP63, TGM2, SEMA3C, HOXD13, NR3C1
GOTERM_BP_5	GO:0030100~regulation of endocytosis	7	0.010038399	FCGR2B, C3, FCER1G, IGH-6, CLEC7A, FCGR1, AHSG
GOTERM_CC_5	GO:0030054~cell junction	27	0.010384123	CLDN8, OCLN, CLDN5, CXADR, GJA4, CDH5, GJA3, VCL, PVRL4, MARVELD2, PPL, ESAM, AHNAK, SYNPO, TMEM204, CLCA5, PCDH12, SSPN, CLDN23, GJB1, GJB2, PGM5, TNS1, CLDN1, DSC2, PERP, PDZD2
GOTERM_CC_5	GO:0005614~interstitial matrix	4	0.010780733	SMOC2, NEPN, CCDC80, ABI3BP
GOTERM_CC_5	GO:0005579~membrane attack complex	3	0.010798161	C8A, C9, HC
GOTERM_BP_5	GO:0002064~epithelial cell development	5	0.011079701	WNT4, ONECUT1, XBP1, ONECUT2, TRP63
GOTERM_CC_5	GO:0031225~anchored to membrane	15	0.011351616	LPL, PRND, MMP19, CD109, CD52, LYPD6B, VCAM1, ART3, PPP1R16B, CP, RAB27B, NT5E, GPIHBP1, DPEP1, CD14
GOTERM_BP_5	GO:0030879~mammary gland development	9	0.011392161	CCL11, WNT4, CAV1, CEBPB, IRF6, ELF5, MET, TGFB3, NR3C1
GOTERM_BP_5	GO:0070391~response to lipoteichoic acid	3	0.011778855	TLR4, LBP, CD14
GOTERM_BP_5	GO:0050665~hydrogen peroxide biosynthetic process	3	0.011778855	CYBB, CYP1A1, NCF1
GOTERM_BP_5	GO:0015711~organic anion transport	6	0.011882983	PTGER3, SLC16A7, SLC26A1, SLCO2B1, SLC4A4, ARL6IP5
GOTERM_CC_5	GO:0005604~basement membrane	8	0.011928913	LAMA2, SMOC2, ALB, CCDC80, TIMP3, COL8A2, TRF, MMRN2
GOTERM_BP_5	GO:0016477~cell migration	17	0.01203222	EGFR, PRKCA, CCKAR, CCR1, MET, ITGA1, ITGB2, ENPEP, MMP14, APOA1, TNS1, CD34, CXCL15, CX3CR1, FCER1G, SEMA3C, LBP
GOTERM_BP_5	GO:0001890~placenta development	9	0.012168671	CEBPA, EGFR, VCAM1, KRT19, CEBPB, MET, PKD2, PCDH12, PLG
GOTERM_BP_5	GO:0008544~epidermis development	11	0.013751433	EGFR, NTF5, KRT6A, SPRR1A, IRF6, PPL, PTGS1, TRP63, COL1A1, ADAMTS2, SCEL
GOTERM_BP_5	GO:0042981~regulation of apoptosis	31	0.014153998	IFIH1, C9, PRND, CLU, TGFB3, NR3C1, ALDH1A1, ALB, CASP8, TRP63, TGM2, DAD1, FCER1G, BCL6, FAS, CASP1, PIK3R1, CD28, PRKCA, CEBPB, CIDEB, MAL, FCGR1, PLG, ATF5, CDKN1A, BCL2A1A, CASP12, CX3CR1, PERP, IGFBP3
GOTERM_CC_5	GO:0005901~caveola	5	0.014198835	CAV3, CAV2, CAV1, PTRF, SDPR
GOTERM_BP_5	GO:0002822~regulation of	7	0.01422281	FCGR2B, C3, HPX, FCER1G, BCL6,

	adaptive immune response based on somatic recombination of immune receptors built from immunoglobulin superfamily domains		7	FCGR1, CD28
GOTERM_BP_5	GO:0002819~regulation of adaptive immune response	7	0.014222817	FCGR2B, C3, HPX, FCER1G, BCL6, FCGR1, CD28
GOTERM_CC_5	GO:0005887~integral to plasma membrane	29	0.014571165	CAV2, CAV1, CLCA1, C9, SGMS2, OSMR, HC, ICAM2, SLC6A13, ITGA1, PCDH12, ITGB2, AQP1, UGT1A1, C8A, LAPTM5, FCGR2B, KCNJ8, DLL4, ITGB6, SCN9A, FCER1G, TMPRSS11A, SCN7A, PPAP2A, SLC4A4, PERP, KCNE4, TMEM45A
GOTERM_BP_5	GO:0045576~mast cell activation	4	0.014828472	FCGR2B, FCER1G, TLR4, LCP2
GOTERM_BP_5	GO:0006911~phagocytosis, engulfment	4	0.014828472	FCGR2B, FCER1G, CLEC7A, FCGR1
GOTERM_BP_5	GO:0042742~defense response to bacterium	10	0.014829352	PLD1, CCR5, WFDC15B, NCF1, MYO1F, FCER1G, TLR4, LBP, FCGR1, LEAP2
GOTERM_BP_5	GO:0014706~striated muscle tissue development	11	0.015236953	MEF2C, CAV2, CAV1, MEOX2, PLN, MET, MYH6, MBNL1, TTN, CXADR, CSRP3
GOTERM_BP_5	GO:0010648~negative regulation of cell communication	14	0.015723015	PRKCA, CAV3, CAV1, NEPN, ONECUT1, ONECUT2, TGFB3, GREM1, AHSG, HHEX, WNT4, HNF4A, BCL6, LECT2
GOTERM_BP_5	GO:0043067~regulation of programmed cell death	31	0.016558701	IFIH1, C9, PRND, CLU, TGFB3, NR3C1, ALDH1A1, ALB, CASP8, TRP63, TGM2, DAD1, FCER1G, BCL6, FAS, CASP1, PIK3R1, CD28, PRKCA, CEBPB, CIDEB, MAL, FCGR1, PLG, ATF5, CDKN1A, BCL2A1A, CASP12, CX3CR1, PERP, IGFBP3
GOTERM_BP_5	GO:0030334~regulation of cell migration	9	0.016644851	LAMA2, ONECUT1, DLL4, ONECUT2, MYO1F, ANGPTL3, PIK3R1, VCL, CXCL10
**GOTERM_CC_5**	**GO:0016459~myosin complex**	**7**	**0.017238632**	**MYL7, MYO1D, MYBPC3, MYO1F, MYH6, MYOM1, TTN**
GOTERM_BP_5	GO:0031100~organ regeneration	3	0.017254428	APOH, GAS6, AHSG
GOTERM_BP_5	GO:0001953~negative regulation of cell-matrix adhesion	3	0.017254428	BCL6, MMP14, PIK3R1
GOTERM_BP_5	GO:0060644~mammary gland epithelial cell differentiation	3	0.017254428	CEBPB, IRF6, ELF5
GOTERM_BP_5	GO:0006820~anion transport	11	0.017684987	P2RY6, CLCA1, PTGER3, SLC16A7, CLCA3, CLCA5, CFTR, SLC26A1, SLCO2B1, SLC4A4, ARL6IP5
GOTERM_BP_5	GO:0001889~liver development	6	0.017703312	CEBPA, MAN2A1, HHEX, ONECUT1, ONECUT2, MET
GOTERM_BP_5	GO:0060571~morphogenesis of an epithelial fold	4	0.017776785	EGFR, HHEX, TRP63, HOXD13
GOTERM_BP_5	GO:0030512~negative regulation of transforming growth factor beta receptor signaling pathway	4	0.017776785	NEPN, ONECUT1, ONECUT2, TGFB3

GOTERM_BP_5	GO:0042594~response to starvation	5	0.018610549	ALB, FOXA3, ALDOB, HFE, UGT1A1
GOTERM_BP_5	GO:0051050~positive regulation of transport	11	0.019470801	CAV1, FCGR2B, C3, TGFB3, FCER1G, SYTL2, IGH-6, CLEC7A, CASP1, FCGR1, AHSG
KEGG_PATHWAY	mmu05410:Hypertrophic cardiomyopathy (HCM)	10	0.019645895	LAMA2, ATP2A2, ITGB6, MYBPC3, TGFB3, ITGA1, LMNA, MYH6, TNNI3, TTN
GOTERM_BP_5	GO:0001525~angiogenesis	11	0.020411688	EDNRA, MEOX2, PGF, DLL4, MMP19, CASP8, SOX18, ENPEP, MMP14, FIGF, TNFAIP2
GOTERM_CC_5	GO:0031430~M band	3	0.021672719	SPNB2, MYOM1, TTN
GOTERM_CC_5	GO:0042598~vesicular fraction	13	0.021934836	AADAC, ATP2A2, PTRF, CYP1A1, KCNJ8, FMO1, PTGS1, CYP2J6, B4GALNT2, UGT1A1, DNAJC1, PLG, ACSL5
GOTERM_BP_5	GO:0043269~regulation of ion transport	7	0.022565839	CAV3, CAV1, SLN, PLN, PKD2, NEDD4L, DMPK
GOTERM_BP_5	GO:0045088~regulation of innate immune response	6	0.0231287	HPX, MYO1F, TLR4, SERPING1, CLEC7A, LBP
GOTERM_BP_5	GO:0048729~tissue morphogenesis	16	0.023283933	EGFR, KRT6A, PGF, HOXD13, NR3C1, JAG1, GREM1, FZD6, CCL11, ALDH1A1, HHEX, TCF21, WNT4, CASP8, TRP63, SEMA3C
GOTERM_BP_5	GO:0042953~lipoprotein transport	3	0.023592095	CD36, MSR1, APOBEC1
GOTERM_CC_5	GO:0070161~anchoring junction	10	0.024586239	PVRL4, TMEM204, TNS1, PGM5, PPL, DSC2, ESAM, CXADR, PERP, VCL
GOTERM_CC_5	GO:0005922~connexon complex	4	0.025312676	GJA4, GJB1, GJA3, GJB2
GOTERM_BP_5	GO:0050867~positive regulation of cell activation	9	0.027503003	CD38, CDKN1A, PLEK, FCER1G, BCL6, TLR4, IL2RG, IGH-6, CD28
GOTERM_BP_5	GO:0008217~regulation of blood pressure	7	0.027764889	EDNRA, ACTA2, PTGS1, GUCY1A3, MYH6, NPR3, TNNI3
GOTERM_BP_5	GO:0002706~regulation of lymphocyte mediated immunity	7	0.027764889	FCGR2B, C3, HPX, FCER1G, BCL6, FCGR1, CD28
GOTERM_BP_5	GO:0060627~regulation of vesicle-mediated transport	8	0.028220311	RAB3D, FCGR2B, C3, FCER1G, IGH-6, CLEC7A, FCGR1, AHSG
GOTERM_BP_5	GO:0015698~inorganic anion transport	8	0.028220311	P2RY6, CLCA1, PTGER3, CLCA3, CLCA5, CFTR, SLC26A1, SLC4A4
GOTERM_BP_5	GO:0043627~response to estrogen stimulus	5	0.028702717	CAV1, TGFB3, CFTR, MMP14, TIMP3
GOTERM_BP_5	GO:0006909~phagocytosis	6	0.029527071	CD36, FCGR2B, FCER1G, CLEC7A, LBP, FCGR1
GOTERM_BP_5	GO:0002237~response to molecule of bacterial origin	6	0.029527071	KCNJ8, LY96, TLR1, TLR4, LBP, CD14
GOTERM_BP_5	GO:0001701~in utero embryonic development	17	0.029634844	CEBPA, EGFR, CEBPB, HC, TGFB3, PCDH12, MBNL1, MYH6, TTN, PLG, VCAM1, EDNRA, MAN2A1, KRT19, SERPINA1B, PKD2, DAD1
KEGG_PATHWAY	mmu04062:Chemokine signaling pathway	16	0.029920209	CCL2, ADCY7, NCF1, CCR1, CCL9, CCL7, STAT3, CCL6, CXCL10, CCL11, CCR5, PPBP, CXCL15, CX3CR1, GRK5, PIK3R1
GOTERM_BP_5	GO:0002888~positive regulation	3	0.03072379	C3, FCER1G, FCGR1

	of myeloid leukocyte mediated immunity		9	
GOTERM_BP_5	GO:0055088~lipid homeostasis	5	0.031644717	CAV3, APOA2, CAV1, FABP4, ANGPTL3
GOTERM_BP_5	GO:0006631~fatty acid metabolic process	13	0.031822296	ECH1, NCF1, EHHADH, PTGS1, FADS3, ACADVL, APOA2, FABP4, HPGD, HPGDS, TMEM195, MGST2, ACSL5
GOTERM_BP_5	GO:0050671~positive regulation of lymphocyte proliferation	6	0.031884054	CD38, CDKN1A, BCL6, TLR4, IGH-6, CD28
GOTERM_BP_5	GO:0032946~positive regulation of mononuclear cell proliferation	6	0.031884054	CD38, CDKN1A, BCL6, TLR4, IGH-6, CD28
GOTERM_BP_5	GO:0009887~organ morphogenesis	31	0.0338176	MEF2C, KRT6A, PGF, MYBPC3, TGFB3, HOXD13, NR3C1, JAG1, GREM1, TTN, AHSG, ALDH1A1, TCF21, WNT4, CASP8, TRP63, APOH, SEMA3C, PDGFC, COL8A2, EGFR, NTF5, GAS6, STAT3, FZD6, CCL11, HHEX, HOXD8, NAB2, GAMT, COL1A1
GOTERM_BP_5	GO:0031399~regulation of protein modification process	12	0.033881224	EGFR, PRKCA, CAV1, APOA1, HNF4A, HPX, MDFIC, TGFB3, PDGFC, FBXO4, IGH-6, IGFBP3
KEGG_PATHWAY	mmu04950:Maturity onset diabetes of the young	5	0.034741141	HHEX, HNF4A, ONECUT1, FOXA3, SLC2A2
GOTERM_BP_5	GO:0070665~positive regulation of leukocyte proliferation	6	0.036942358	CD38, CDKN1A, BCL6, TLR4, IGH-6, CD28
GOTERM_BP_5	GO:0001822~kidney development	9	0.037006515	TCF21, WNT4, OSR1, PGF, KCNJ8, PKD2, GREM1, ANXA4, WT1
GOTERM_BP_5	GO:0051781~positive regulation of cell division	5	0.038041146	S100A6, PGF, TGFB3, PDGFC, FIGF
GOTERM_BP_5	GO:0033559~unsaturated fatty acid metabolic process	5	0.038041146	NCF1, PTGS1, HPGD, HPGDS, MGST2
GOTERM_BP_5	GO:0002703~regulation of leukocyte mediated immunity	7	0.038078601	FCGR2B, C3, HPX, FCER1G, BCL6, FCGR1, CD28
GOTERM_BP_5	GO:0031016~pancreas development	5	0.041496792	HHEX, ONECUT1, XBP1, ONECUT2, CLU
GOTERM_BP_5	GO:0009306~protein secretion	4	0.041796606	TRAF3IP2, PLEK, LCP2, CXCL10
GOTERM_BP_5	GO:0051259~protein oligomerization	7	0.042805282	C1QTNF7, CAV2, CAV1, OTC, PRND, TGM2, FAS
GOTERM_CC_5	GO:0005624~membrane fraction	26	0.04313121	CAV3, CAV1, CYP2J6, PTGS1, ITGB2, ASAH2, FMO1, B4GALNT2, PPAP2A, EHD2, NT5E, DNAJC1, ACSL5, CYP1A1, NCF1, MET, NPR3, UGT1A1, PLG, AADAC, CD38, SLC16A7, ATP2A2, PTRF, KCNJ8, SYTL2
GOTERM_BP_5	GO:0060562~epithelial tube morphogenesis	9	0.044441762	CCL11, TCF21, HHEX, WNT4, PGF, CASP8, NR3C1, GREM1, FZD6
GOTERM_BP_5	GO:0032496~response to lipopolysaccharide	5	0.045124262	KCNJ8, LY96, TLR4, LBP, CD14
KEGG_PATHWAY	mmu00830:Retinol metabolism	8	0.045188259	ALDH1A1, DHRS3, CYP1A1, UGT2B34, CYP2C68, ALDH1A7, UGT1A1, RDH5
KEGG_PATHWAY	mmu00040:Pentose and glucuronate interconversions	4	0.046215616	AKR1B7, UGT2B34, UGT1A1, UGP2

GOTERM_BP_5	GO:0050830~defense response to Gram-positive bacterium	4	0.046835838	PLD1, NCF1, MYO1F, LBP
GOTERM_BP_5	GO:0043066~negative regulation of apoptosis	15	0.046842895	CEBPB, PRND, CLU, TGFB3, ATF5, CDKN1A, BCL2A1A, ALB, CX3CR1, TRP63, DAD1, FCER1G, BCL6, FAS, PIK3R1
GOTERM_BP_5	GO:0008211~glucocorticoid metabolic process	3	0.047115601	APOA1, SERPINA6, NR3C1
GOTERM_BP_5	GO:0002866~positive regulation of acute inflammatory response to antigenic stimulus	3	0.047115601	C3, FCER1G, FCGR1
GOTERM_BP_5	GO:0048754~branching morphogenesis of a tube	8	0.04783648	CCL11, EDNRA, TCF21, HHEX, WNT4, PGF, MMP14, GREM1
KEGG_PATHWAY	mmu04620:Toll-like receptor signaling pathway	10	0.049540403	CD86, LY96, RIPK1, CASP8, TLR1, TLR4, LBP, PIK3R1, CD14, CXCL10
KEGG_PATHWAY	mmu04640:Hematopoietic cell lineage	9	0.049554924	CD38, IL1R1, CD36, CD34, IL11RA2, ITGA1, FCGR1, CD14, CSF1R
**Cluster 7**				
**Category**	**Term**	**Count**	**PValue**	**Genes**
**GOTERM_BP_5**	**GO:0019827~stem cell maintenance**	**9**	**2.69E-09**	**NANOG, RIF1, ESRRB, POU5F1, NODAL, SOX2, PIWIL2, FGF4, TCL1**
**GOTERM_BP_5**	**GO:0048864~stem cell development**	**9**	**4.07E-09**	**NANOG, RIF1, ESRRB, POU5F1, NODAL, SOX2, PIWIL2, FGF4, TCL1**
**GOTERM_CC_5**	**GO:0005634~nucleus**	**115**	**1.94E-07**	**TEX19.1, XRCC5, APOBEC3, INO80, GM13138, GM13139, REST, CBX7, SOHLH2, ZIC3, SETX, NAA11, EED, STAG3, PITPNC1, CRY1, DHX32, MYST4, RPP25, TCFAP2C, I1C0022H11RIK, PIM1, NR0B1, CPHX, SUZ12, 2210409E12RIK, MTF2, HSPB1, TESK2, PIAS2, PRDM1, RNF17, SOX2, MYBL2, PSMA8, COIL, IRAK3, ZFP819, SPNB4, DNMT3L, RANBP17, MAFF, UTF1, CCPG1, KLF9, LGALS3, ESRRB, SMYD3, SYCE1, TRIM63, DPPA4, TET1, ZBTB44, SFMBT2, DPPA2, TCL1, PHF3, AIRE, RFX2, RBPJ, KLF2, TCF15, CDC14A, ZMAT4, ZFP42, MLH3, CDT1, NFATC2IP, ANKRD17, SAP30, ASH2L, POU5F1, GPX4, SOX15, IPMK, NANOG, TRAP1A, ARID5B, DMRT1, TLE4, MORC1, GM13152, ZSWIM1, ZFP655, RIF1, DMC1, GADD45A, GRASP, CAMK1D, ZFP710, CALCOCO2, TRIB3, GM13212, TCFCP2L1, STAT4, TCEA3, ANKRD37, GBX2, KDM3B, ETV5, D14ERTD668E, MSH6, KAT2B, REX2, TBX3, NF2, JARID2, ZFP59, SIRT1, RPS6KA5, 1700029I01RIK, PHF17, CENPV, RHOX13, 2610305D13RIK, USP48, ZFP534, NR5A2**
GOTERM_BP_5	GO:0010556~regulation of	73	2.82E-06	ZFP42, GM13138, GM13139, REST,

	macromolecule biosynthetic process			CBX7, ZIC3, SOHLH2, CDT1, NFATC2IP, SAP30, EIF4EBP2, STRA8, ASH2L, POU5F1, EED, PIWIL2, SOX15, CRY1, MYST4, NANOG, TCFAP2C, ARID5B, NODAL, I1C0022H11RIK, DMRT1, TLE4, NR0B1, CPHX, SUZ12, GM13152, ZFP655, PIAS2, PRDM1, ZFP710, SOX2, TRIB3, MYBL2, GM13212, TCFCP2L1, IRAK3, STAT4, ZFP819, TCEA3, GBX2, DAZL, INPP5D, KDM3B, ETV5, D14ERTD668E, MAFF, UTF1, KAT2B, KLF9, TBX3, REX2, JARID2, ESRRB, SYKB, ZFP59, ZBTB44, SIRT1, SFMBT2, FXR1, RPS6KA5, 1700029I01RIK, PHF17, AIRE, RHOX13, ZFP534, 2610305D13RIK, RFX2, NR5A2, KLF2, RBPJ, TCF15
GOTERM_BP_5	GO:0045596~negative regulation of cell differentiation	15	5.80E-06	NANOG, TBX3, ESRRB, NODAL, SOX2, REST, NR0B1, SIRT1, TCL1, RIF1, POU5F1, PIWIL2, INPP5D, RBPJ, FGF4
GOTERM_BP_5	GO:0010605~negative regulation of macromolecule metabolic process	25	1.66E-05	MSH6, NANOG, TBX3, JARID2, NODAL, SOX2, TLE4, REST, NR0B1, SIRT1, FXR1, TCFCP2L1, CDT1, SUZ12, SAP30, IRAK3, MOV10, EIF4EBP2, STRA8, POU5F1, PIWIL2, SOX15, INPP5D, RBPJ, MYST4
**GOTERM_BP_5**	**GO:0007283~spermatogenesis**	**16**	**6.50E-05**	**RNF17, SLCO4C1, NR0B1, MORC1, SIRT1, SOHLH2, BBS2, CLGN, STRA8, GPX4, D1PAS1, ZFP296, TESK2, PIWIL2, DAZL, DMC1**
GOTERM_BP_5	GO:0045449~regulation of transcription	65	6.62E-05	ZFP42, GM13138, GM13139, REST, CBX7, ZIC3, SOHLH2, NFATC2IP, SAP30, ASH2L, POU5F1, EED, SOX15, CRY1, MYST4, NANOG, TCFAP2C, ARID5B, NODAL, I1C0022H11RIK, DMRT1, TLE4, NR0B1, CPHX, SUZ12, GM13152, ZFP655, PIAS2, PRDM1, ZFP710, SOX2, TRIB3, MYBL2, GM13212, TCFCP2L1, IRAK3, STAT4, ZFP819, TCEA3, GBX2, KDM3B, ETV5, D14ERTD668E, MAFF, UTF1, KAT2B, KLF9, TBX3, REX2, JARID2, ESRRB, ZFP59, ZBTB44, SIRT1, SFMBT2, RPS6KA5, 1700029I01RIK, PHF17, AIRE, RHOX13, ZFP534, 2610305D13RIK, RFX2, NR5A2, KLF2, RBPJ, TCF15
GOTERM_BP_5	GO:0010558~negative regulation of macromolecule biosynthetic process	21	7.64E-05	NANOG, TBX3, JARID2, NODAL, SOX2, TLE4, REST, NR0B1, SIRT1, FXR1, TCFCP2L1, CDT1, SUZ12, SAP30, EIF4EBP2, STRA8, POU5F1, SOX15, INPP5D, RBPJ, MYST4
GOTERM_BP_5	GO:0031327~negative regulation of cellular biosynthetic process	21	1.13E-04	NANOG, TBX3, JARID2, NODAL, SOX2, TLE4, REST, NR0B1, SIRT1, FXR1, TCFCP2L1, CDT1, SUZ12, SAP30, EIF4EBP2, STRA8, POU5F1,

				SOX15, INPP5D, RBPJ, MYST4
GOTERM_BP_5	GO:0009890~negative regulation of biosynthetic process	21	1.29E-04	NANOG, TBX3, JARID2, NODAL, SOX2, TLE4, REST, NR0B1, SIRT1, FXR1, TCFCP2L1, CDT1, SUZ12, SAP30, EIF4EBP2, STRA8, POU5F1, SOX15, INPP5D, RBPJ, MYST4
GOTERM_BP_5	GO:0031324~negative regulation of cellular metabolic process	22	2.28E-04	MSH6, NANOG, TBX3, JARID2, NODAL, SOX2, TLE4, REST, NR0B1, SIRT1, FXR1, TCFCP2L1, CDT1, SUZ12, SAP30, EIF4EBP2, STRA8, POU5F1, SOX15, INPP5D, RBPJ, MYST4
GOTERM_BP_5	GO:0045934~negative regulation of nucleobase, nucleoside, nucleotide and nucleic acid metabolic process	19	3.34E-04	MSH6, NANOG, TBX3, JARID2, NODAL, SOX2, TLE4, REST, NR0B1, SIRT1, TCFCP2L1, CDT1, SUZ12, SAP30, STRA8, POU5F1, SOX15, RBPJ, MYST4
GOTERM_BP_5	GO:0051172~negative regulation of nitrogen compound metabolic process	19	3.83E-04	MSH6, NANOG, TBX3, JARID2, NODAL, SOX2, TLE4, REST, NR0B1, SIRT1, TCFCP2L1, CDT1, SUZ12, SAP30, STRA8, POU5F1, SOX15, RBPJ, MYST4
GOTERM_CC_5	GO:0000228~nuclear chromosome	9	0.001313581	SUZ12, SAP30, MSH6, DNMT3L, EED, STAG3, SYCE1, MLH3, DMC1
GOTERM_BP_5	GO:0010629~negative regulation of gene expression	18	0.001343787	NANOG, TBX3, JARID2, NODAL, SOX2, TLE4, REST, NR0B1, SIRT1, TCFCP2L1, SUZ12, SAP30, MOV10, POU5F1, SOX15, PIWIL2, RBPJ, MYST4
GOTERM_BP_5	GO:0045892~negative regulation of transcription, DNA-dependent	15	0.001508769	NANOG, TBX3, JARID2, NODAL, SOX2, TLE4, REST, NR0B1, SIRT1, TCFCP2L1, SUZ12, SAP30, POU5F1, SOX15, RBPJ
GOTERM_BP_5	GO:0051253~negative regulation of RNA metabolic process	15	0.001585979	NANOG, TBX3, JARID2, NODAL, SOX2, TLE4, REST, NR0B1, SIRT1, TCFCP2L1, SUZ12, SAP30, POU5F1, SOX15, RBPJ
GOTERM_MF_5	GO:0032559~adenyl ribonucleotide binding	43	0.002180167	XRCC5, STK31, INO80, TRIB3, ASNS, MLH3, STK30, SETX, IRAK3, TOR3A, ANKRD17, MOV10, ETNK1, DHX16, NLRP4A, UBE1Y1, IPMK, NLRP4F, DHX32, MSH6, HSP90AA1, TWF2, PIK3CB, HCK, PDK4, PIM1, PFKP, PIM3, SYKB, AK7, MORC1, RPS6KA5, TEX14, P2RX7, ABCB1A, ABCB1B, DNAHC8, D1PAS1, TESK2, ABCC4, OAS1A, DMC1, CAMK1D
GOTERM_CC_5	GO:0044454~nuclear chromosome part	8	0.002232589	SUZ12, SAP30, MSH6, DNMT3L, EED, STAG3, SYCE1, MLH3
GOTERM_BP_5	GO:0006355~regulation of transcription, DNA-dependent	42	0.003063501	SOX2, GM13138, GM13139, REST, ZIC3, GM13212, TCFCP2L1, NFATC2IP, SAP30, STAT4, ZFP819, TCEA3, POU5F1, GBX2, SOX15, ETV5, MAFF, UTF1, NANOG, KAT2B, REX2, TBX3, JARID2, ESRRB, TCFAP2C, NODAL, I1C0022H11RIK, DMRT1,

				TLE4, ZFP59, NR0B1, SIRT1, CPHX, RPS6KA5, SUZ12, GM13152, 1700029I01RIK, ZFP655, RHOX13, ZFP534, RFX2, 2610305D13RIK, RBPJ, NR5A2
GOTERM_BP_5	GO:0016481~negative regulation of transcription	16	0.003305391	NANOG, TBX3, JARID2, NODAL, SOX2, TLE4, REST, NR0B1, SIRT1, TCFCP2L1, SUZ12, SAP30, POU5F1, SOX15, RBPJ, MYST4
**GOTERM_BP_5**	**GO:0007127~meiosis I**	**5**	**0.003572703**	**STRA8, PIWIL2, SYCE1, MLH3, DMC1**
**GOTERM_BP_5**	**GO:0007498~mesoderm development**	**6**	**0.004274789**	**NANOG, TBX3, NF2, POU5F1, NODAL, TCF15**
**GOTERM_BP_5**	**GO:0048332~mesoderm morphogenesis**	**5**	**0.00482775**	**NANOG, TBX3, NF2, POU5F1, NODAL**
GOTERM_BP_5	GO:0051327~M phase of meiotic cell cycle	7	0.005289194	CLGN, STRA8, STAG3, PIWIL2, SYCE1, MLH3, DMC1
**GOTERM_BP_5**	**GO:0007126~meiosis**	**7**	**0.005289194**	**CLGN, STRA8, STAG3, PIWIL2, SYCE1, MLH3, DMC1**
GOTERM_BP_5	GO:0048477~oogenesis	5	0.005304159	STRA8, PIWIL2, DAZL, DMC1, SOHLH2
GOTERM_CC_5	GO:0000794~condensed nuclear chromosome	5	0.006138177	DNMT3L, STAG3, SYCE1, MLH3, DMC1
GOTERM_CC_5	GO:0005694~chromosome	15	0.006997898	XRCC5, MSH6, KAT2B, SYCE1, MLH3, CBX7, SUZ12, SAP30, RIF1, DNMT3L, CENPV, EED, STAG3, DMC1, MYST4
GOTERM_BP_5	GO:0043009~chordate embryonic development	16	0.009961739	MAFF, COBL, ENAH, TBX3, IFITM1, OOEP, ESRRB, NODAL, TTPA, POU5F1, DNMT3L, GBX2, HBEGF, PRDM1, IPMK, TCF15
GOTERM_CC_5	GO:0044427~chromosomal part	13	0.0105535	MSH6, KAT2B, SYCE1, MLH3, CBX7, SUZ12, SAP30, RIF1, DNMT3L, CENPV, EED, STAG3, MYST4
GOTERM_BP_5	GO:0016568~chromatin modification	11	0.011134915	SUZ12, PHF17, KAT2B, EED, CENPV, SMYD3, INO80, KDM3B, CBX7, SIRT1, MYST4
GOTERM_BP_5	GO:0045137~development of primary sexual characteristics	7	0.011674255	NANOG, TBX3, STRA8, SOX2, DMC1, NR0B1, SIRT1
GOTERM_BP_5	GO:0009887~organ morphogenesis	20	0.011979365	COBL, NANOG, ENAH, TBX3, NF2, PDGFA, NODAL, ARID5B, SOX2, FEM1B, ZIC3, BBS2, P2RX7, CHRNA9, POU5F1, GBX2, IPMK, ETV5, TCF15, FGF4
GOTERM_BP_5	GO:0008284~positive regulation of cell proliferation	12	0.014673214	SUZ12, NANOG, TBX3, PDGFA, NODAL, SOX2, ST8SIA1, HBEGF, SYKB, RBPJ, FGFBP1, FGF4
GOTERM_CC_5	GO:0000790~nuclear chromatin	5	0.015680166	SUZ12, SAP30, MSH6, DNMT3L, EED
GOTERM_BP_5	GO:0040015~negative regulation of multicellular organism growth	3	0.016228805	ADRB3, BBS2, SOCS2
GOTERM_BP_5	GO:0006464~protein modification process	34	0.019985524	STK31, FUT9, CDC14A, ST8SIA1, TRIB3, STK30, IRAK3, STAT4, EED, CRY1, UBE1Y1, MYST4, FGD4, KAT2B, OOEP, HCK, PDK4, PIM1, HERC4, PIM3, SYKB, SIRT1, AVPI1, ADPRH, SUZ12, RPS6KA5, PHF17, TEX14,

				P2RX7, ST8SIA6, DUSP27, TESK2, PIAS2, CAMK1D
GOTERM_BP_5	GO:0007128~meiotic prophase I	3	0.022473651	PIWIL2, SYCE1, DMC1
GOTERM_BP_5	GO:0051324~prophase	3	0.022473651	PIWIL2, SYCE1, DMC1
KEGG_PATHWAY	mmu04070:Phosphatidylinositol signaling system	5	0.02366407	CDS2, IMPA2, PIK3CB, INPP5D, CDS1
GOTERM_CC_5	GO:0005844~polysome	3	0.02428234	PIWIL2, DAZL, NR0B1
GOTERM_MF_5	GO:0003690~double-stranded DNA binding	4	0.026754118	ANKRD17, MSH6, MLH3, DMC1
**GOTERM_BP_5**	**GO:0001707~mesoderm formation**	**4**	**0.027214202**	**NANOG, NF2, POU5F1, NODAL**
GOTERM_BP_5	GO:0006644~phospholipid metabolic process	8	0.02932668	CDS2, SMPDL3B, PIK3CB, FABP3, PITPNC1, CDS1, IPMK, PLA2G5
GOTERM_BP_5	GO:0007143~female meiosis	3	0.029543036	STRA8, STAG3, MLH3
GOTERM_BP_5	GO:0001829~trophectodermal cell differentiation	3	0.033365865	ESRRB, POU5F1, NODAL
GOTERM_CC_5	GO:0005654~nucleoplasm	18	0.03543774	KAT2B, SOX2, CALCOCO2, REST, SIRT1, COIL, SETX, TCFCP2L1, SUZ12, SAP30, PHF17, SPNB4, ASH2L, 2210409E12RIK, POU5F1, EED, PIAS2, RBPJ, MYST4
**GOTERM_BP_5**	**GO:0001704~formation of primary germ layer**	**4**	**0.035732565**	**NANOG, NF2, POU5F1, NODAL**
GOTERM_BP_5	GO:0007263~nitric oxide mediated signal transduction	2	0.036014147	MT2, MT1
**GOTERM_BP_5**	**GO:0007281~germ cell development**	**6**	**0.036859338**	**BBS2, RNF17, PRDM14, STRA8, DAZL, DMC1**
GOTERM_BP_5	GO:0009968~negative regulation of signal transduction	8	0.03689785	IRAK3, P2RX7, NANOG, SOCS2, STMN3, NF2, SOX2, INPP5D
GOTERM_CC_5	GO:0044451~nucleoplasm part	16	0.037173306	KAT2B, SOX2, CALCOCO2, REST, COIL, TCFCP2L1, SUZ12, SAP30, PHF17, SPNB4, ASH2L, 2210409E12RIK, POU5F1, EED, PIAS2, RBPJ, MYST4
GOTERM_BP_5	GO:0007129~synapsis	3	0.037370021	STRA8, SYCE1, MLH3
GOTERM_BP_5	GO:0070192~chromosome organization involved in meiosis	3	0.037370021	STRA8, SYCE1, MLH3
GOTERM_BP_5	GO:0009948~anterior/posterior axis specification	3	0.037370021	TBX3, NODAL, PCSK6
GOTERM_CC_5	GO:0045120~pronucleus	3	0.038999513	RIF1, EED, TCL1
GOTERM_CC_5	GO:0000793~condensed chromosome	6	0.04015412	DNMT3L, CENPV, STAG3, SYCE1, MLH3, DMC1
GOTERM_BP_5	GO:0009888~tissue development	19	0.042623898	COBL, NANOG, ENAH, TBX3, NF2, PDGFA, NODAL, MYLPF, MREG, FEM1B, TCFCP2L1, ANKRD17, POU5F1, GBX2, SOX15, RBPJ, IPMK, TCF15, FGF4
GOTERM_MF_5	GO:0003714~transcription corepressor activity	5	0.04476823	POU5F1, TLE4, TRIB3, NR0B1, SIRT1
GOTERM_BP_5	GO:0008652~cellular amino acid biosynthetic process	4	0.045459696	BCAT1, CTH, 4933437F05RIK, ASNS

**GOTERM_BP_5**	**GO:0007140~male meiosis**	**3**	**0.045891377**	**STAG3, MLH3, DMC1**
GOTERM_CC_5	GO:0000795~synaptonemal complex	3	0.047339706	STAG3, SYCE1, MLH3
KEGG_PATHWAY	mmu00601:Glycosphingolipid biosynthesis	3	0.047465753	GCNT2, FUT9, ST8SIA1
GOTERM_BP_5	GO:0051053~negative regulation of DNA metabolic process	3	0.050393659	MSH6, STRA8, CDT1
GOTERM_CC_5	GO:0035098~ESC/E(Z) complex	2	0.051720749	SUZ12, EED
GOTERM_BP_5	GO:0030258~lipid modification	4	0.053525324	ACOXL, PIK3CB, BDH2, IPMK
GOTERM_BP_5	GO:0001831~trophectodermal cellular morphogenesis	2	0.05353402	ESRRB, NODAL
GOTERM_BP_5	GO:0051900~regulation of mitochondrial depolarization	2	0.05353402	P2RX7, GCLM
GOTERM_BP_5	GO:0030718~germ-line stem cell maintenance	2	0.05353402	POU5F1, PIWIL2
GOTERM_BP_5	GO:0010648~negative regulation of cell communication	8	0.053691733	IRAK3, P2RX7, NANOG, SOCS2, STMN3, NF2, SOX2, INPP5D
GOTERM_BP_5	GO:0019752~carboxylic acid metabolic process	15	0.053873516	BCAT1, ACOXL, 4933437F05RIK, PDK4, GOT1L1, EPHX2, ASNS, SYKB, GCLM, PIPOX, CTH, FOLR1, AS3MT, BDH2, NR5A2
GOTERM_MF_5	GO:0008559~xenobiotic-transporting ATPase activity	2	0.054737289	ABCB1A, ABCB1B
GOTERM_BP_5	GO:0000902~cell morphogenesis	11	0.054875142	BBS2, P2RX7, ENAH, PRDM14, OOEP, SPNB4, ESRRB, NODAL, GBX2, RBPJ, TCFCP2L1
GOTERM_BP_5	GO:0001701~in utero embryonic development	10	0.054968222	MAFF, TBX3, OOEP, ESRRB, POU5F1, NODAL, DNMT3L, TTPA, HBEGF, PRDM1
GOTERM_CC_5	GO:0043073~germ cell nucleus	3	0.0562624	2210409E12RIK, STAG3, MLH3, COIL
GOTERM_BP_5	GO:0001825~blastocyst formation	3	0.059845715	ESRRB, POU5F1, NODAL
GOTERM_BP_5	GO:0009952~anterior/posterior pattern formation	7	0.060464577	TBX3, IFITM1, NODAL, GBX2, PCSK6, ZIC3, TCF15
GOTERM_CC_5	GO:0034708~methyltransferase complex	3	0.060926368	SUZ12, ASH2L, EED
GOTERM_CC_5	GO:0035097~histone methyltransferase complex	3	0.060926368	SUZ12, ASH2L, EED
GOTERM_BP_5	GO:0043549~regulation of kinase activity	8	0.061591894	IRAK3, P2RX7, NF2, TRIB3, SYKB, GADD45A, AVPI1, FGD4
GOTERM_BP_5	GO:0016310~phosphorylation	20	0.063963527	STK31, OOEP, PIK3CB, HCK, PDK4, PIM1, TRIB3, SYKB, PIM3, STK30, AVPI1, RPS6KA5, IRAK3, TEX14, STAT4, P2RX7, TESK2, IPMK, FGD4, CAMK1D
GOTERM_BP_5	GO:0021915~neural tube development	5	0.064302881	COBL, ENAH, NODAL, GBX2, IPMK
GOTERM_BP_5	GO:0044267~cellular protein metabolic process	48	0.064722664	MKRN1, STK31, FUT9, CDC14A, ST8SIA1, TRIB3, STK30, FEM1B, PSMA8, IRAK3, TOR3A, STAT4, RPL39L, EED, FBXO6, CRY1, UBE1Y1, MYST4, FGD4, HSP90AA1, KAT2B, SOCS2, OOEP, HCK, PDK4, PIM1,

				HERC4, PIM3, SYKB, TRIM63, SIRT1, AVPI1, ADPRH, RPS6KA5, SUZ12, USP28, PHF17, TEX14, P2RX7, CLGN, ST8SIA6, DUSP27, TESK2, RNF138, PIAS2, USP48, FBXO15, CAMK1D
GOTERM_BP_5	GO:0001841~neural tube formation	4	0.065266174	COBL, ENAH, NODAL, IPMK
GOTERM_BP_5	GO:0010604~positive regulation of macromolecule metabolic process	18	0.06931915	UTF1, KAT2B, TBX3, NODAL, SOX2, SYKB, ZIC3, P2RX7, POU5F1, GPX4, SOX15, PIWIL2, DAZL, PRDM1, KLF2, NR5A2, ETV5, MYST4
GOTERM_BP_5	GO:0048729~tissue morphogenesis	9	0.069416462	COBL, NANOG, ENAH, TBX3, NF2, POU5F1, NODAL, FEM1B, IPMK
GOTERM_BP_5	GO:0006749~glutathione metabolic process	3	0.069849178	GPX4, GCLM, GSTP1
GOTERM_BP_5	GO:0007066~female meiosis sister chromatid cohesion	2	0.070736882	STRA8, STAG3
GOTERM_CC_5	GO:0000785~chromatin	7	0.070761739	SUZ12, SAP30, MSH6, DNMT3L, EED, CBX7, MYST4
GOTERM_BP_5	GO:0051348~negative regulation of transferase activity	4	0.071542914	IRAK3, NF2, TRIB3, GADD45A
GOTERM_BP_5	GO:0000279~M phase	10	0.073610208	CLGN, STRA8, CENPV, STAG3, PIWIL2, SYCE1, HORMAD1, CEP55, MLH3, DMC1
GOTERM_BP_5	GO:0001890~placenta development	5	0.07374834	ESRRB, NODAL, TTPA, GJB3, PRDM1
KEGG_PATHWAY	mmu00564:Glycerophospholipid metabolism	4	0.074354789	CDS2, ETNK1, CDS1, PLA2G5
GOTERM_BP_5	GO:0001838~embryonic epithelial tube formation	4	0.074779348	COBL, ENAH, NODAL, IPMK
GOTERM_BP_5	GO:0035282~segmentation	4	0.074779348	TBX3, IFITM1, PCSK6, TCF15
GOTERM_BP_5	GO:0010741~negative regulation of protein kinase cascade	3	0.07504145	P2RX7, SOCS2, NF2
GOTERM_BP_5	GO:0008406~gonad development	5	0.07621406	NANOG, STRA8, DMC1, NR0B1, SIRT1
GOTERM_BP_5	GO:0006650~glycerophospholipid metabolic process	5	0.07621406	PIK3CB, FABP3, PITPNC1, CDS1, IPMK
GOTERM_BP_5	GO:0031328~positive regulation of cellular biosynthetic process	16	0.076669537	UTF1, KAT2B, HSP90AA1, TBX3, NODAL, SOX2, SYKB, ZIC3, POU5F1, SOX15, PIWIL2, DAZL, KLF2, NR5A2, ETV5, MYST4
GOTERM_BP_5	GO:0031325~positive regulation of cellular metabolic process	18	0.078394209	UTF1, KAT2B, HSP90AA1, TBX3, NODAL, SOX2, SYKB, ZIC3, P2RX7, POU5F1, GPX4, SOX15, PIWIL2, DAZL, KLF2, NR5A2, ETV5, MYST4
KEGG_PATHWAY	mmu00260:Glycine, serine and threonine metabolism	3	0.079182159	CTH, TDH, PIPOX
GOTERM_BP_5	GO:0001947~heart looping	3	0.080352468	TBX3, NODAL, ZIC3
GOTERM_CC_5	GO:0000123~histone acetyltransferase complex	3	0.080794513	PHF17, KAT2B, MYST4
GOTERM_BP_5	GO:0009891~positive regulation of biosynthetic process	16	0.083190158	UTF1, KAT2B, HSP90AA1, TBX3, NODAL, SOX2, SYKB, ZIC3, POU5F1, SOX15, PIWIL2, DAZL, KLF2, NR5A2, ETV5, MYST4

GOTERM_BP_5	GO:0046545~development of primary female sexual characteristics	4	0.084868024	TBX3, STRA8, DMC1, SIRT1
GOTERM_BP_5	GO:0046486~glycerolipid metabolic process	6	0.086092561	PIK3CB, FABP3, PITPNC1, CDS1, IPMK, SIRT1
GOTERM_BP_5	GO:0046851~negative regulation of bone remodeling	2	0.087628444	P2RX7, INPP5D
GOTERM_BP_5	GO:0045779~negative regulation of bone resorption	2	0.087628444	P2RX7, INPP5D
GOTERM_BP_5	GO:0051177~meiotic sister chromatid cohesion	2	0.087628444	STRA8, STAG3
GOTERM_BP_5	GO:0035148~tube lumen formation	4	0.091898749	COBL, ENAH, NODAL, IPMK
GOTERM_BP_5	GO:0009966~regulation of signal transduction	18	0.093912182	FGD1, NANOG, NF2, STMN3, SOCS2, MCF2, SOX2, TRIB3, SYKB, AVPI1, IRAK3, ADRB3, P2RX7, RASGRP2, INPP5D, FGFBP1, KNDC1, FGD4
GOTERM_BP_5	GO:0007398~ectoderm development	6	0.095037858	NF2, POU5F1, PDGFA, RBPJ, MREG, TCF15
GOTERM_BP_5	GO:0044106~cellular amine metabolic process	9	0.095484198	BCAT1, CTH, FOLR1, 4933437F05RIK, GOT1L1, FABP3, ASNS, GCLM, D14ERTD668E
GOTERM_BP_5	GO:0007492~endoderm development	3	0.096939023	ANKRD17, POU5F1, NODAL
GOTERM_BP_5	GO:0001892~embryonic placenta development	4	0.099162614	ESRRB, NODAL, TTPA, PRDM1

Supplementary Table S2 . qRT-PCR has been performed using the following primers

Gene	Forward Primer	Reverse Primer
**Mageb16**	AATGGGAATCTATGCTGGGATGA	TCACTATTGGCTATTGGCTGGTA
**Tnnt2**	CAGAGGAGGCCAACGTAGAAG	CTCCATCGGGGATCTTGGGT
**Esrrb**	GCACCTGGGCTCTAGTTGC	TACAGTCCTCGTAGCTCTTGC
**Zeb2**	ATTGCACATCAGACTTTGAGGAA	ATAATGGCCGTGTCGCTTCG
**Vim**	CGTCCACACGCACCTACAG	GGGGGATGAGGAATAGAGGCT
**Gapdh**	AGGTCGGTGTGAACGGATTTG	TGTAGACCATGTAGTTGAGGTCA
